# Supplementary material for: Transcriptome-wide decoding the roles of aberrant splicing in melanoma MAPK-targeted resistance evolution
Source: EMBO Rep. 2025 Jul 18;26(16):4180–96. doi: 10.1038/s44319-025-00521-6 (PMC12373858; doi:10.1038/s44319-025-00521-6)
Supplement: Supplementary file 6 — Appendix [file 44319_2025_521_MOESM6_ESM.pdf]

# Appendix

Appendix Figure S1 ... 2
Appendix Figure S2 ... 3
Appendix Figure S3 ... 4
Appendix Figure S4 ... 5
Appendix Figure S5 ... 6
Appendix Figure S6 ... 7
Appendix Figure S7 ... 8
Appendix Figure S8 ... 9
Appendix Figure S9 ... 10
Appendix Figure S10 ... 11
Appendix Figure S11 ... 12
Appendix Figure S12 ... 13
Appendix Figure S13 ... 14
Appendix Figure S14 ... 15
Appendix Figure S15 ... 16
Appendix Figure S16 ... 17
Appendix Figure S17 ... 18

**Appendix Figure S1**

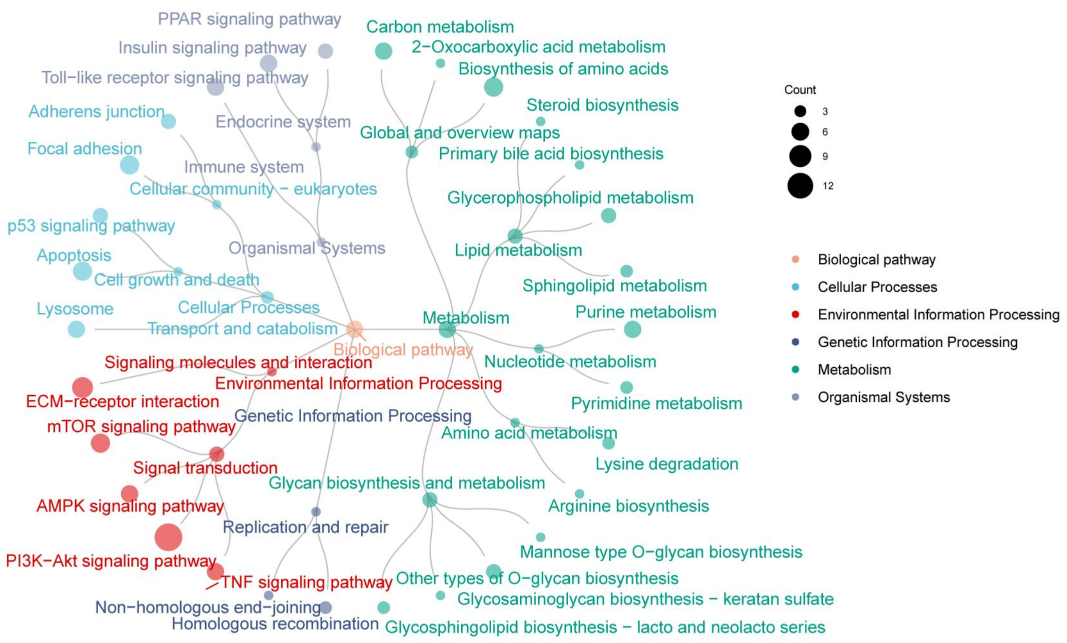


**Appendix Figure S1. KEGG pathway enrichment of differential splicing events.** The circular dendrogram shows KEGG pathways that are significantly enriched for genes undergoing differential splicing events. The size of circles represents the number of genes belonging to that pathway.

## Appendix Figure S2

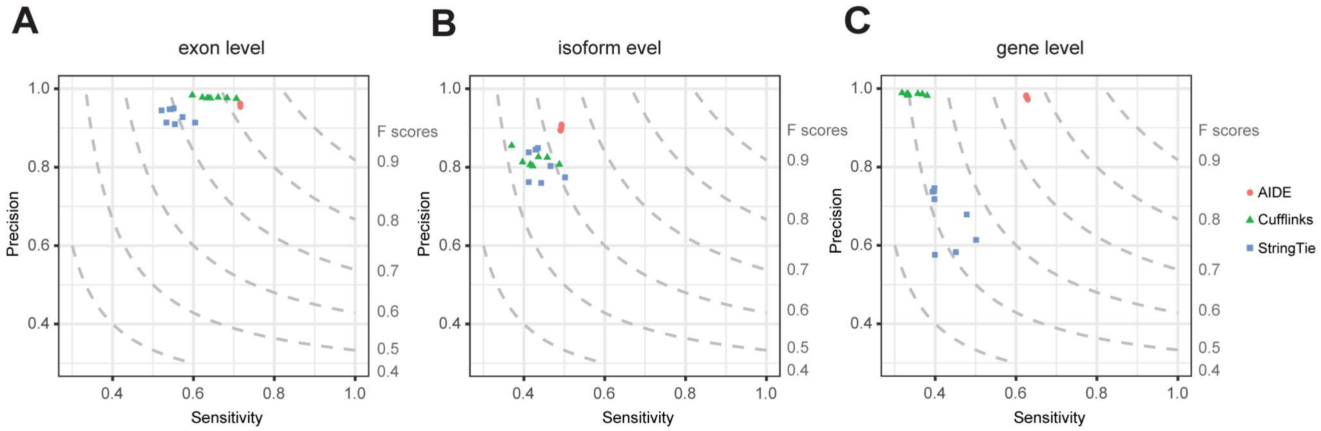


**Appendix Figure S2. Comparison between AIDE and the other isoform discovery methods in RNAseq data.** We applied AIDE, Cufflinks, and StringTie for isoform discovery. The exon-level (A), isoform-level (B), and gene-level (C) precision rates, sensitivity, and F scores averaged across the human genes were calculated.

**Appendix Figure S3**

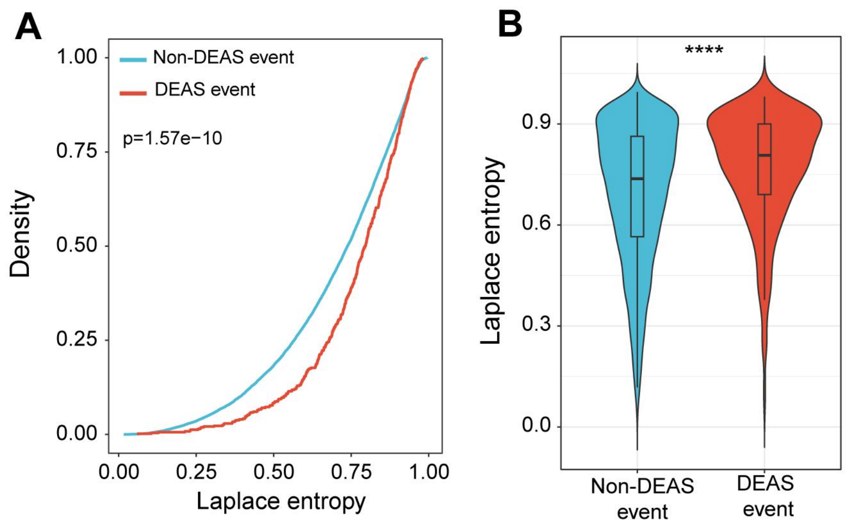


**Appendix Figure S3. The robustness of splicing changes across all significant events.** (A) The cumulative curve distribution of Laplace entropy between genes that undergo differential splicing events and genes that do not undergo differential splicing events. Kolmogorov-Smirnov test. (B) The boxplot shows the Laplace entropy between genes that undergo differential splicing events and genes that do not undergo differential splicing events. Student's t-test. **** $p < 0.0001$.

## Appendix Figure S4

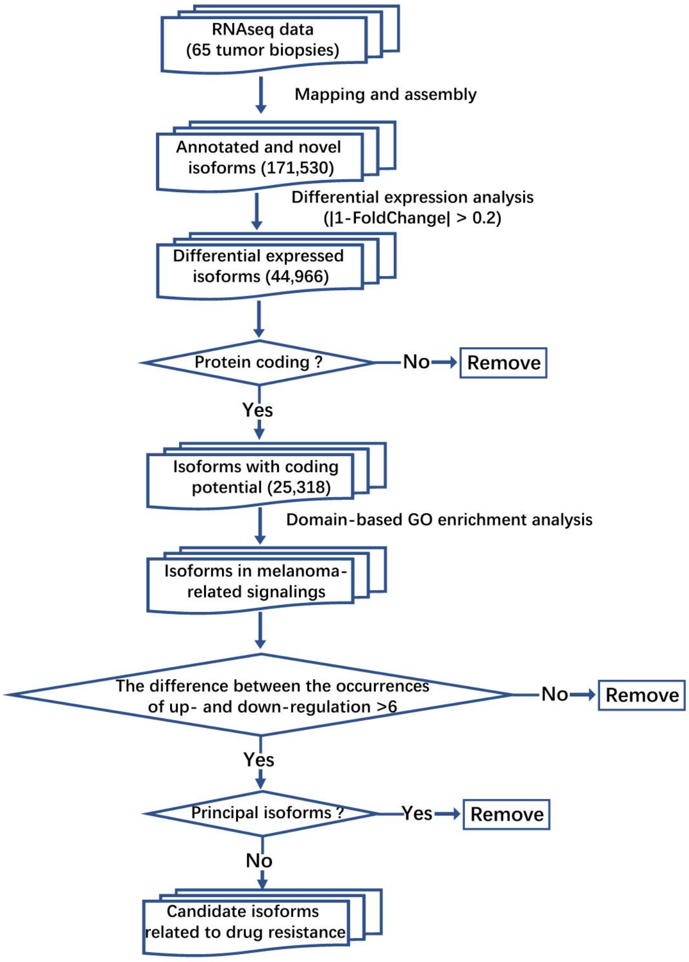


**Appendix Figure S4. Screening process for functional splicing isoforms associated with resistance to MAPK-targeted therapy.** First, we screened differentially expressed splicing isoforms based on the fold change in expression between paired samples, with a |1-FoldChange| > 0.2 and a minimum expression exceeding 10 in Baseline or DP groups. Subsequently, isoforms with protein-coding potential were screened based on the functional characteristics of the isoforms in the Ensembl database. Next, aberrant isoforms enriched in melanoma-associated pathways were identified through domain-based GO pathway enrichment analysis. Isoforms repeatedly upregulated or downregulated in MAPKi-resistant samples were selected from the aforementioned isoform sets (the absolute difference between the upregulated and downregulated paired samples exceeded 6). Finally, based on annotations from the APPRIS database, the principal isoforms were excluded, and candidate isoforms related to drug resistance were identified.

## Appendix Figure S5

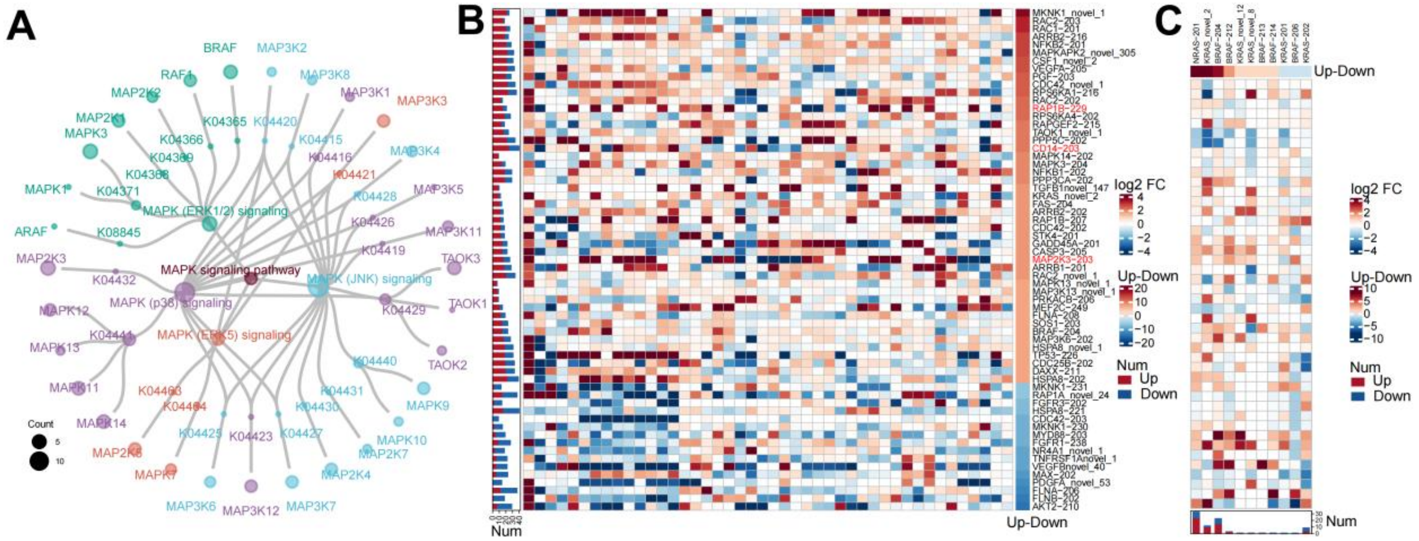


**Appendix Figure S5. MAPKi induces dramatic splicing changes in the MAPK signaling pathway.** (A) Differentially expressed splicing isoforms of four functional modules in the MAPK pathway. The color of the dots represents different modules. The size of the outermost dots represents the number of differentially expressed splicing isoforms, the size of the middle layer dots represents the number of genes within each KEGG Orthology group, and the size of the innermost dots represents the number of genes within different MAPK functional modules. (B) Expression patterns of differentially expressed splicing isoforms in the MAPK pathway. Only non-principal isoforms with high occurrence are shown. Splicing isoforms are ranked based on the difference between the number of upregulation and downregulation events, with the corresponding color bar on the right. The stacked bar chart in the left panel shows the upregulation (red) and downregulation (blue) events of each splicing isoform. (C) Aberrant splicing isoforms of the oncogenes BRAF, NRAS, and KRAS are illustrated in the heatmap.

## Appendix Figure S6

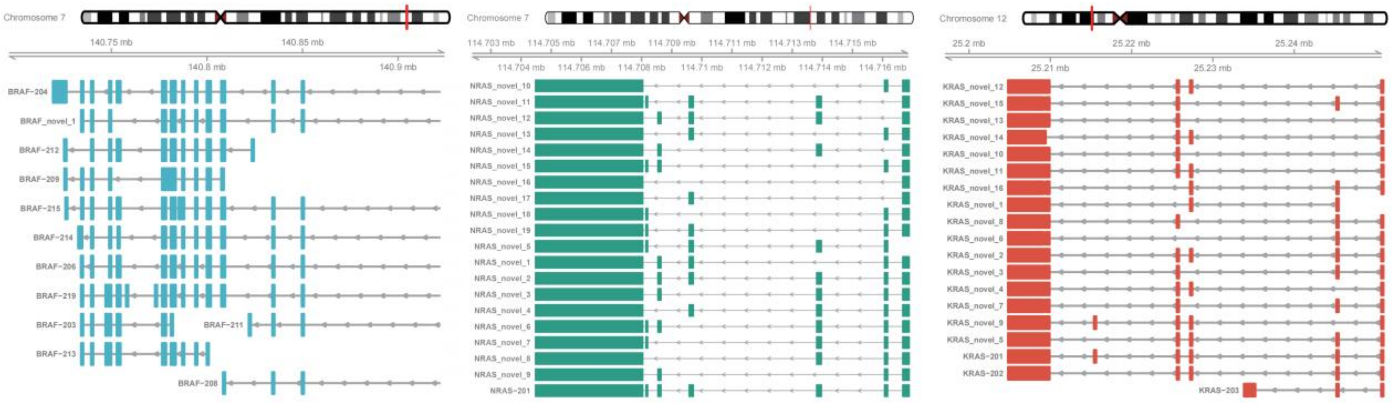


**Appendix Figure S6. Exon structure of BRAF, NRAS, and KRAS isoforms.**

**Appendix Figure S7**

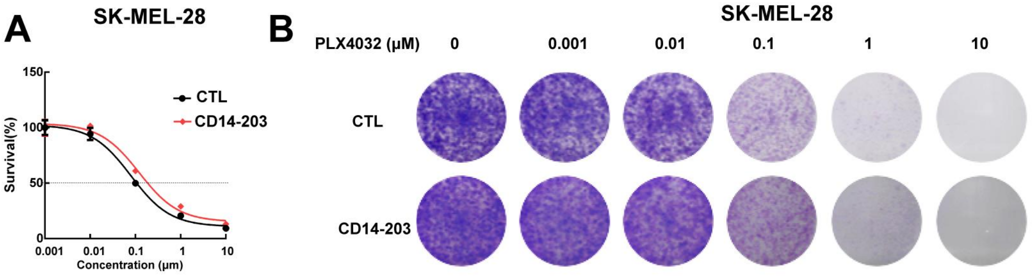


**Appendix Figure S7. The aberrant splicing of CD14 confers MAPKi resistance in melanoma cells.** (A) Short-term cell viability assay in melanoma cells with CD14-203 overexpression after PLX4032 treatment. The data are shown as mean±SD (n=5, biological replicates); normalized to DMSO vehicle as 100%. (B) Colony formation assay in melanoma cells with CD14-203 overexpression, followed by treatment as indicated.

## Appendix Figure S8

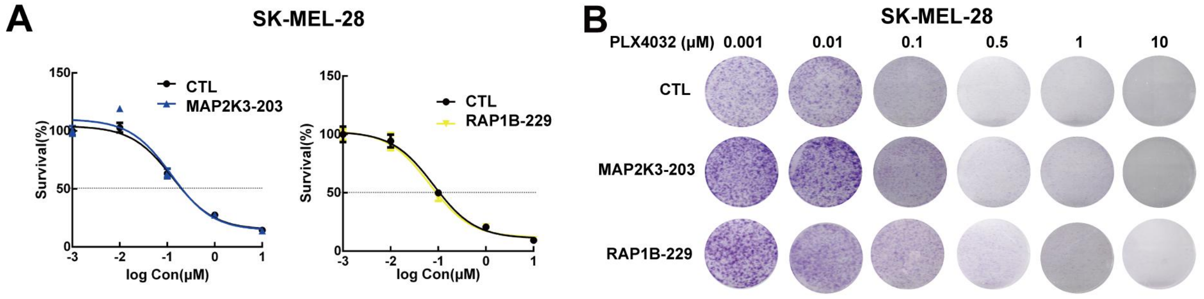


**Appendix Figure S8. Overexpression of MAP2K3-203 and RAP1B-229 in melanoma cells.** (A) Cell viability assays in melanoma cells with MAP2K3-203 and RAP1B-229 overexpression after PLX4032 treatment. The data are shown as mean±SD (n=5, biological replicates); normalized to DMSO vehicle as 100%. (B) Colony formation assay in melanoma cells with MAP2K3-203 and RAP1B-229 overexpression, followed by treatment as indicated.

**Appendix Figure S9**

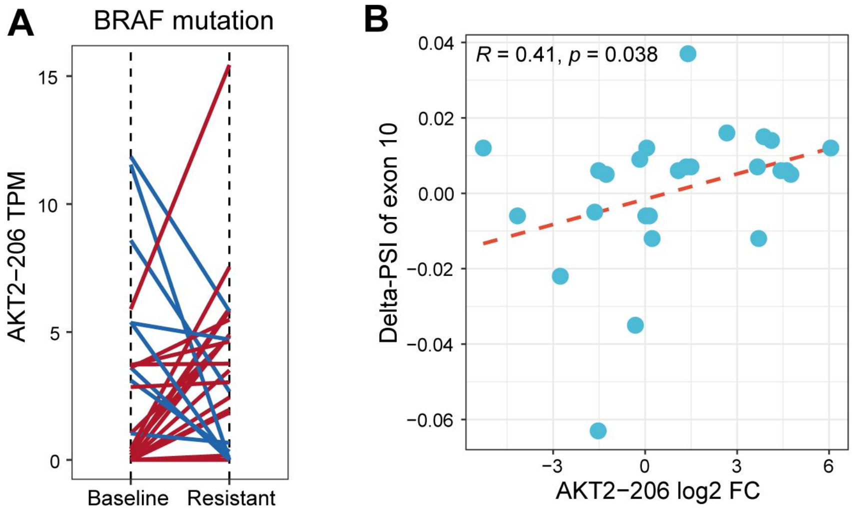


**Appendix Figure S9. Expression patterns of AKT2-206**. (A) AKT2-206 expression changes in five independent BRAF mutant melanoma datasets under MAPKi (GSE75299, GSE203545, GSE285131, GSE103630, GSE186108). (B) Scatter plot showing Spearman's correlation between log2FC of AKT2-206 isoforms and Delta-PSI of exon 10.

**Appendix Figure S10**

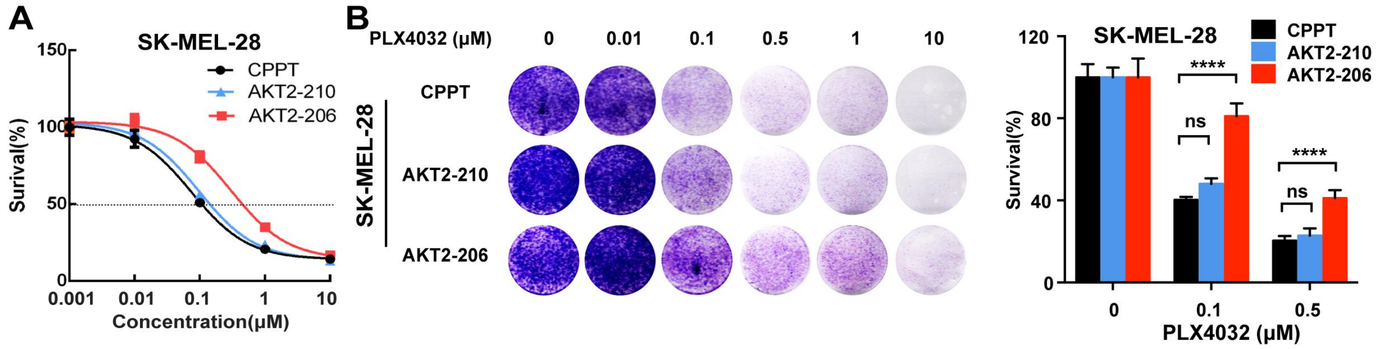


**Appendix Figure S10. Overexpression of AKT2-206 enhances drug resistance to MAPKi.** (A) Cell viability assays in melanoma cells with AKT2 isoforms overexpression under PLX4032 treatment. The data are shown as mean±SD (n=5, biological replicates); normalized to DMSO vehicle as 100%. (B) Colony formation assay in melanoma cells with AKT2 isoforms overexpression. The data are presented as mean ± SD (n = 3, biological replicates); ANOVA. ns $p > 0.05$, **** $p < 0.0001$.

## Appendix Figure S11

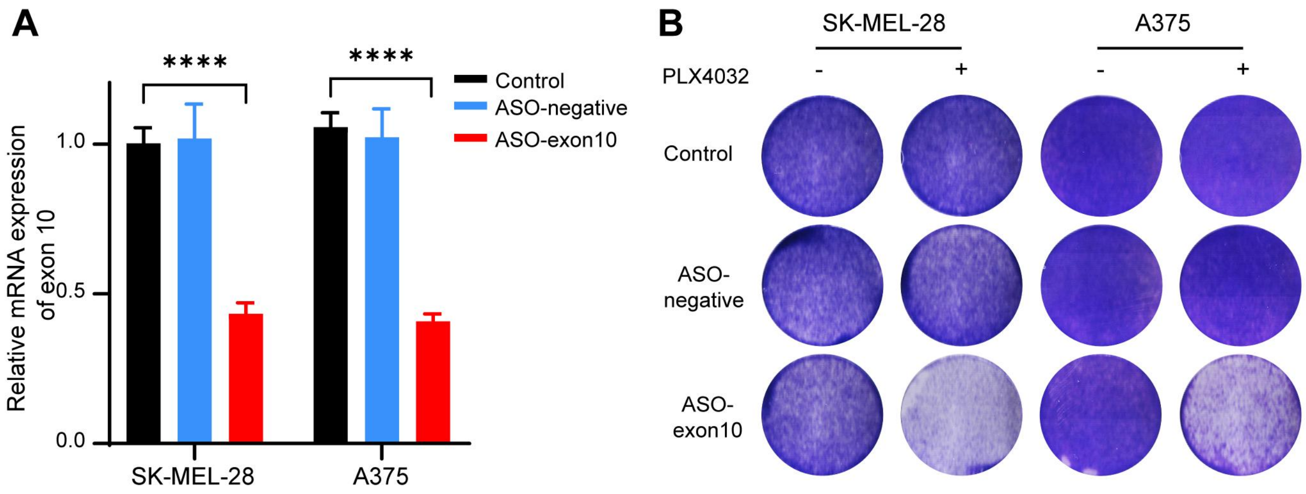


**Appendix Figure S11. ASO-mediated exon 10 silencing increased the sensitivity of melanoma cells to BRAFi.** (A) Efficiency of ASO-mediated exon 10 silencing. The data are shown as mean±SD (n=3, biological replicates); ANOVA. (B) Colony formation assay was performed on melanoma cells transfected with negative ASO control and exon 10 ASO with or without PLX4032 (0.01μM). **** $p < 0.0001$.

**Appendix Figure S12**

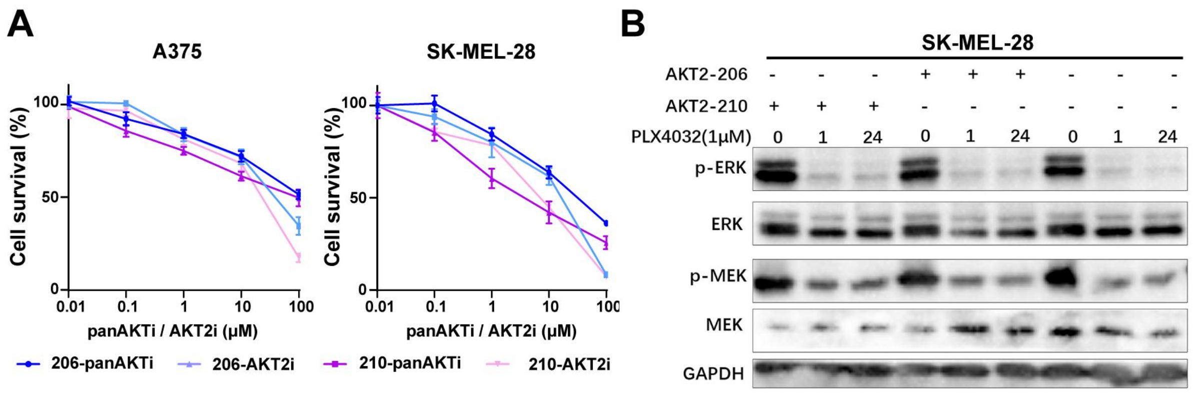


**Appendix Figure S12. Functional Characterization of AKT2 Splicing Isoforms in Melanoma Drug Responses and MAPK Signaling.** (A) Cell viability assays in melanoma cells with AKT2-206 and AKT2-210 overexpression after AKT2-specific inhibitor CCT128930 and pan-AKT inhibitor capivasertib treatment. The data are shown as mean±SD (n=5, biological replicates). (B) The inhibitory effect of PLX4032 on MAPK signaling upon the overexpression of AKT2 splicing isoforms. The activation levels of MEK and ERK were analyzed by western blotting.

## Appendix Figure S13

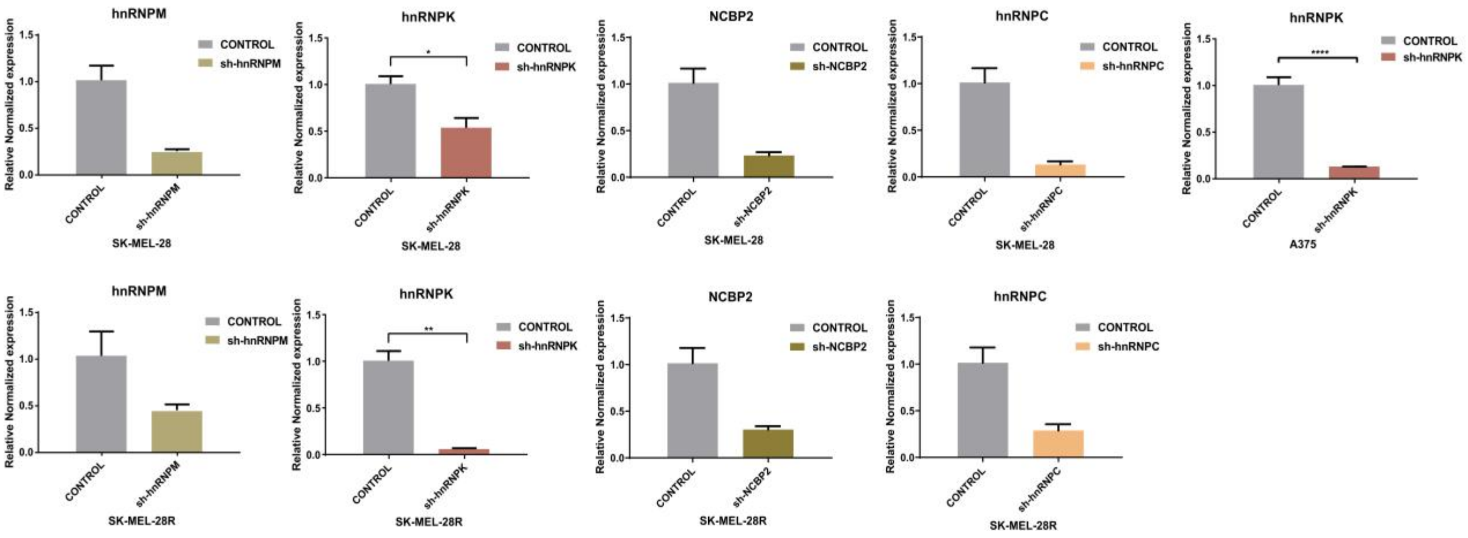


**Appendix Figure S13. The efficiency of knockdown using RNA interference.** A375, SK-MEL-28 resistant and parental cell lines were individually transduced with shRNA directed against RNA-binding proteins or control shRNA. Knockdown efficiency was assessed through qRT-PCR relative to the expression of the housekeeping gene. The data are shown as mean±SD (n=3, biological replicates); Student's t-test. * $p < 0.05$, ** $p < 0.01$, **** $p < 0.0001$.

**Appendix Figure S14**

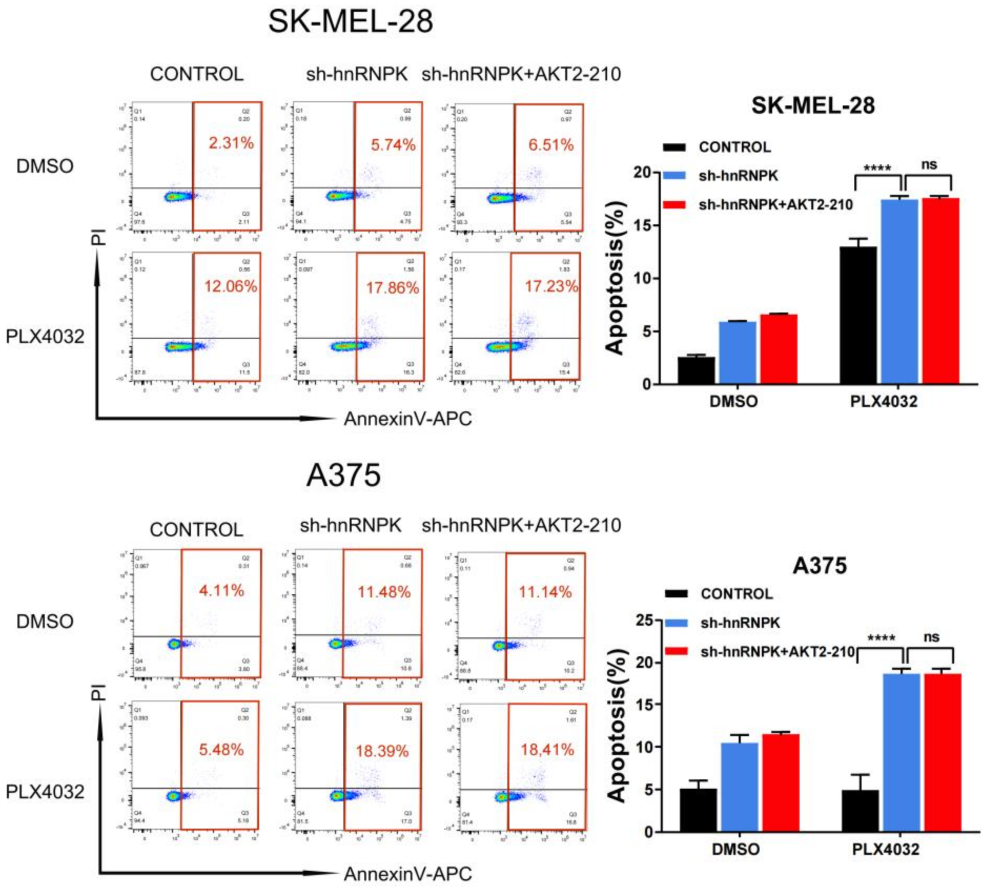


**Appendix Figure S14. The effect of AKT2-210 overexpression on cell apoptosis.** Cell apoptosis induced by PLX4032 (1μM, 96h) was detected by annexin V/PI staining. The data are shown as mean±SD (n=3, biological replicates); ANOVA. ns $p > 0.05$, **** $p < 0.0001$.

## Appendix Figure S15

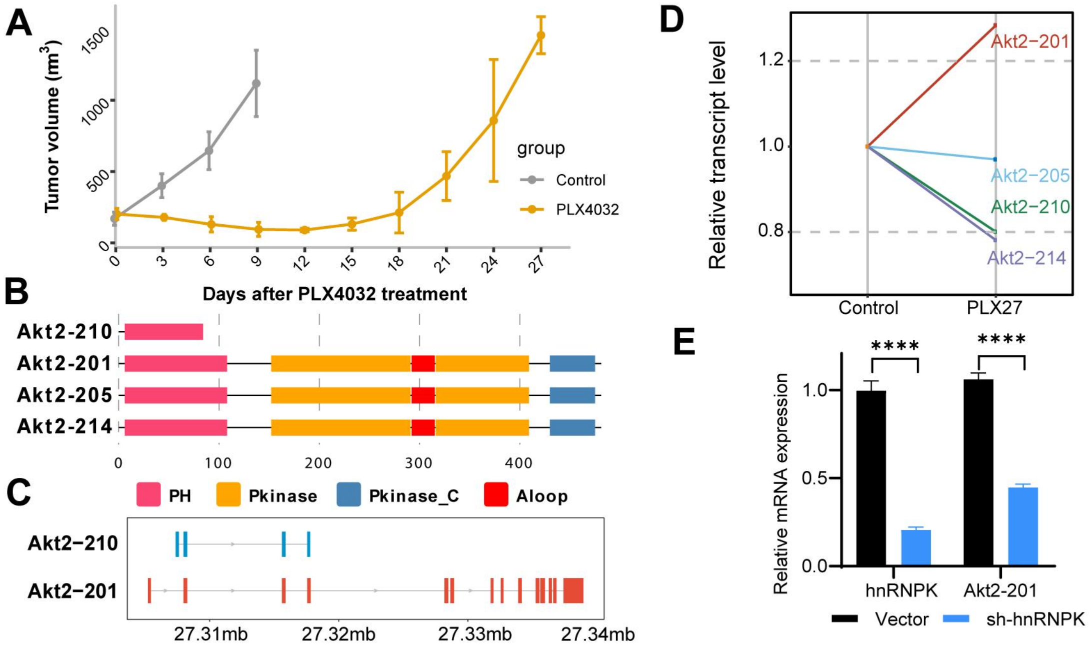


**Appendix Figure S15. The splicing alteration of Akt2 during the evolution of MAPKi resistance in mouse model.** (A) Growth curves of SMM102 tumors treated with vehicle or PLX4032 for the indicated days (mean±SD, n=4, biological replicates). (B) The functional domains of different Akt2 splicing isoforms. (C) Exon structure of Akt2-210 and Akt2-201 splicing isoforms. (D) Expression profiling of Akt2 isoforms in mouse model treated with PLX4032. (E) The expression levels of hnRNPK and Akt2-201 after knockdown of hnRNPK. The data are shown as mean±SD (n=3, biological replicates); ANOVA. **** $p < 0.0001$.

## Appendix Figure S16

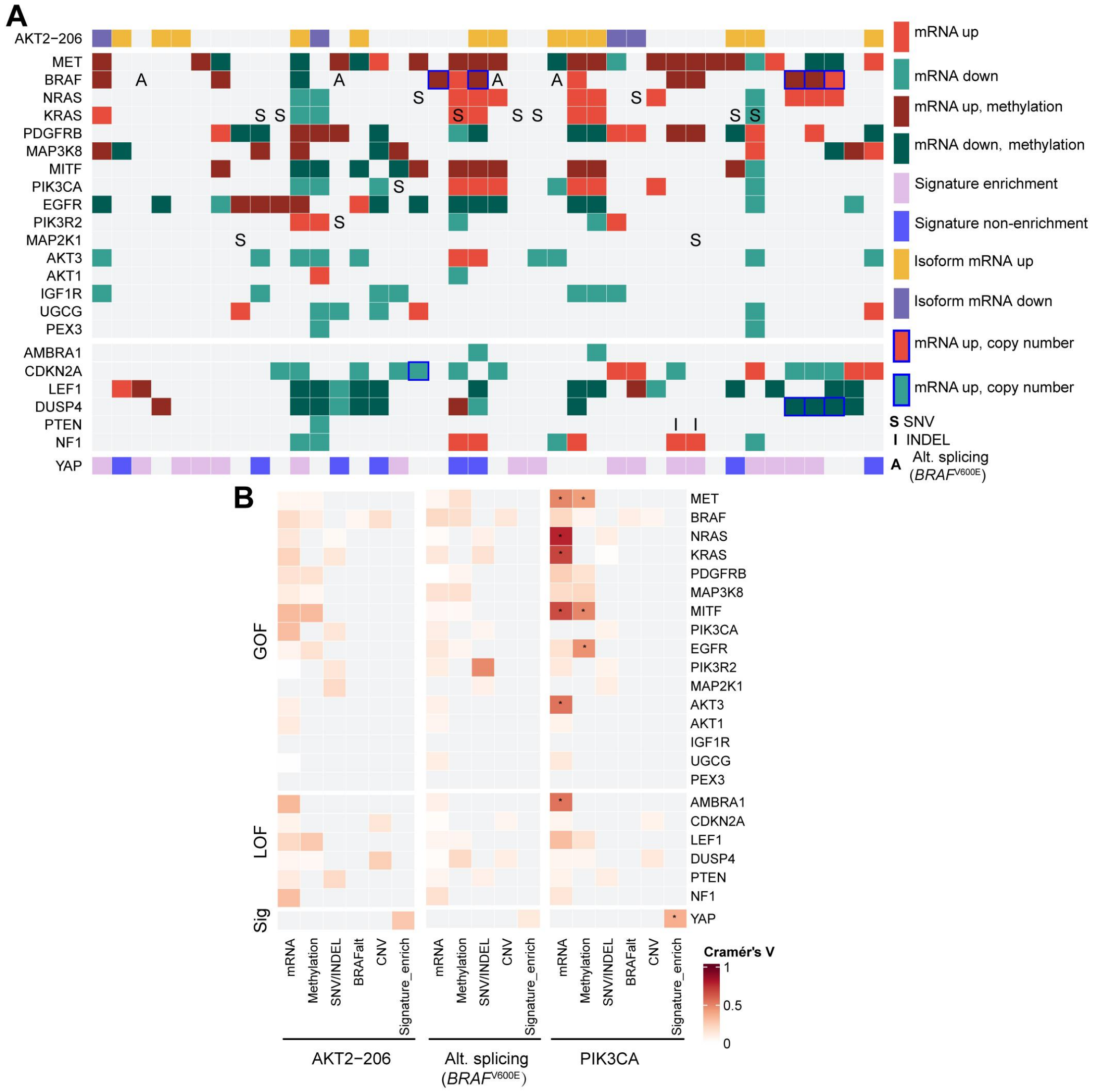


**Appendix Figure S16. Independence of AKT2 splicing switch from known resistance mechanisms.** (A) Recurrence of alterations and heterogeneity mechanistic of known resistance genes and AKT2-206 in acquired resistant tumors. (B) Correlation analysis of AKT2-206, *BRAF*$^{V600E}$ alternative splicing, PIK3CA and identified drug resistance drivers. Fisher's exact test and Cramer's V correlation. * $p < 0.05$.

**Appendix Figure S17**

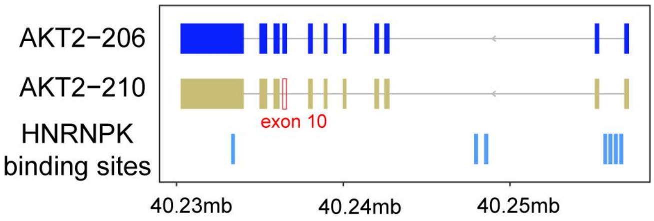


**Appendix Figure S17. Location of the hnRNPK binding site relative to AKT2 exon 10.**
