# Supplementary figures and images for: Transcriptome-wide decoding the roles of aberrant splicing in melanoma MAPK-targeted resistance evolution
Source: EMBO Rep. 2025 Jul 18;26(16):4180–96. doi: 10.1038/s44319-025-00521-6 (PMC12373858; doi:10.1038/s44319-025-00521-6)

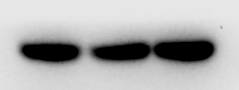

Supplement: Supplementary file 14 — Source data Fig. 5 [file 44319_2025_521_MOESM14_ESM.zip › Figure 5/5B/28-actin-1.tif]

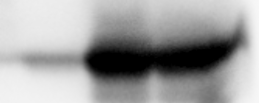

Supplement: Supplementary file 14 — Source data Fig. 5 [file 44319_2025_521_MOESM14_ESM.zip › Figure 5/5B/28-AKT-1.tif]

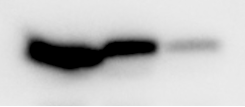

Supplement: Supplementary file 14 — Source data Fig. 5 [file 44319_2025_521_MOESM14_ESM.zip › Figure 5/5B/28-hnrnpk-1.tif]

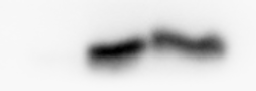

Supplement: Supplementary file 14 — Source data Fig. 5 [file 44319_2025_521_MOESM14_ESM.zip › Figure 5/5B/28-pAKT-1.tif]

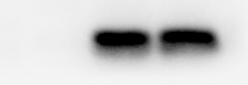

Supplement: Supplementary file 14 — Source data Fig. 5 [file 44319_2025_521_MOESM14_ESM.zip › Figure 5/5B/28-pAKT2-1.tif]

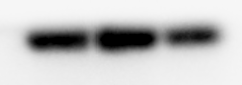

Supplement: Supplementary file 14 — Source data Fig. 5 [file 44319_2025_521_MOESM14_ESM.zip › Figure 5/5B/28-pS6-1.tif]

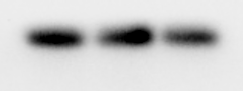

Supplement: Supplementary file 14 — Source data Fig. 5 [file 44319_2025_521_MOESM14_ESM.zip › Figure 5/5B/28-s6-1.tif]

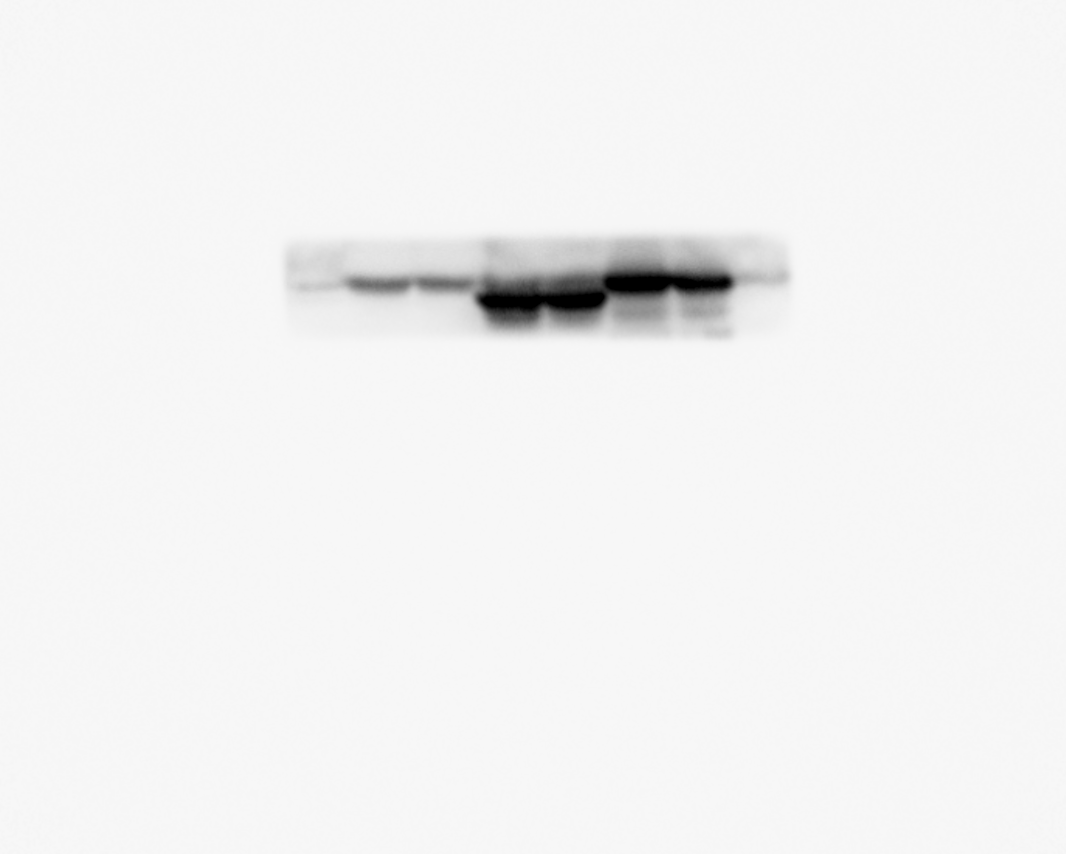

Supplement: Supplementary file 14 — Source data Fig. 5 [file 44319_2025_521_MOESM14_ESM.zip › Figure 5/5B/375-AKT-2.tif]

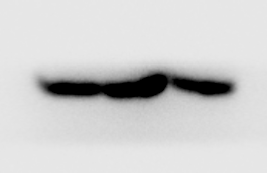

Supplement: Supplementary file 14 — Source data Fig. 5 [file 44319_2025_521_MOESM14_ESM.zip › Figure 5/5B/A375-actin-1.tif]

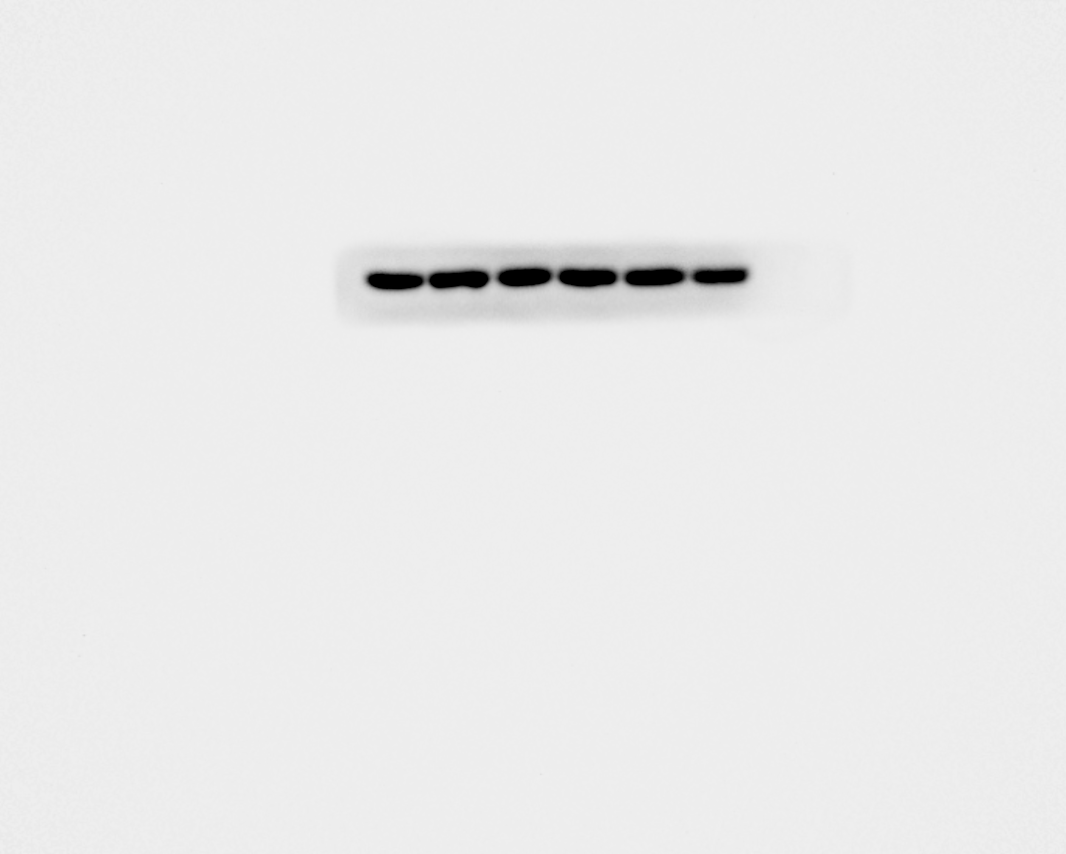

Supplement: Supplementary file 14 — Source data Fig. 5 [file 44319_2025_521_MOESM14_ESM.zip › Figure 5/5B/A375-actin-2.tif]

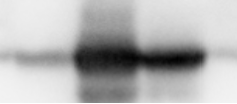

Supplement: Supplementary file 14 — Source data Fig. 5 [file 44319_2025_521_MOESM14_ESM.zip › Figure 5/5B/A375-AKT-1.tif]

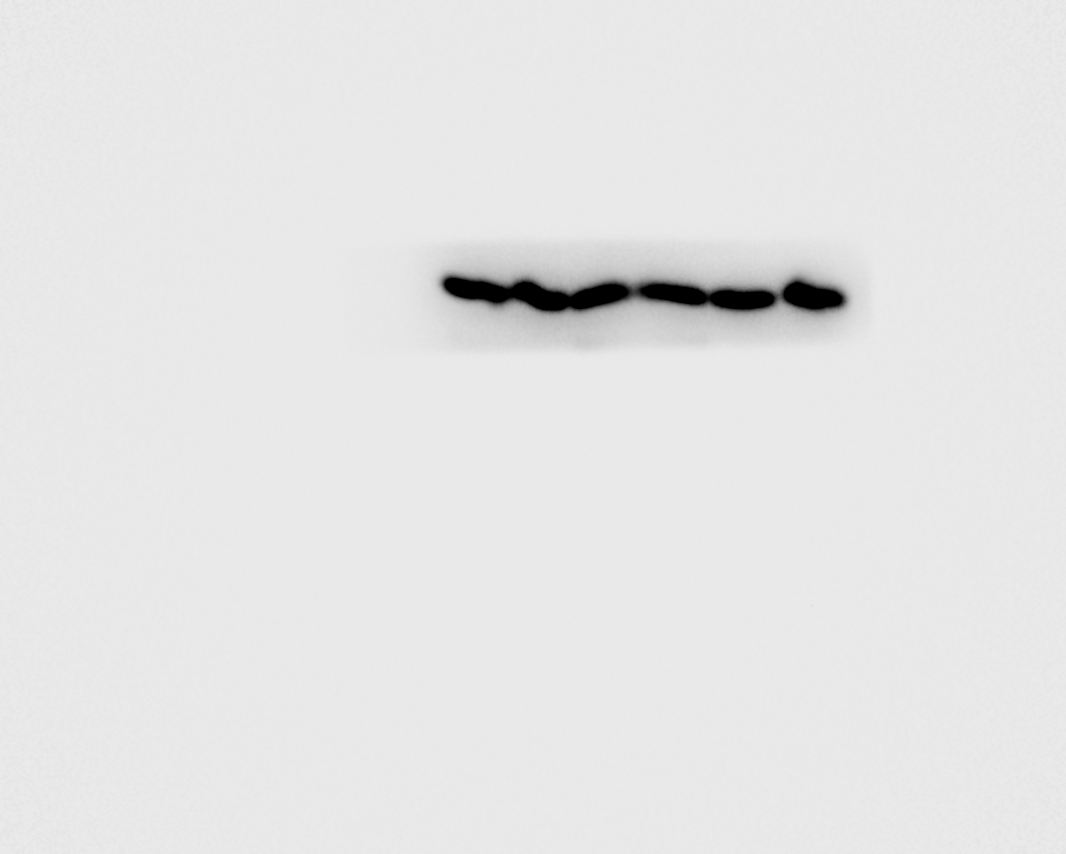

Supplement: Supplementary file 14 — Source data Fig. 5 [file 44319_2025_521_MOESM14_ESM.zip › Figure 5/5B/A375-bax-2.tif]

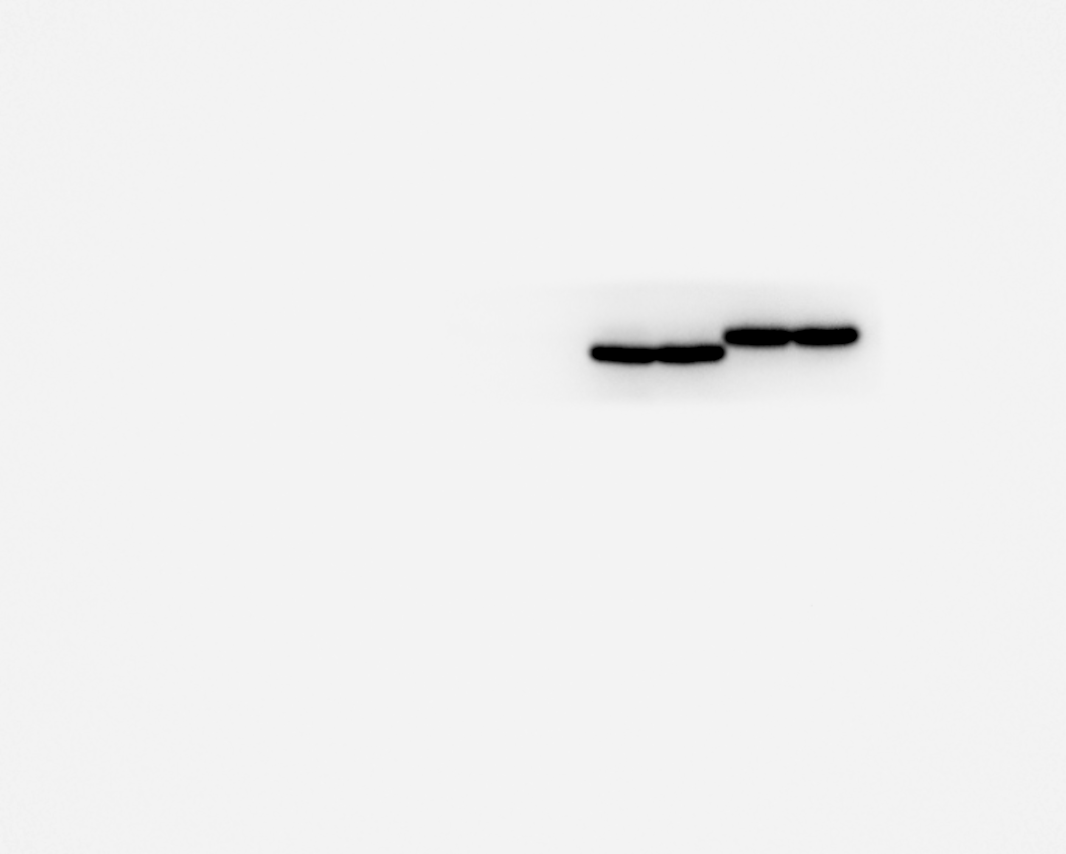

Supplement: Supplementary file 14 — Source data Fig. 5 [file 44319_2025_521_MOESM14_ESM.zip › Figure 5/5B/A375-flag-2.tif]

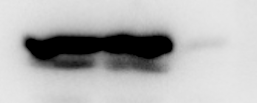

Supplement: Supplementary file 14 — Source data Fig. 5 [file 44319_2025_521_MOESM14_ESM.zip › Figure 5/5B/A375-hnrnpk-1.tif]

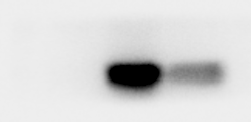

Supplement: Supplementary file 14 — Source data Fig. 5 [file 44319_2025_521_MOESM14_ESM.zip › Figure 5/5B/A375-pAKT-1.tif]

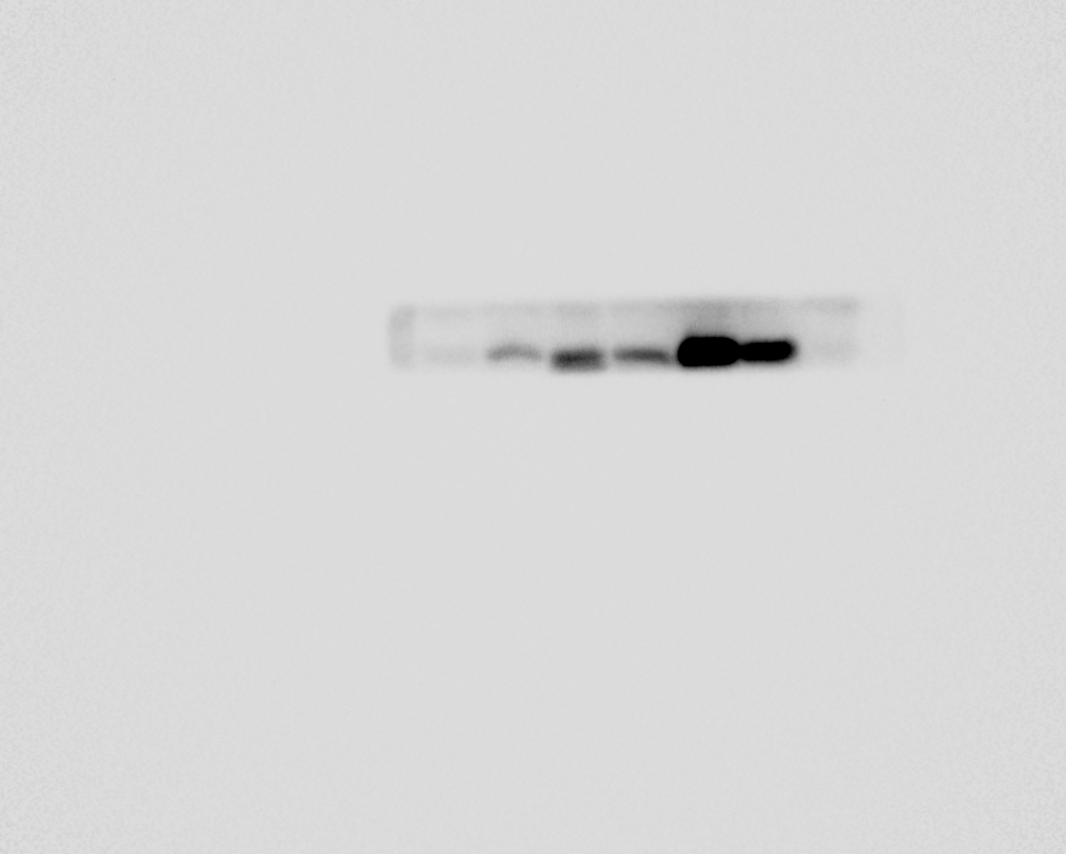

Supplement: Supplementary file 14 — Source data Fig. 5 [file 44319_2025_521_MOESM14_ESM.zip › Figure 5/5B/A375-pAKT-2.tif]

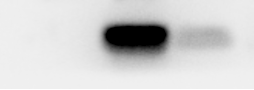

Supplement: Supplementary file 14 — Source data Fig. 5 [file 44319_2025_521_MOESM14_ESM.zip › Figure 5/5B/A375-pAKT2-1.tif]

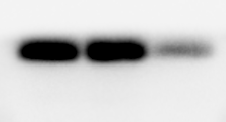

Supplement: Supplementary file 14 — Source data Fig. 5 [file 44319_2025_521_MOESM14_ESM.zip › Figure 5/5B/A375-pS6-1.tif]

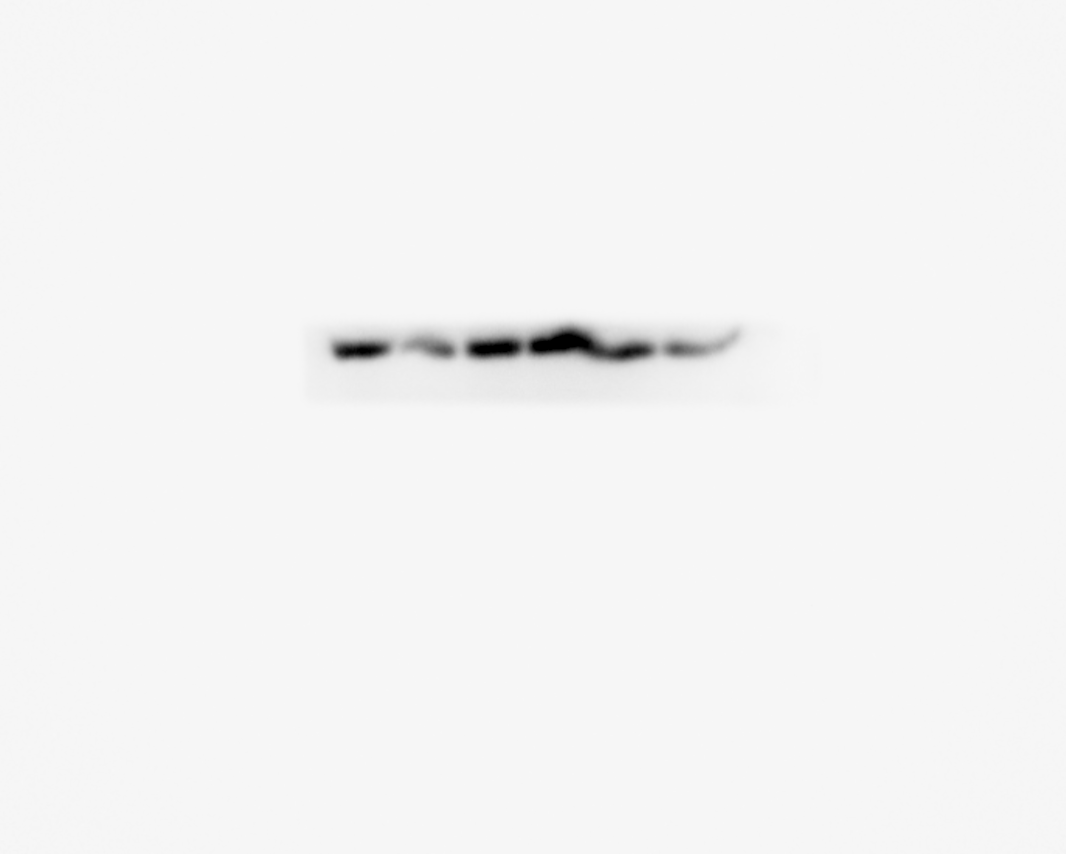

Supplement: Supplementary file 14 — Source data Fig. 5 [file 44319_2025_521_MOESM14_ESM.zip › Figure 5/5B/A375-pS6-2.tif]

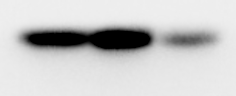

Supplement: Supplementary file 14 — Source data Fig. 5 [file 44319_2025_521_MOESM14_ESM.zip › Figure 5/5B/A375-s6-1.tif]

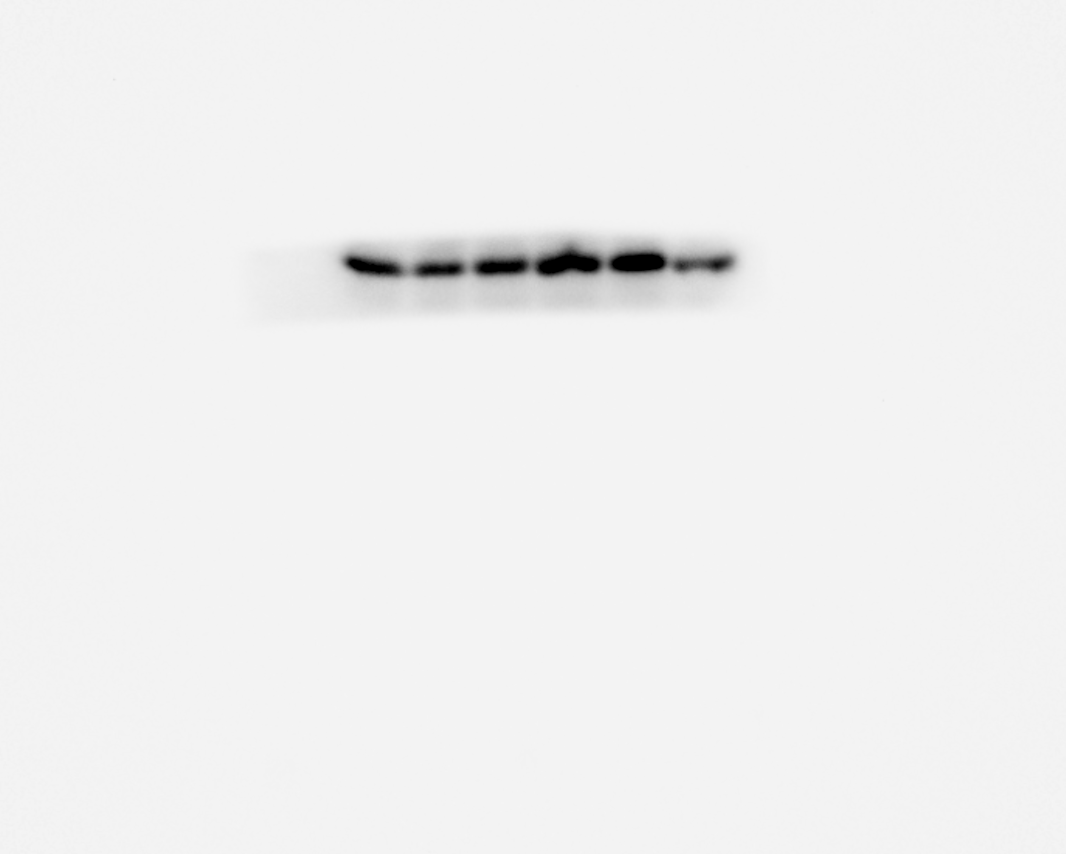

Supplement: Supplementary file 14 — Source data Fig. 5 [file 44319_2025_521_MOESM14_ESM.zip › Figure 5/5B/A375-s6-2.tif]

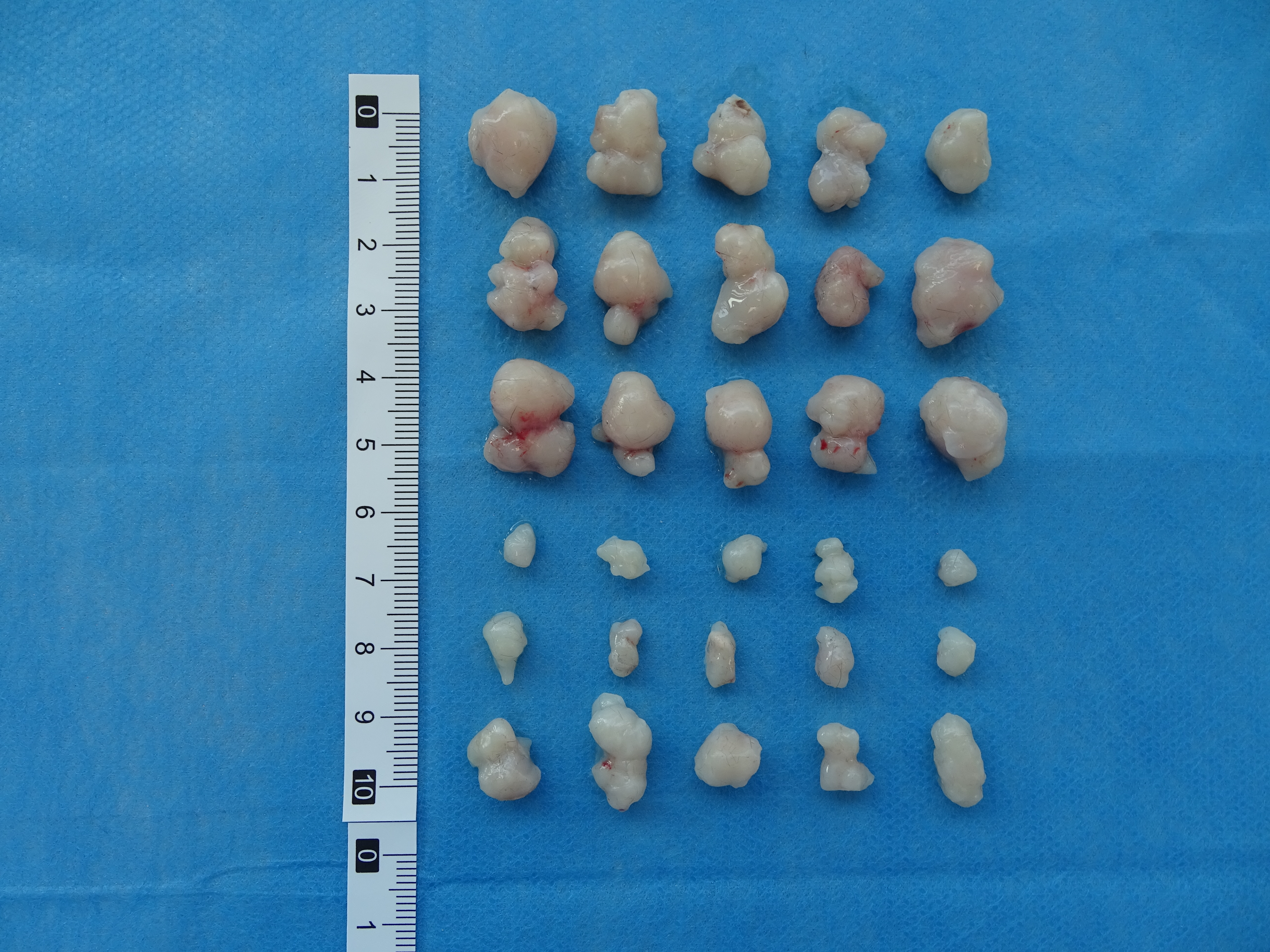

Supplement: Supplementary file 14 — Source data Fig. 5 [file 44319_2025_521_MOESM14_ESM.zip › Figure 5/5E/tumor.JPG]

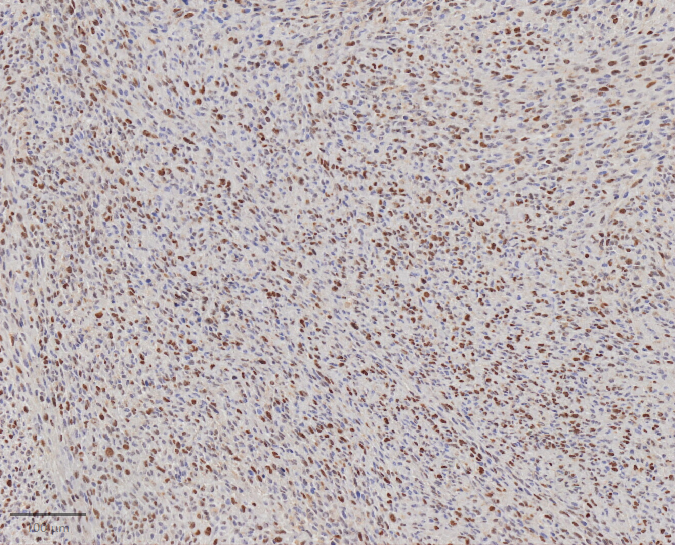

Supplement: Supplementary file 14 — Source data Fig. 5 [file 44319_2025_521_MOESM14_ESM.zip › Figure 5/5F/Ki67/AKT2-206-.png]

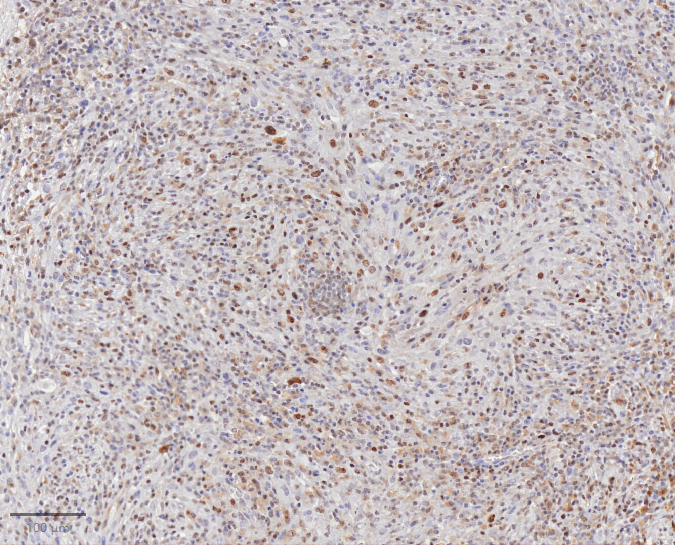

Supplement: Supplementary file 14 — Source data Fig. 5 [file 44319_2025_521_MOESM14_ESM.zip › Figure 5/5F/Ki67/AKT2-210.png]

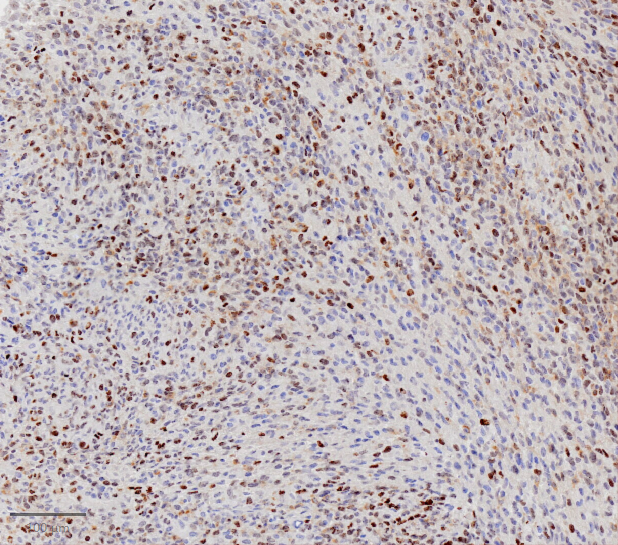

Supplement: Supplementary file 14 — Source data Fig. 5 [file 44319_2025_521_MOESM14_ESM.zip › Figure 5/5F/Ki67/Control.png]

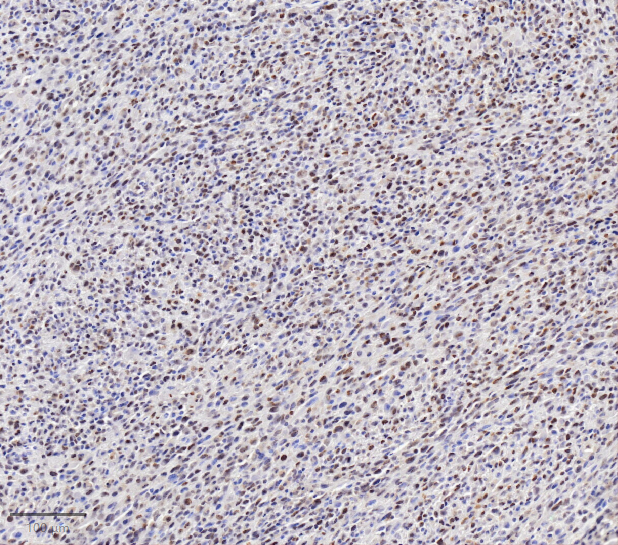

Supplement: Supplementary file 14 — Source data Fig. 5 [file 44319_2025_521_MOESM14_ESM.zip › Figure 5/5F/Ki67/PLX+AKT2-206.png]

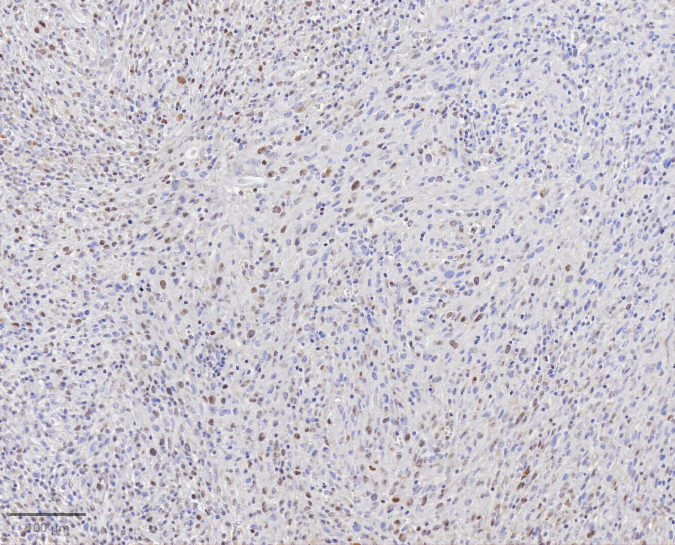

Supplement: Supplementary file 14 — Source data Fig. 5 [file 44319_2025_521_MOESM14_ESM.zip › Figure 5/5F/Ki67/PLX+AKT2-210 Ki67.png]

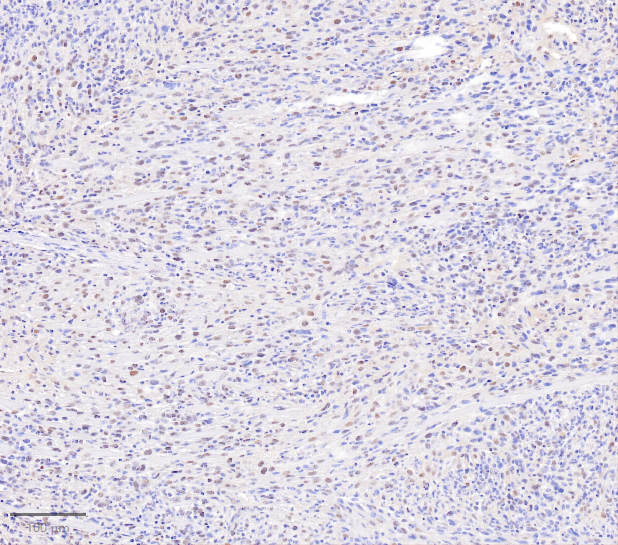

Supplement: Supplementary file 14 — Source data Fig. 5 [file 44319_2025_521_MOESM14_ESM.zip › Figure 5/5F/Ki67/PLX.png]

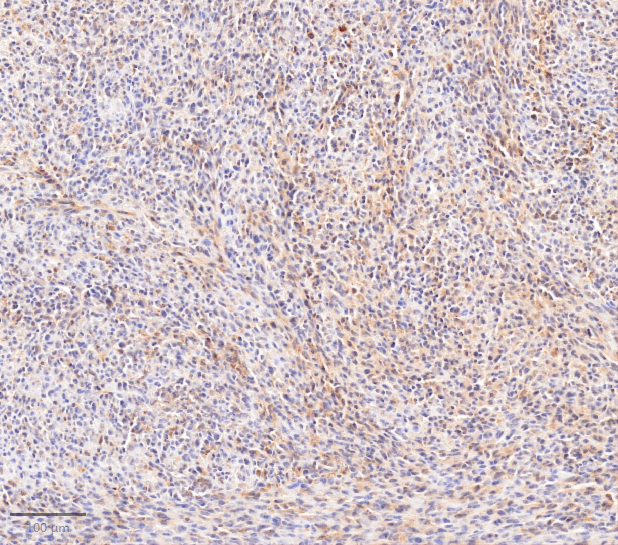

Supplement: Supplementary file 14 — Source data Fig. 5 [file 44319_2025_521_MOESM14_ESM.zip › Figure 5/5F/p-AKT S473/AKT2-206.png]

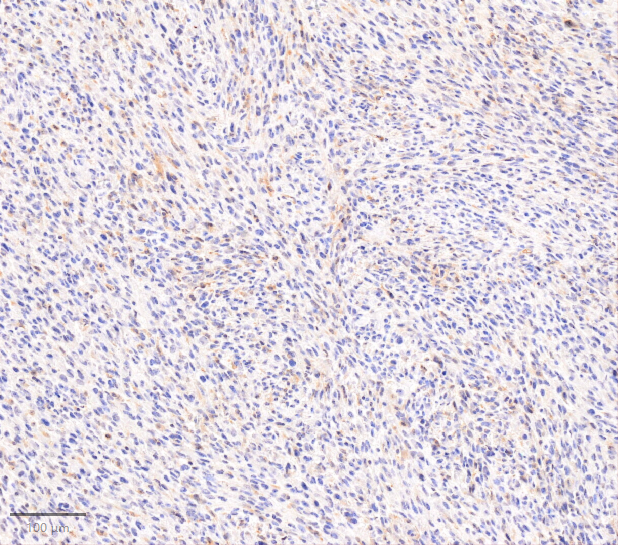

Supplement: Supplementary file 14 — Source data Fig. 5 [file 44319_2025_521_MOESM14_ESM.zip › Figure 5/5F/p-AKT S473/AKT2-210.png]

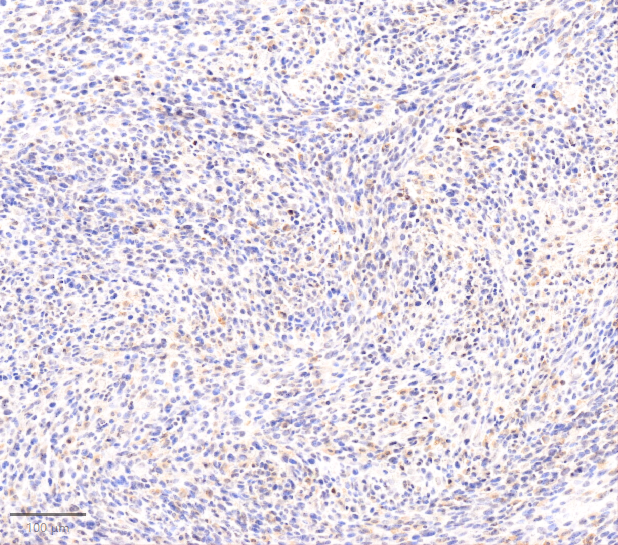

Supplement: Supplementary file 14 — Source data Fig. 5 [file 44319_2025_521_MOESM14_ESM.zip › Figure 5/5F/p-AKT S473/Control.png]

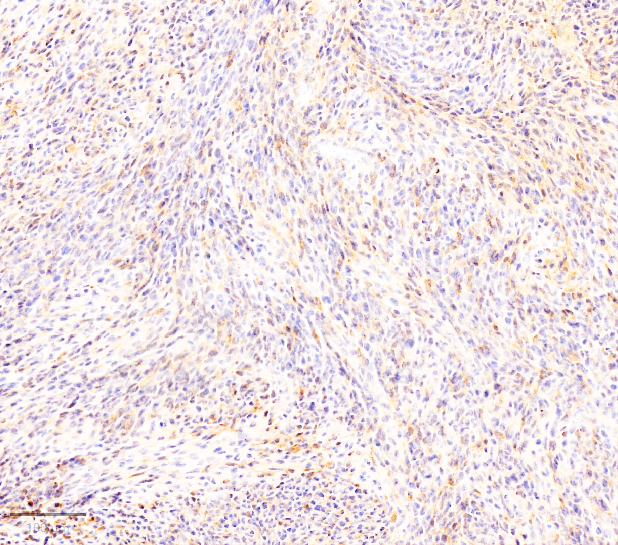

Supplement: Supplementary file 14 — Source data Fig. 5 [file 44319_2025_521_MOESM14_ESM.zip › Figure 5/5F/p-AKT S473/PLX+AKT2-206.png]

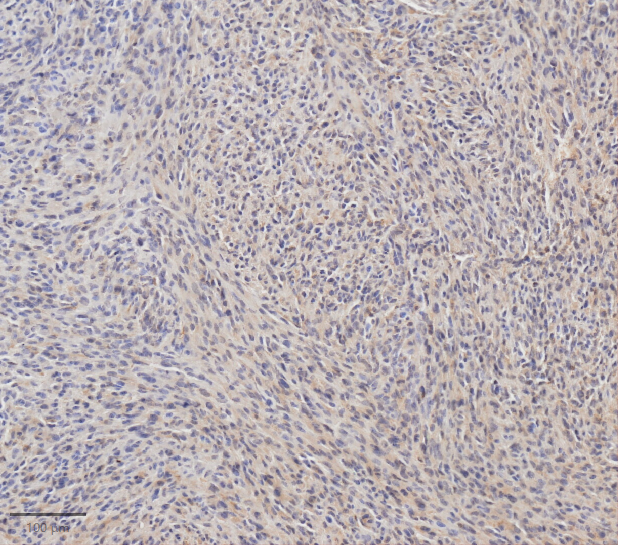

Supplement: Supplementary file 14 — Source data Fig. 5 [file 44319_2025_521_MOESM14_ESM.zip › Figure 5/5F/p-AKT S473/PLX+AKT2-210.png]

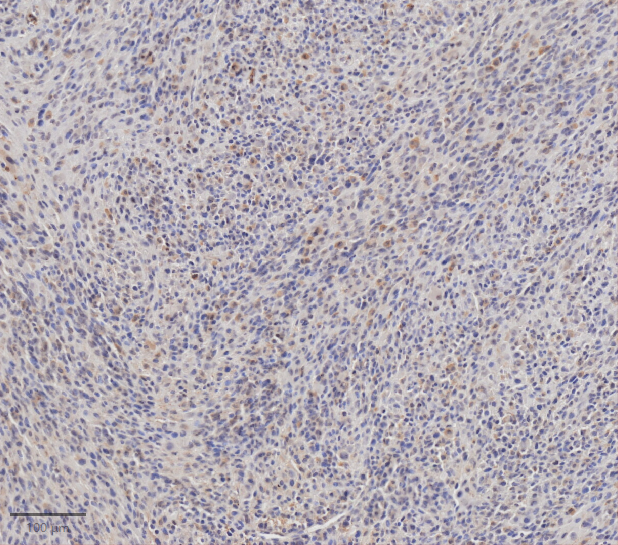

Supplement: Supplementary file 14 — Source data Fig. 5 [file 44319_2025_521_MOESM14_ESM.zip › Figure 5/5F/p-AKT S473/PLX.png]

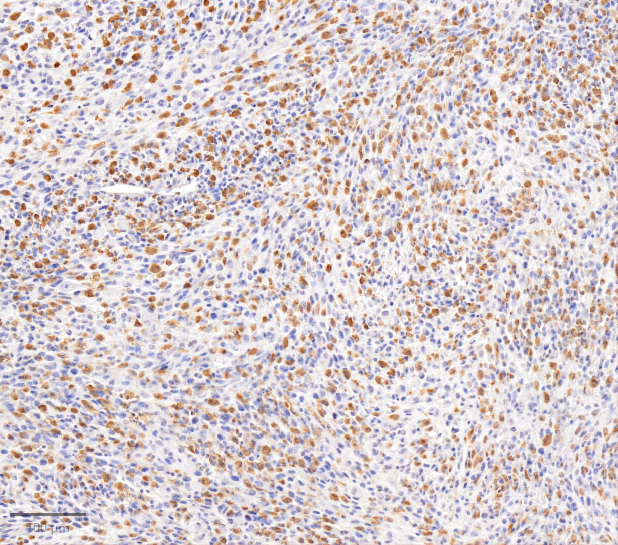

Supplement: Supplementary file 14 — Source data Fig. 5 [file 44319_2025_521_MOESM14_ESM.zip › Figure 5/5F/P-S6K/AKT2-206.png]

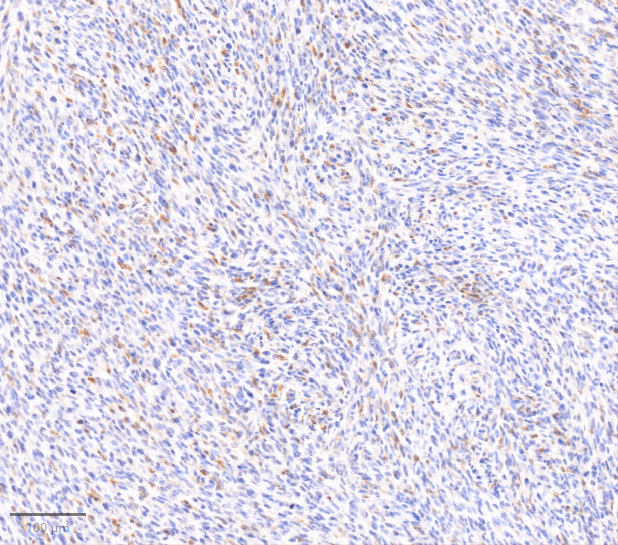

Supplement: Supplementary file 14 — Source data Fig. 5 [file 44319_2025_521_MOESM14_ESM.zip › Figure 5/5F/P-S6K/AKT2-210.png]

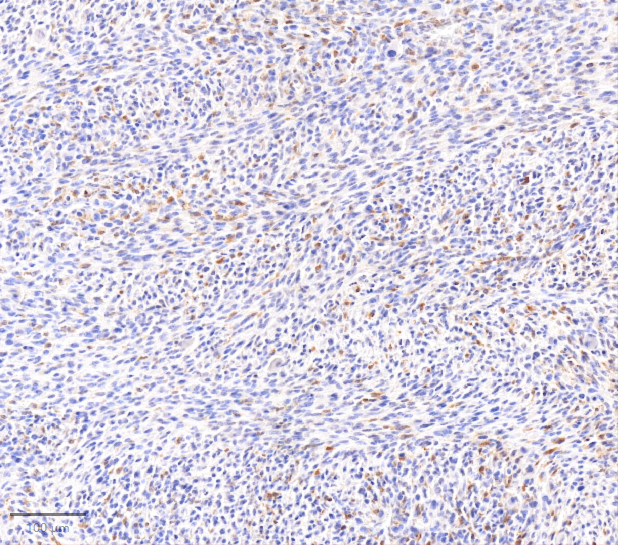

Supplement: Supplementary file 14 — Source data Fig. 5 [file 44319_2025_521_MOESM14_ESM.zip › Figure 5/5F/P-S6K/Control.png]

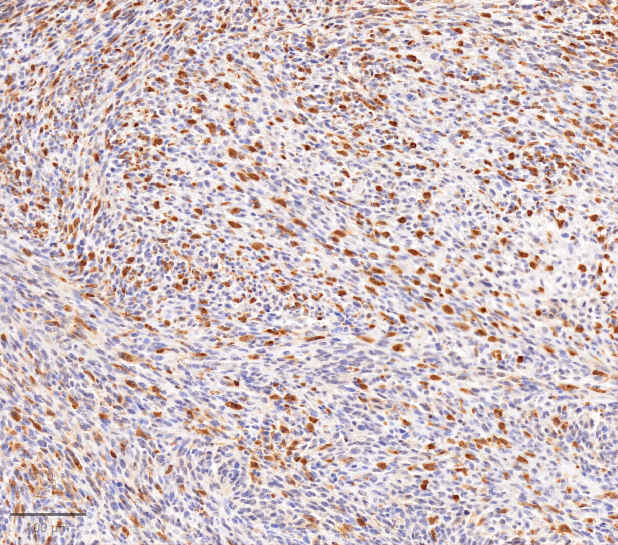

Supplement: Supplementary file 14 — Source data Fig. 5 [file 44319_2025_521_MOESM14_ESM.zip › Figure 5/5F/P-S6K/PLX+AKT2-206.png]

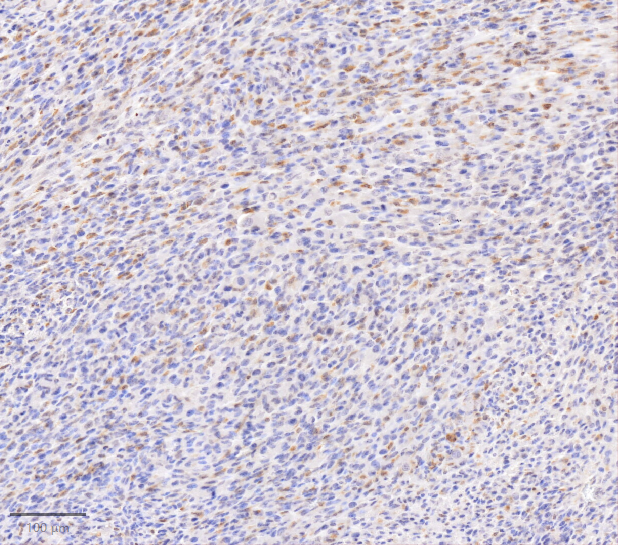

Supplement: Supplementary file 14 — Source data Fig. 5 [file 44319_2025_521_MOESM14_ESM.zip › Figure 5/5F/P-S6K/PLX+AKT2-210.png]

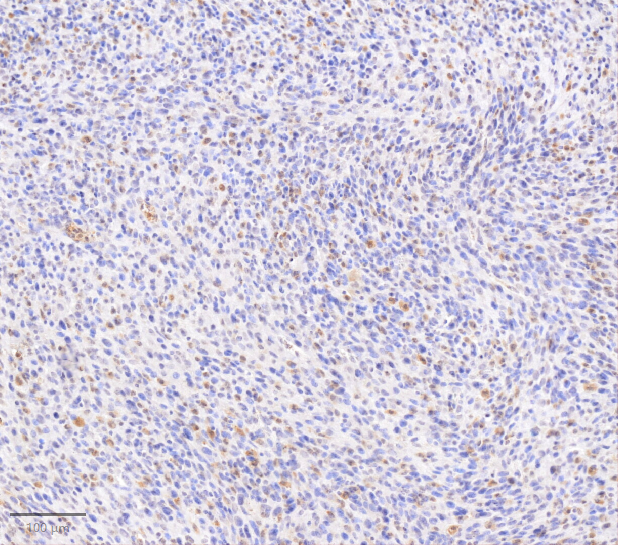

Supplement: Supplementary file 14 — Source data Fig. 5 [file 44319_2025_521_MOESM14_ESM.zip › Figure 5/5F/P-S6K/PLX.png]

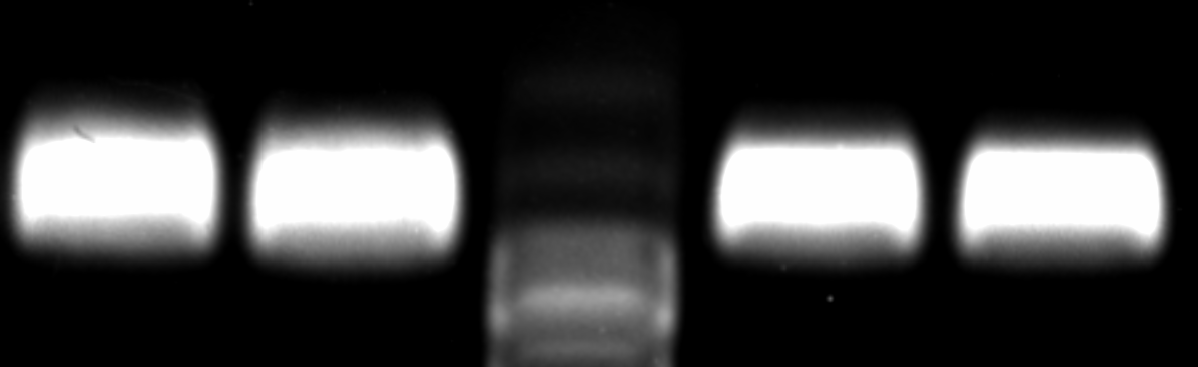

Supplement: Supplementary file 15 — Source data Fig. 6 [file 44319_2025_521_MOESM15_ESM.zip › Figure 6/6D/GAPHD.tif]

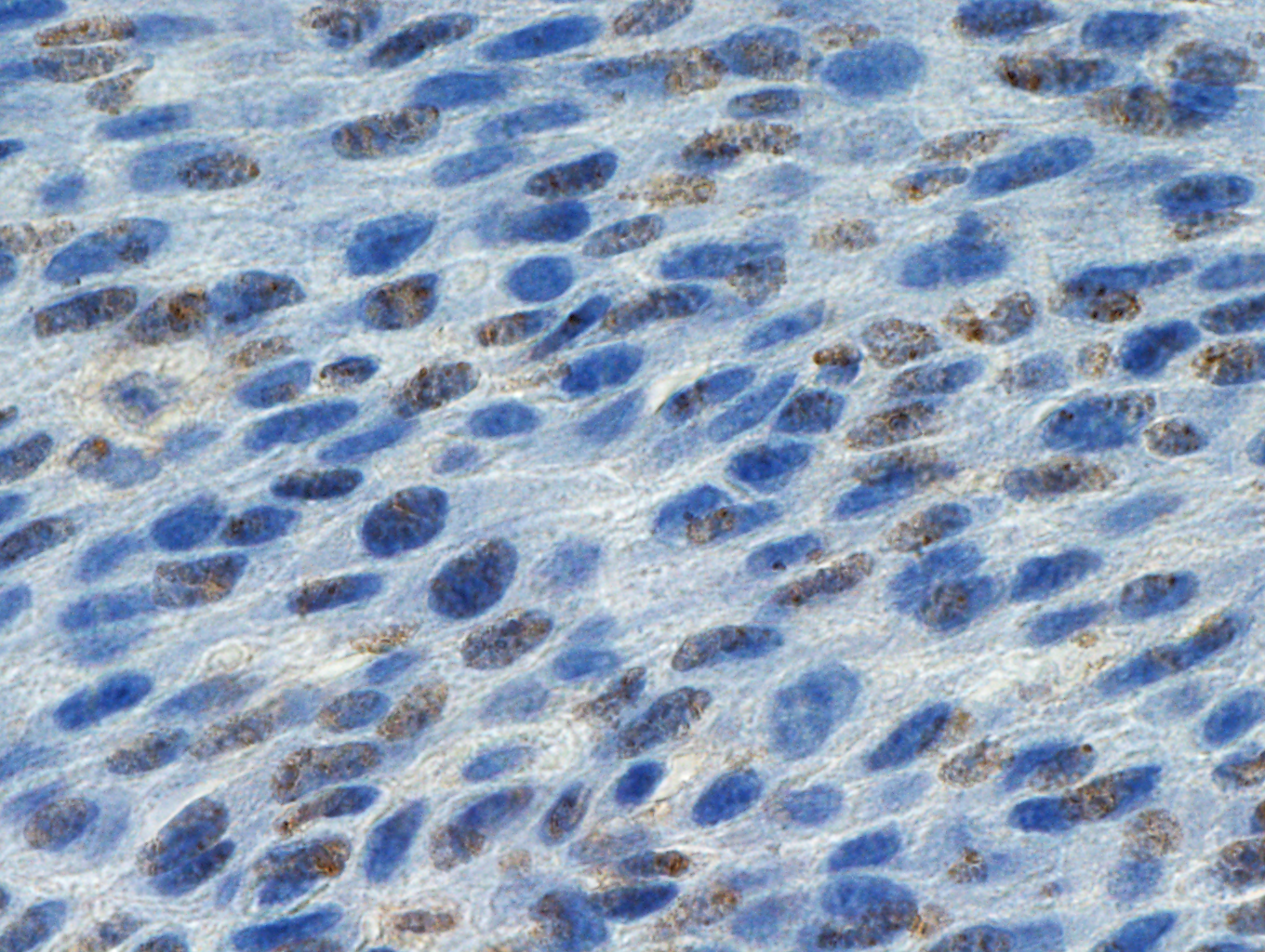

Supplement: Supplementary file 15 — Source data Fig. 6 [file 44319_2025_521_MOESM15_ESM.zip › Figure 6/6F/HNRNPK-DMSO-100C.tif]

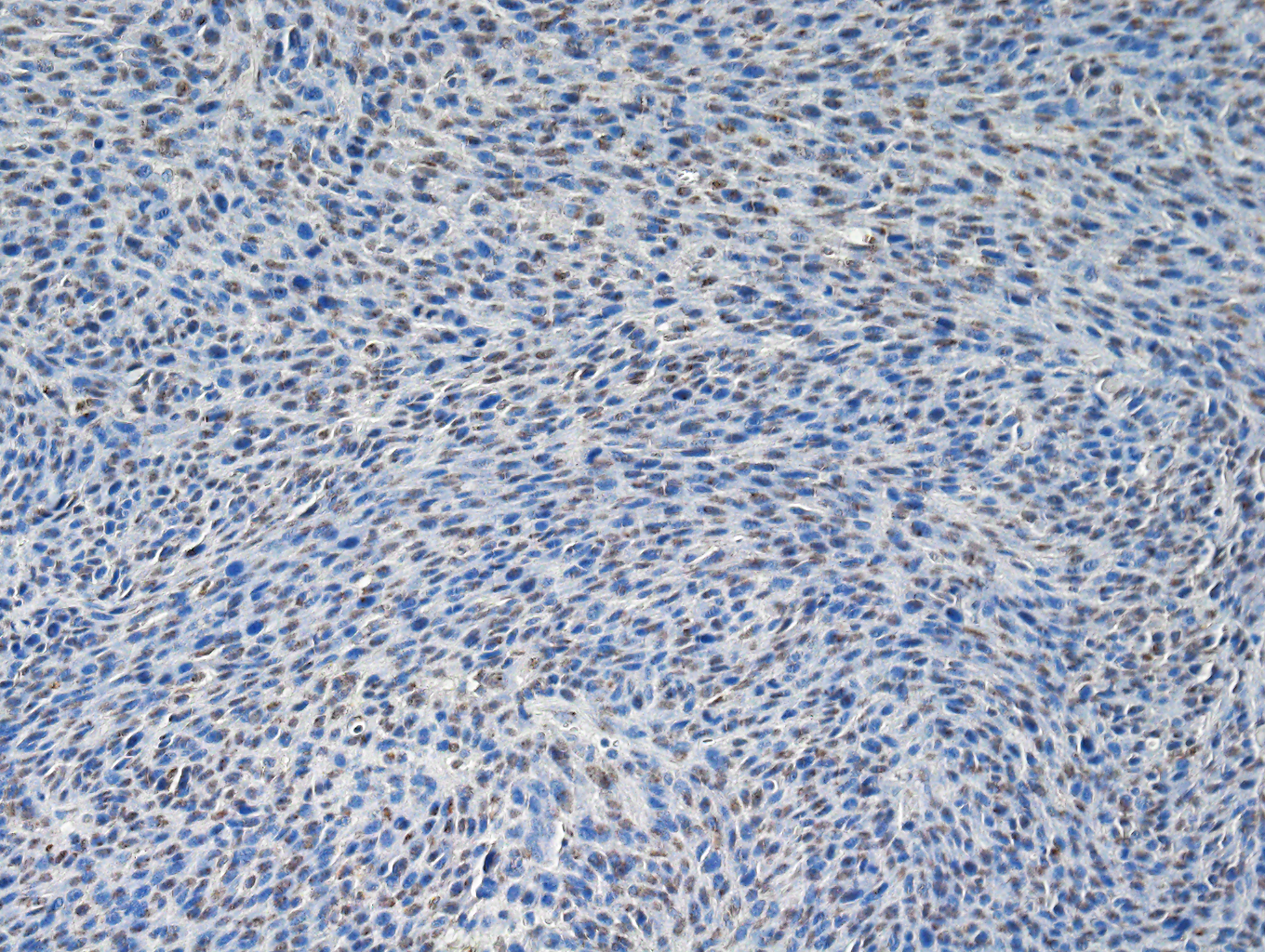

Supplement: Supplementary file 15 — Source data Fig. 6 [file 44319_2025_521_MOESM15_ESM.zip › Figure 6/6F/HNRNPK-DMSO.tif]

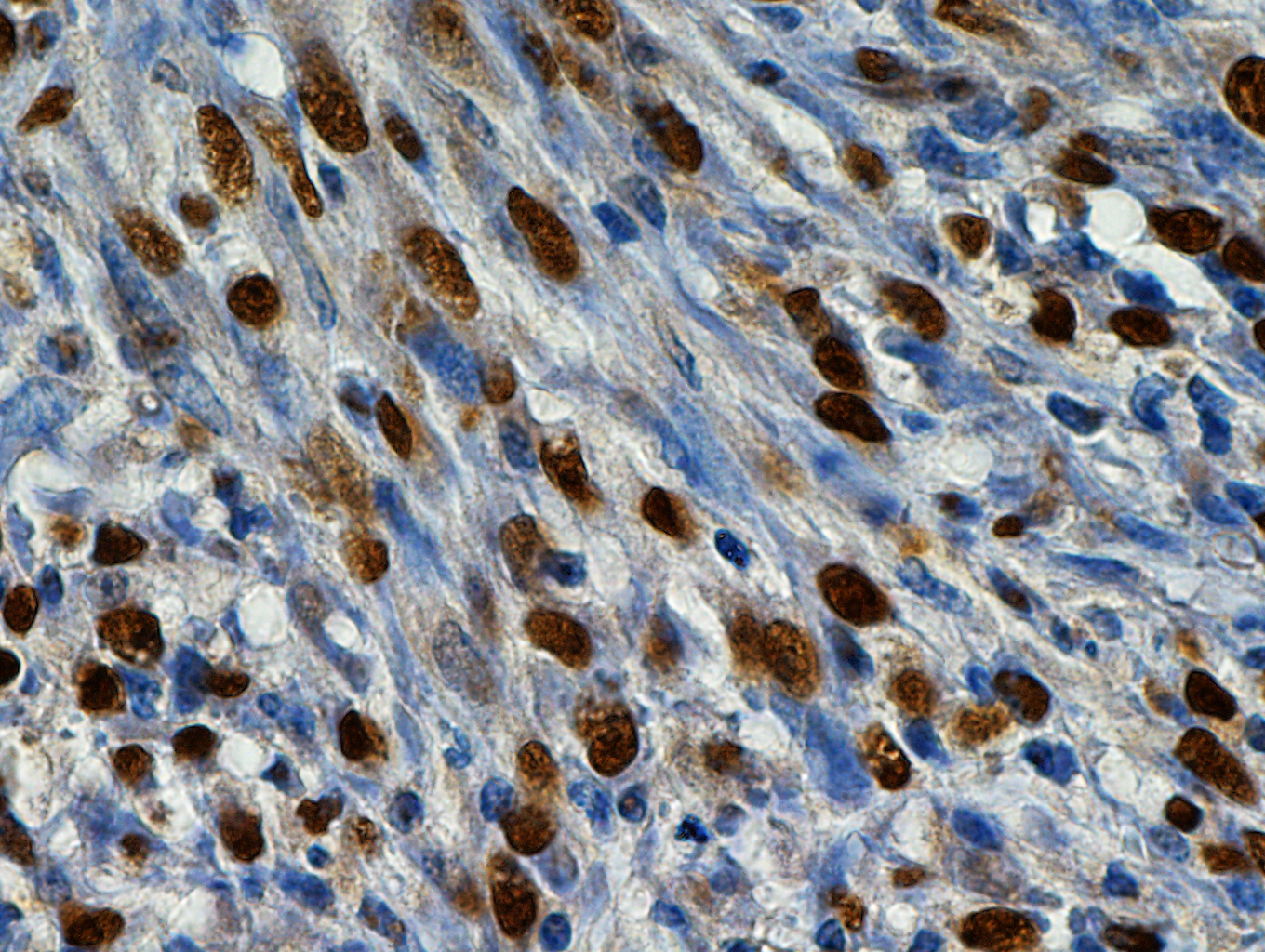

Supplement: Supplementary file 15 — Source data Fig. 6 [file 44319_2025_521_MOESM15_ESM.zip › Figure 6/6F/HNRNPK-PLX4032-100C.tif]

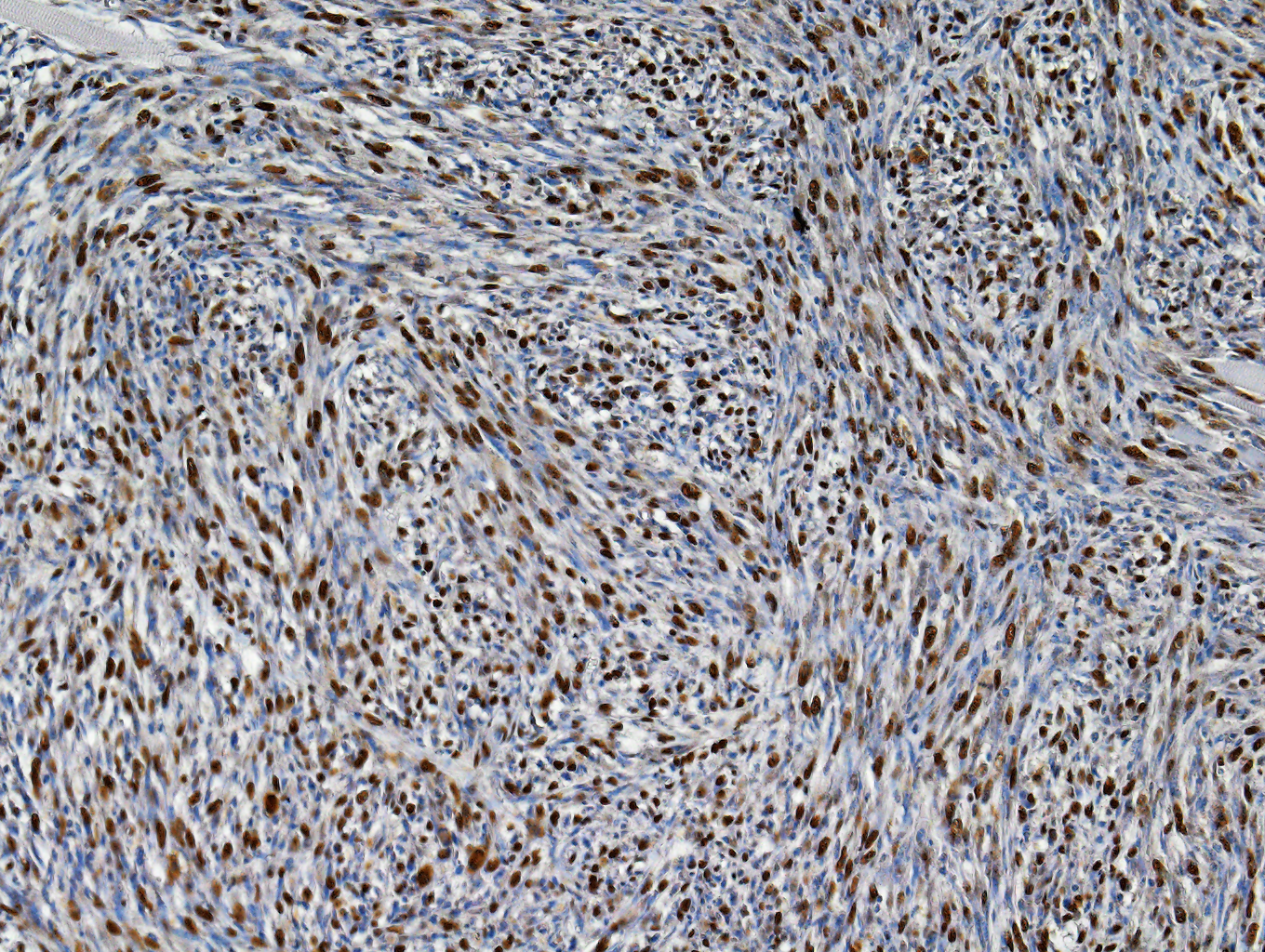

Supplement: Supplementary file 15 — Source data Fig. 6 [file 44319_2025_521_MOESM15_ESM.zip › Figure 6/6F/HNRNPK-PLX4032.tif]

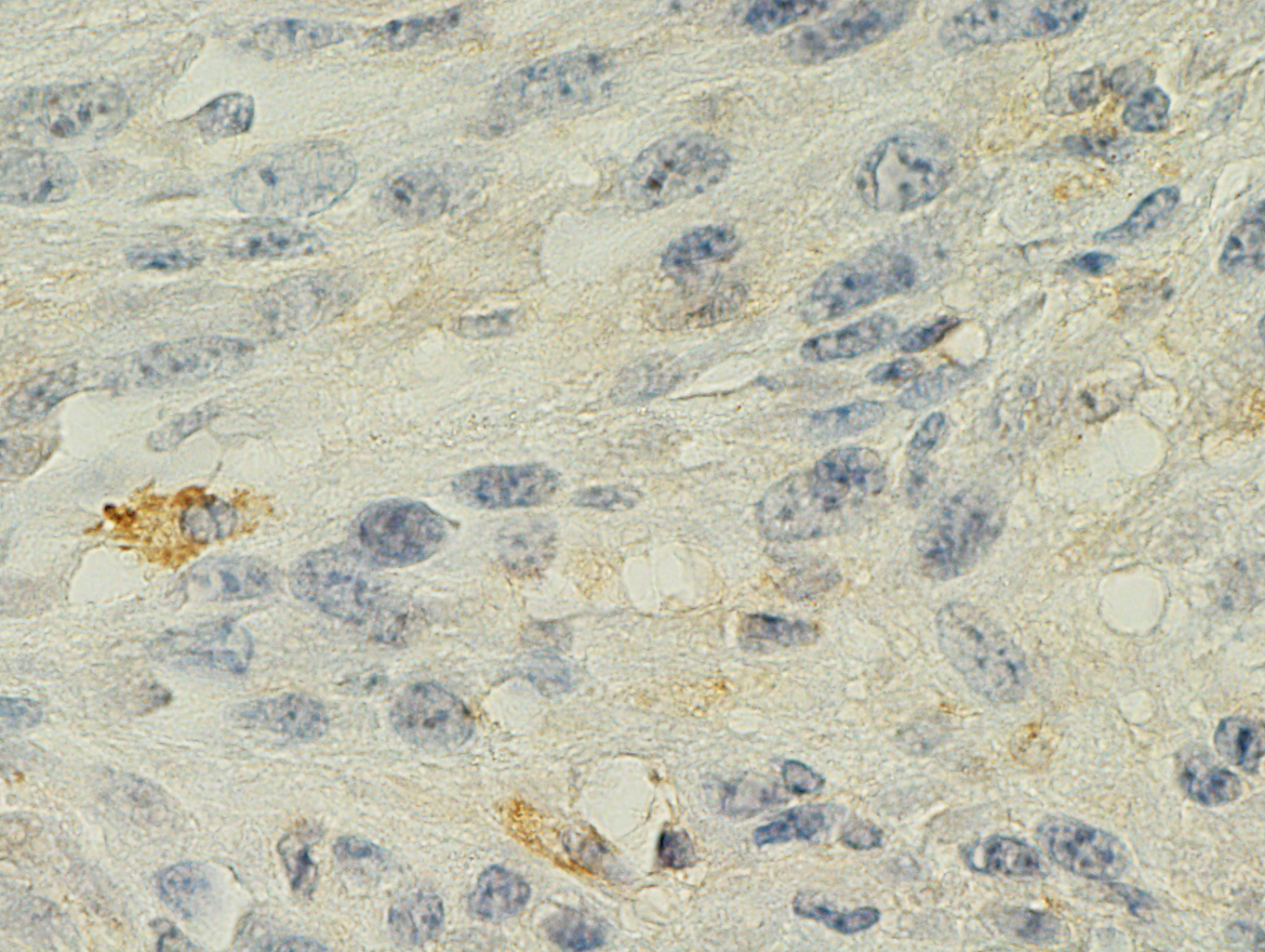

Supplement: Supplementary file 15 — Source data Fig. 6 [file 44319_2025_521_MOESM15_ESM.zip › Figure 6/6F/p-AKT DMSO-100C.tif]

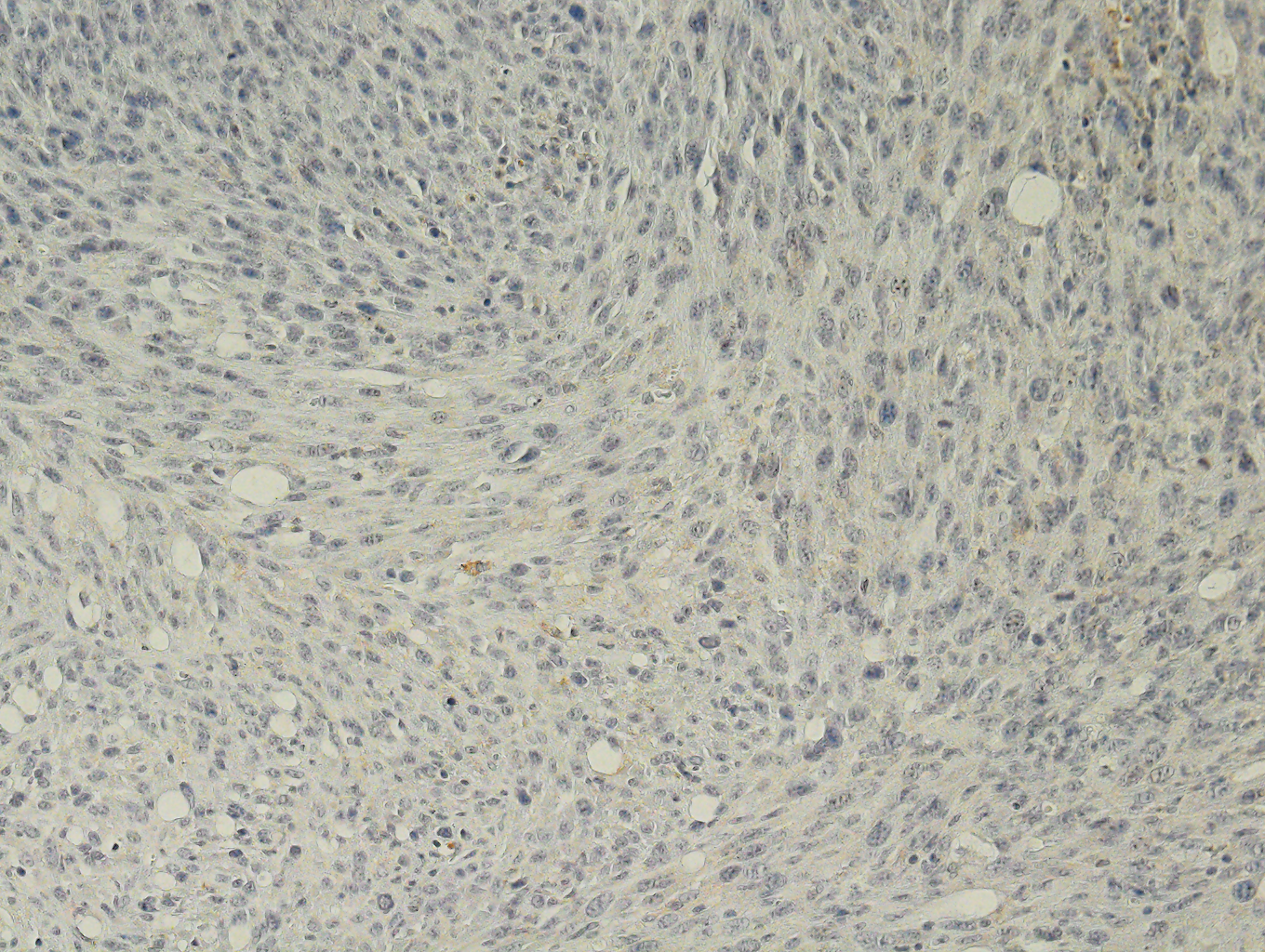

Supplement: Supplementary file 15 — Source data Fig. 6 [file 44319_2025_521_MOESM15_ESM.zip › Figure 6/6F/p-AKT DMSO.tif]

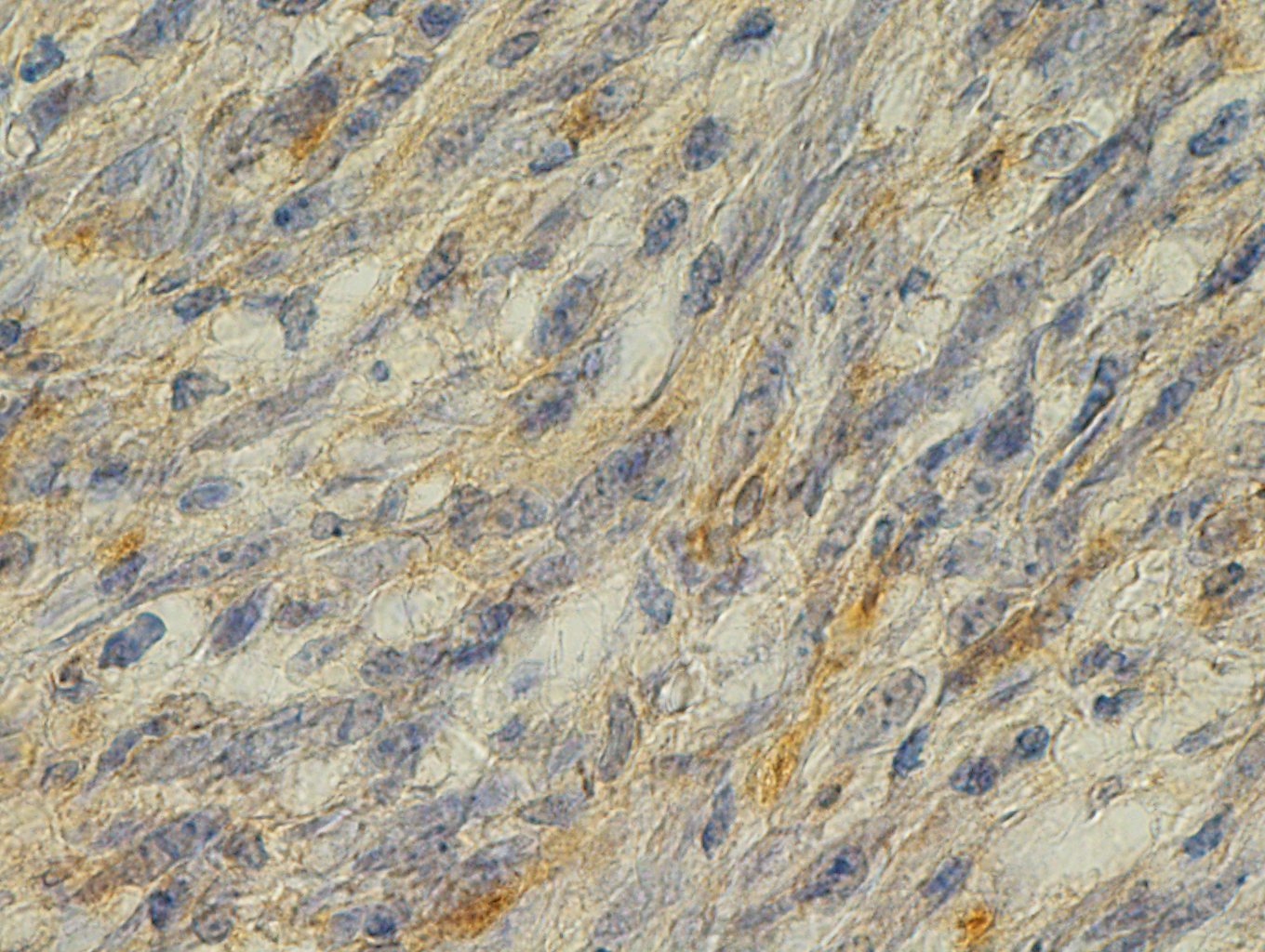

Supplement: Supplementary file 15 — Source data Fig. 6 [file 44319_2025_521_MOESM15_ESM.zip › Figure 6/6F/p-AKT PLX4032-100C.tif]

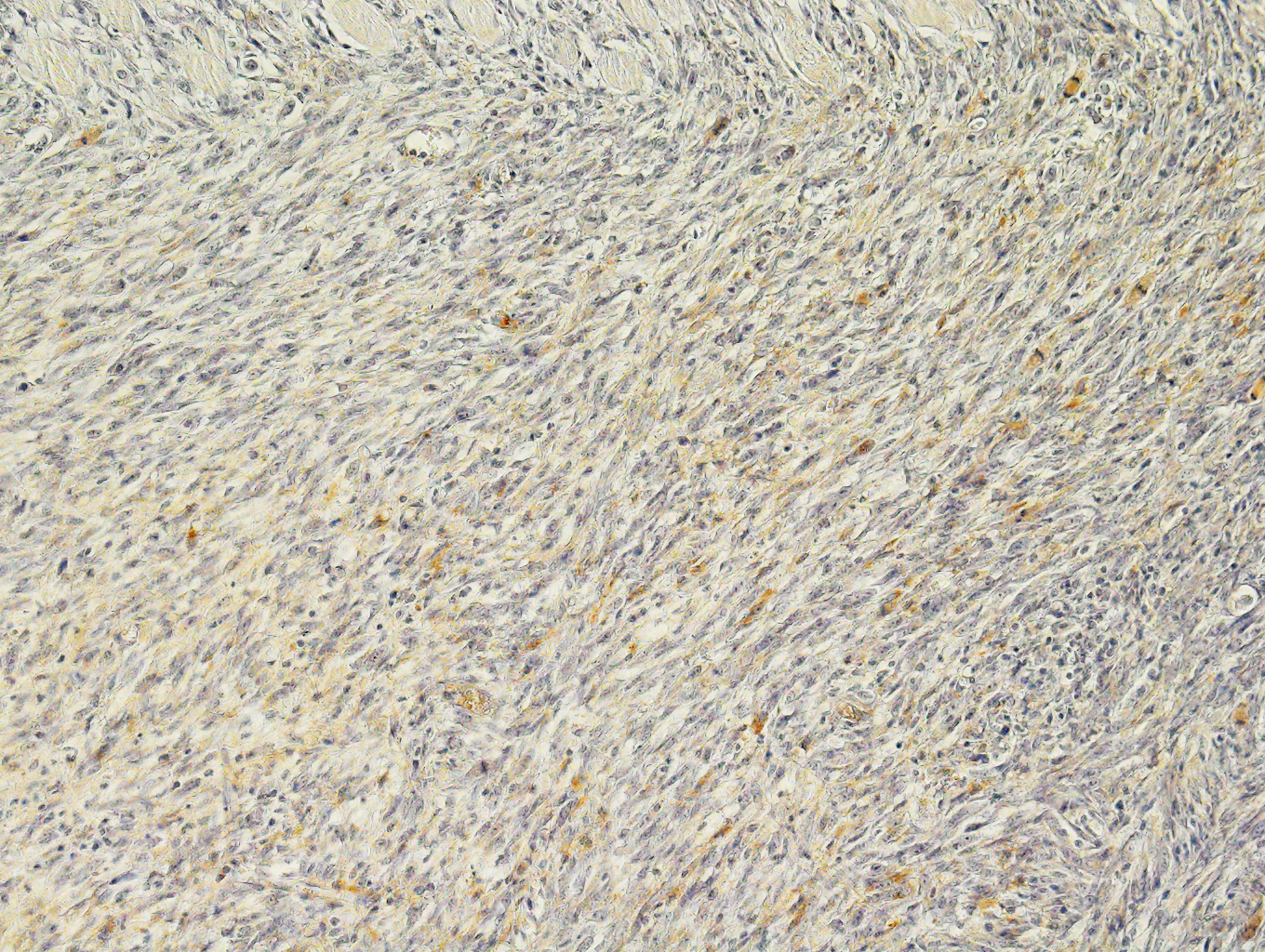

Supplement: Supplementary file 15 — Source data Fig. 6 [file 44319_2025_521_MOESM15_ESM.zip › Figure 6/6F/p-AKT PLX4032.tif]

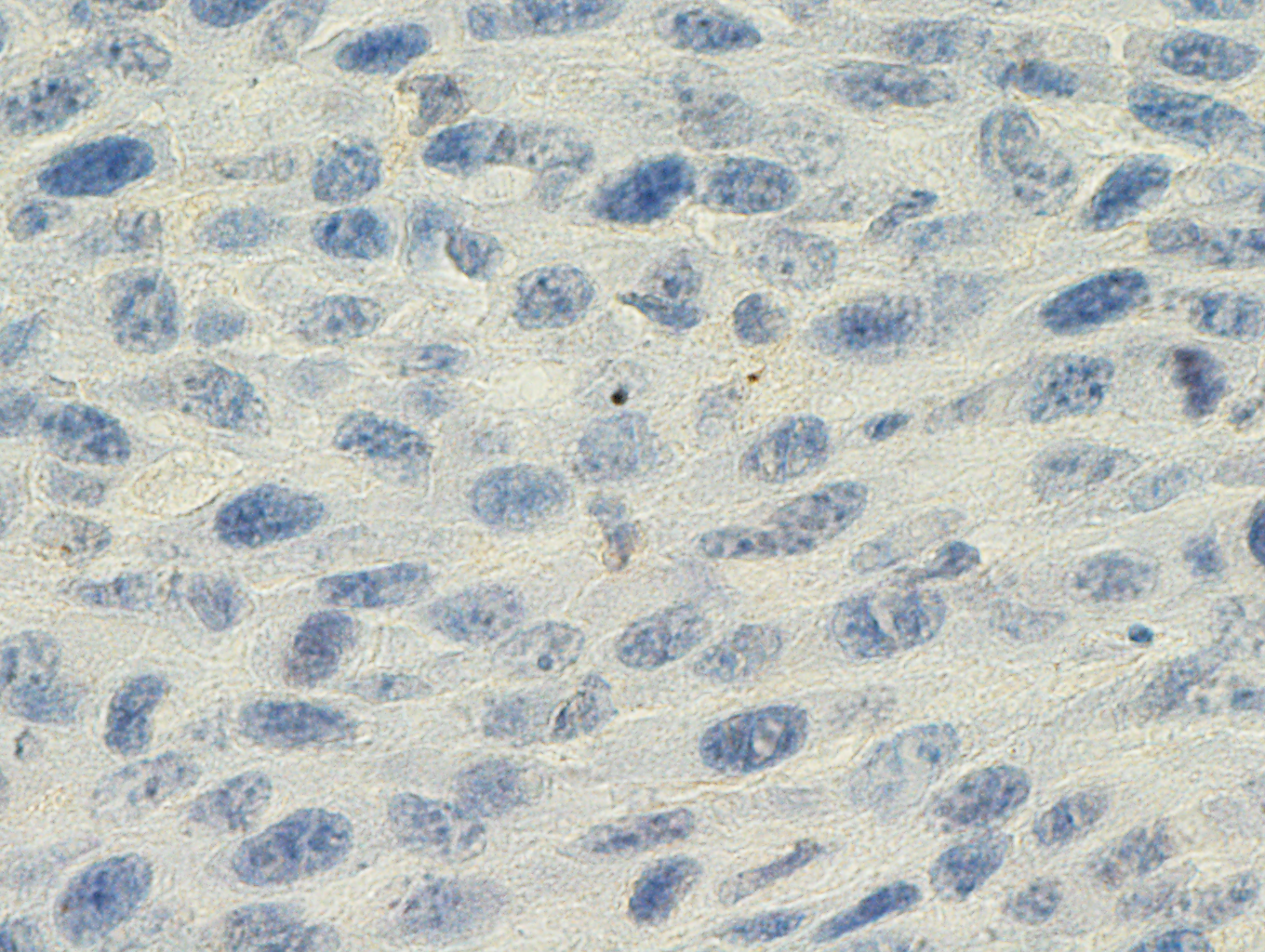

Supplement: Supplementary file 15 — Source data Fig. 6 [file 44319_2025_521_MOESM15_ESM.zip › Figure 6/6F/p-S6 DMSO-100C.tif]

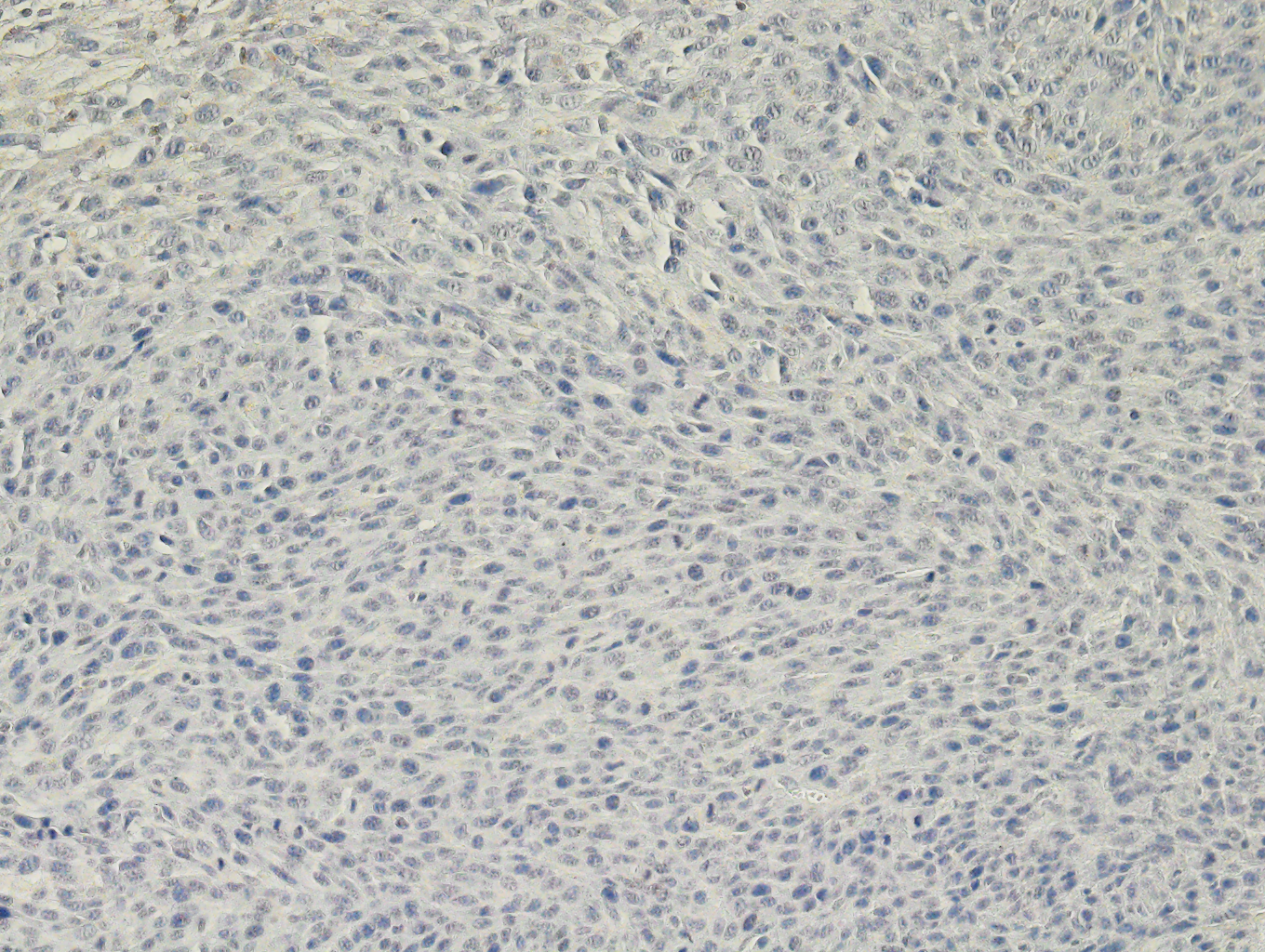

Supplement: Supplementary file 15 — Source data Fig. 6 [file 44319_2025_521_MOESM15_ESM.zip › Figure 6/6F/p-S6 DMSO.tif]

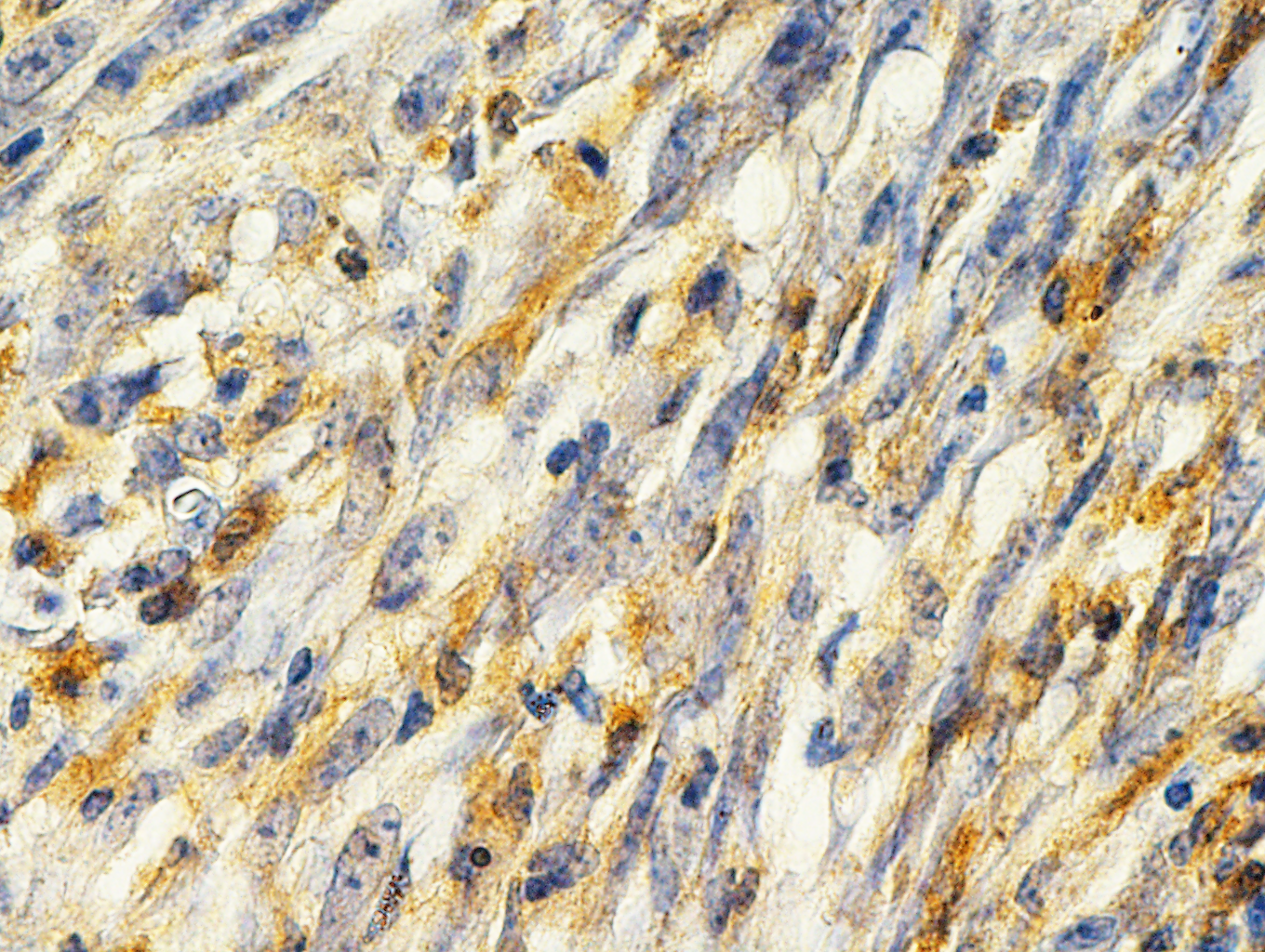

Supplement: Supplementary file 15 — Source data Fig. 6 [file 44319_2025_521_MOESM15_ESM.zip › Figure 6/6F/p-S6 PLX4032-100C.tif]

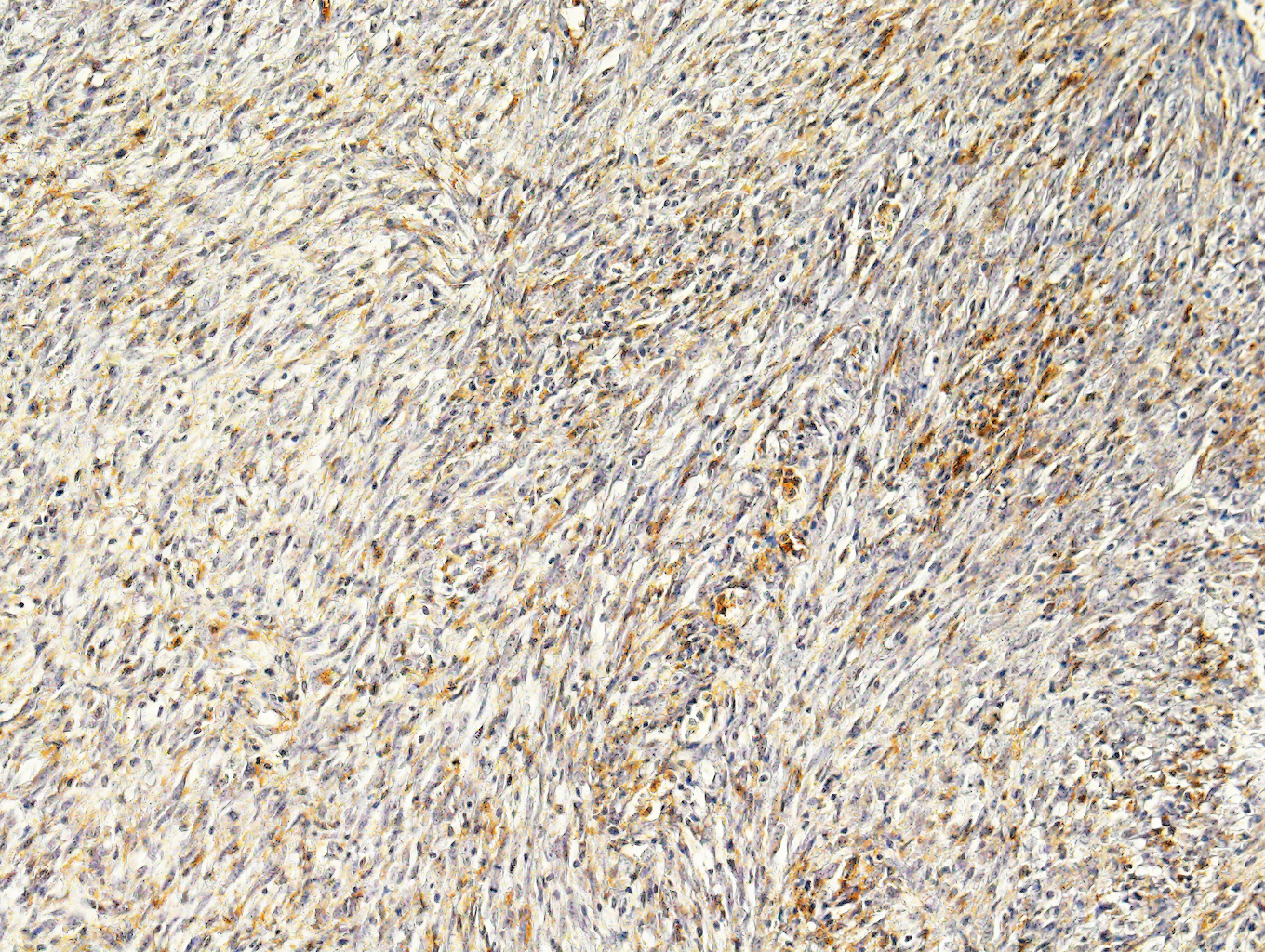

Supplement: Supplementary file 15 — Source data Fig. 6 [file 44319_2025_521_MOESM15_ESM.zip › Figure 6/6F/p-S6 PLX4032.tif]

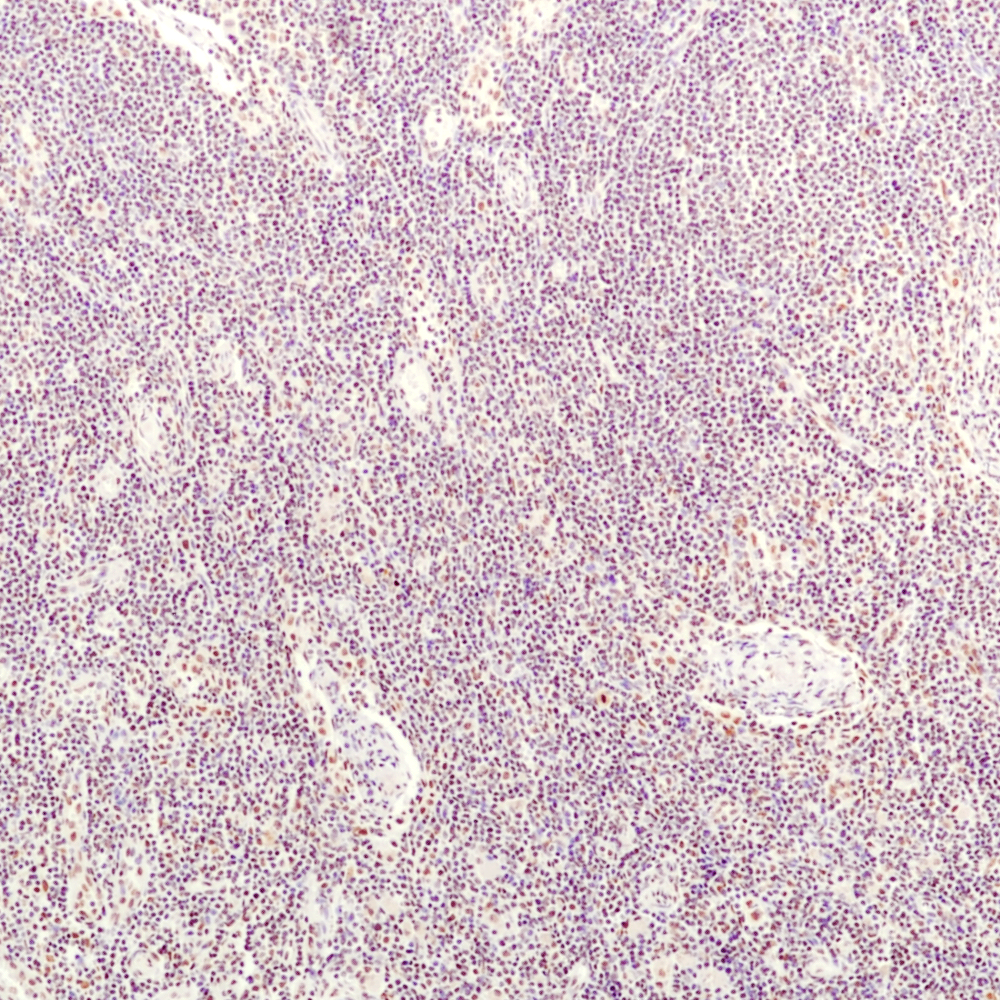

Supplement: Supplementary file 15 — Source data Fig. 6 [file 44319_2025_521_MOESM15_ESM.zip › Figure 6/6H/Pt1 hnRNPK-1 Baseline.png]

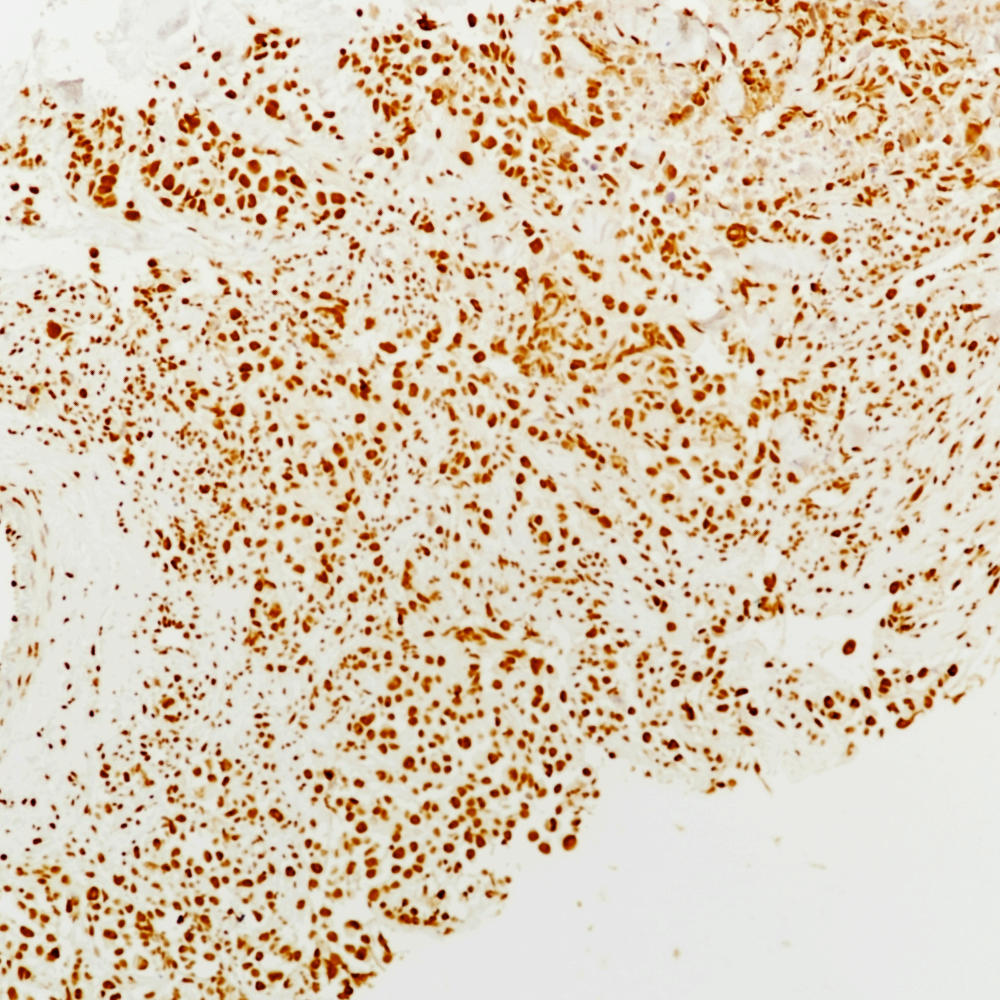

Supplement: Supplementary file 15 — Source data Fig. 6 [file 44319_2025_521_MOESM15_ESM.zip › Figure 6/6H/Pt1 hnRNPK-1 DP.png]

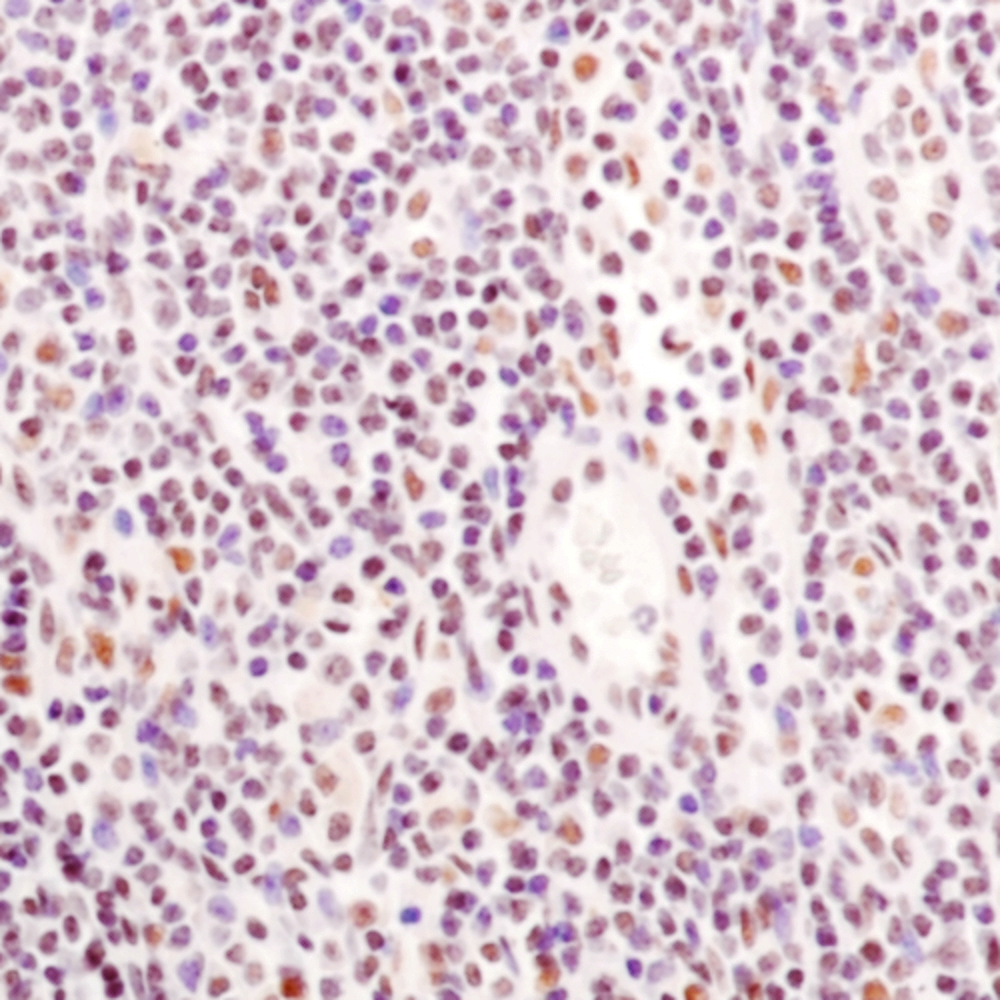

Supplement: Supplementary file 15 — Source data Fig. 6 [file 44319_2025_521_MOESM15_ESM.zip › Figure 6/6H/Pt1 hnRNPK-2 Baseline.png]

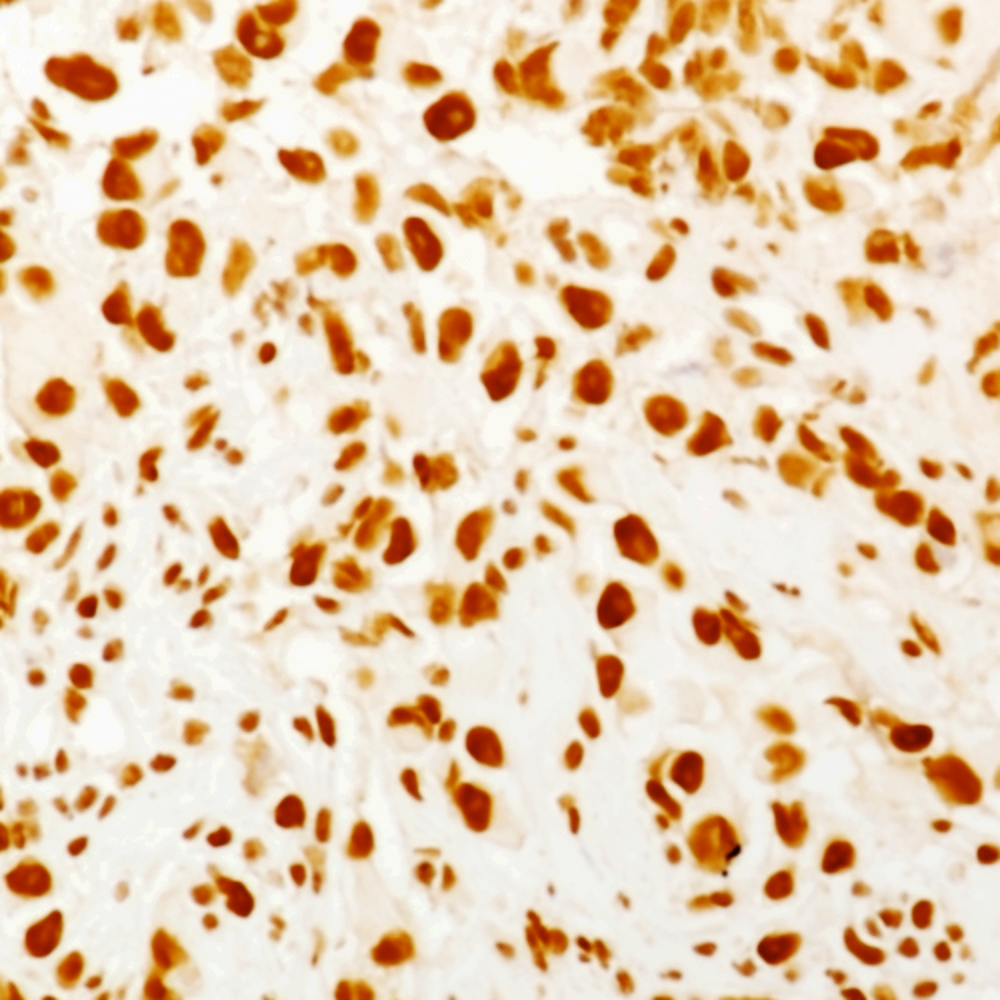

Supplement: Supplementary file 15 — Source data Fig. 6 [file 44319_2025_521_MOESM15_ESM.zip › Figure 6/6H/Pt1 hnRNPK-2 DP.png]

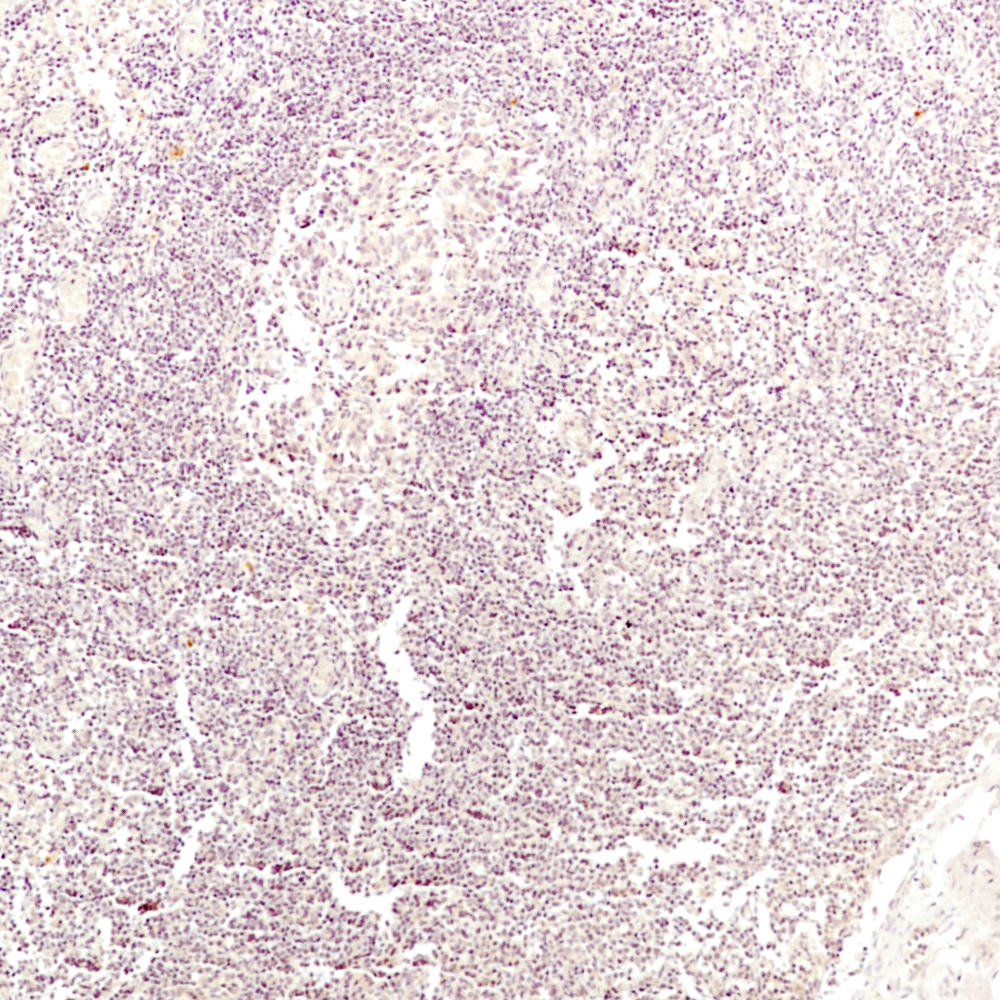

Supplement: Supplementary file 15 — Source data Fig. 6 [file 44319_2025_521_MOESM15_ESM.zip › Figure 6/6H/Pt1 p-AKT-1 Baseline.png]

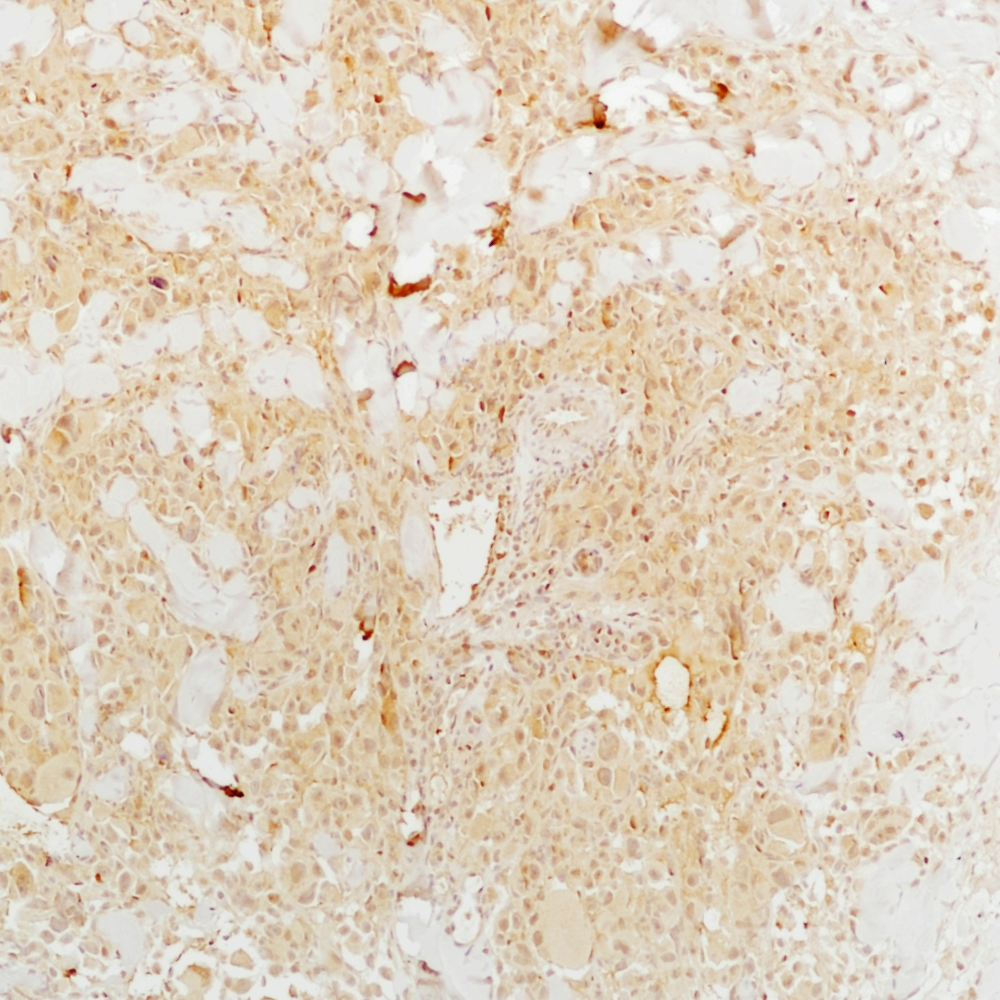

Supplement: Supplementary file 15 — Source data Fig. 6 [file 44319_2025_521_MOESM15_ESM.zip › Figure 6/6H/Pt1 p-AKT-1 DP.png]

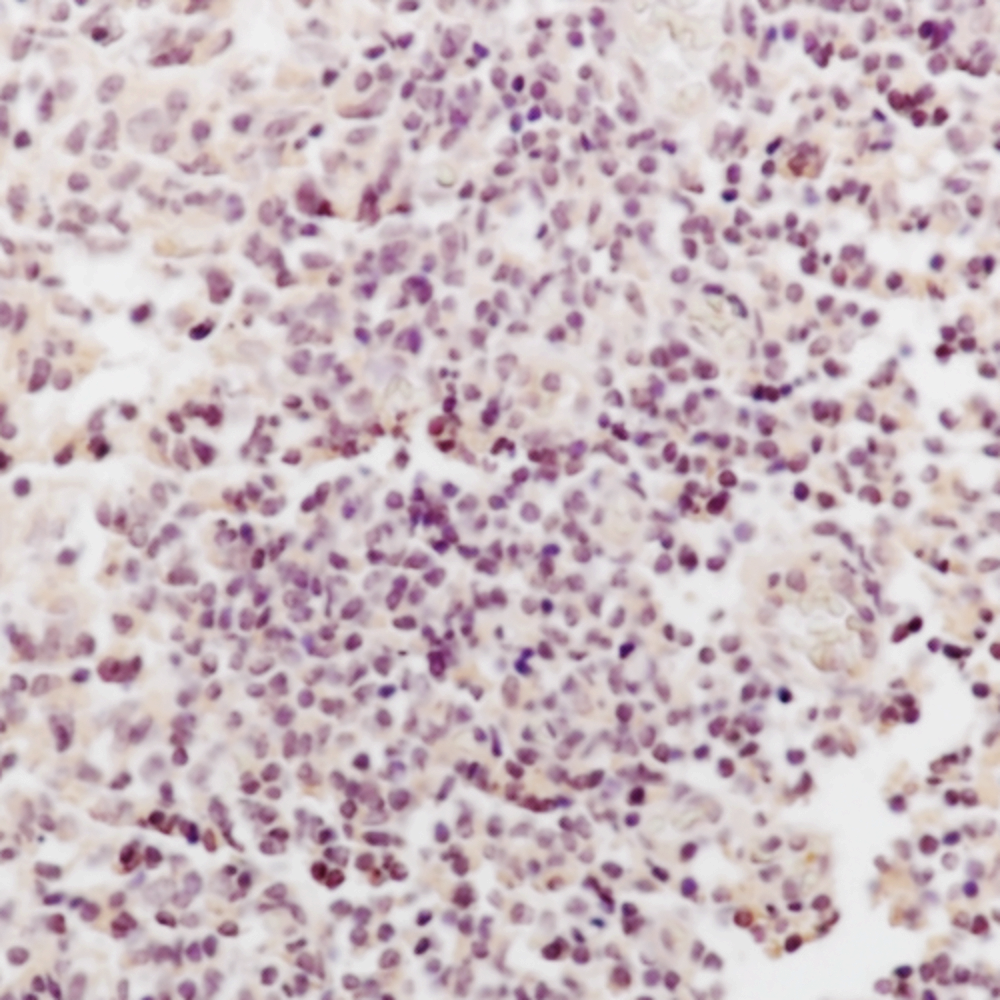

Supplement: Supplementary file 15 — Source data Fig. 6 [file 44319_2025_521_MOESM15_ESM.zip › Figure 6/6H/Pt1 p-AKT-2 Baseline.png]

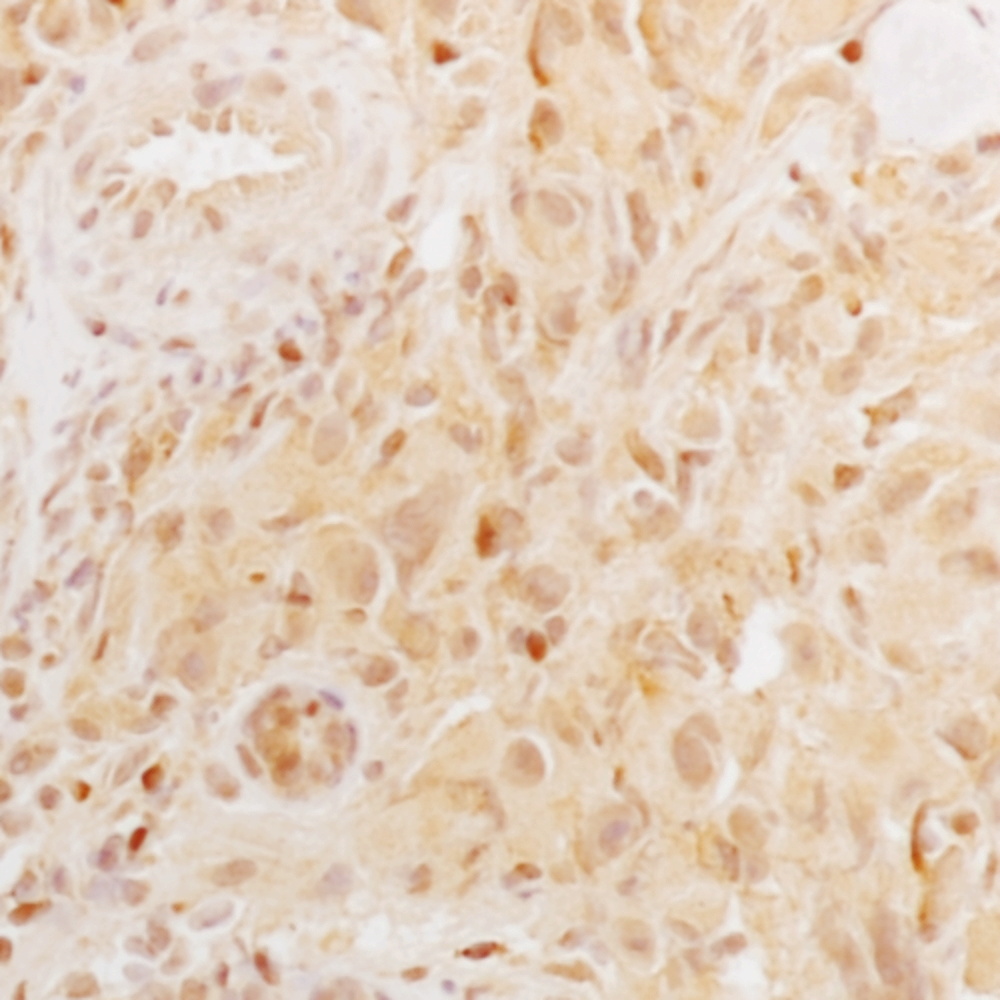

Supplement: Supplementary file 15 — Source data Fig. 6 [file 44319_2025_521_MOESM15_ESM.zip › Figure 6/6H/Pt1 p-AKT-2 DP.png]

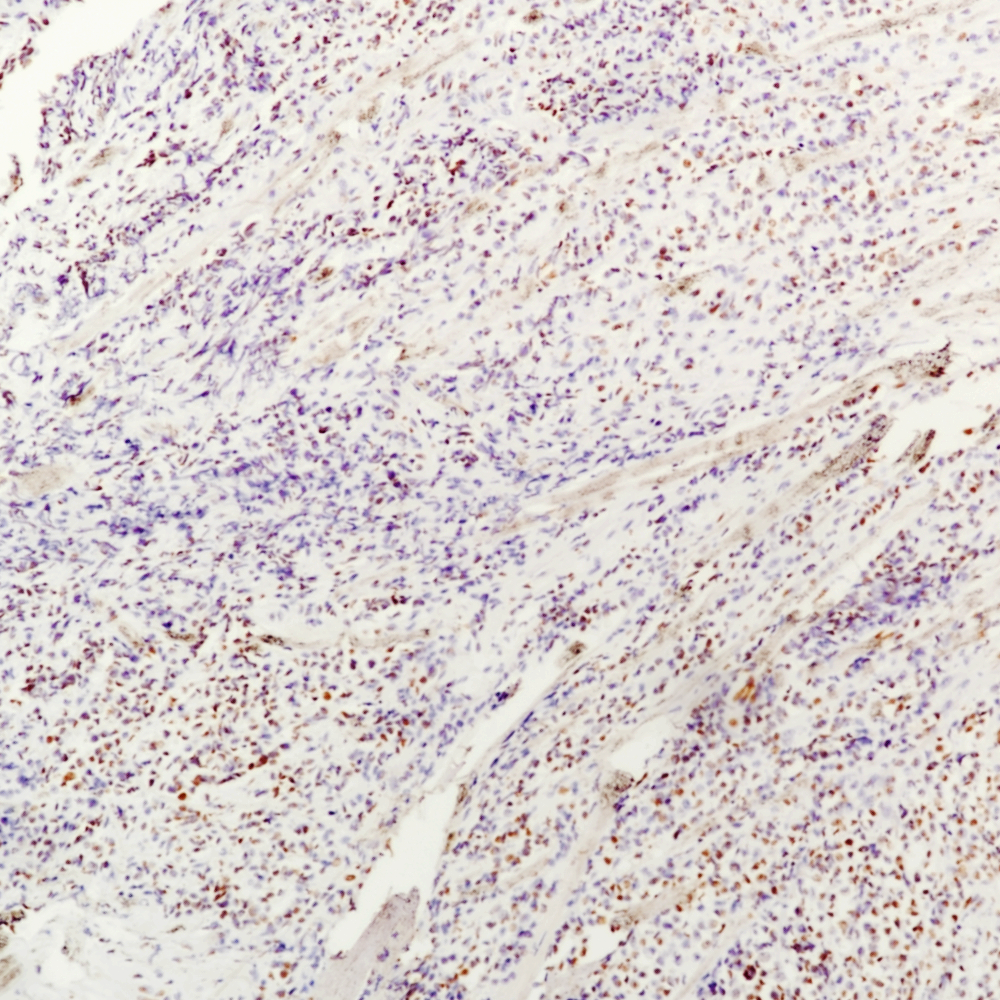

Supplement: Supplementary file 15 — Source data Fig. 6 [file 44319_2025_521_MOESM15_ESM.zip › Figure 6/6H/Pt2 hnRNPK-1 Baseline.png]

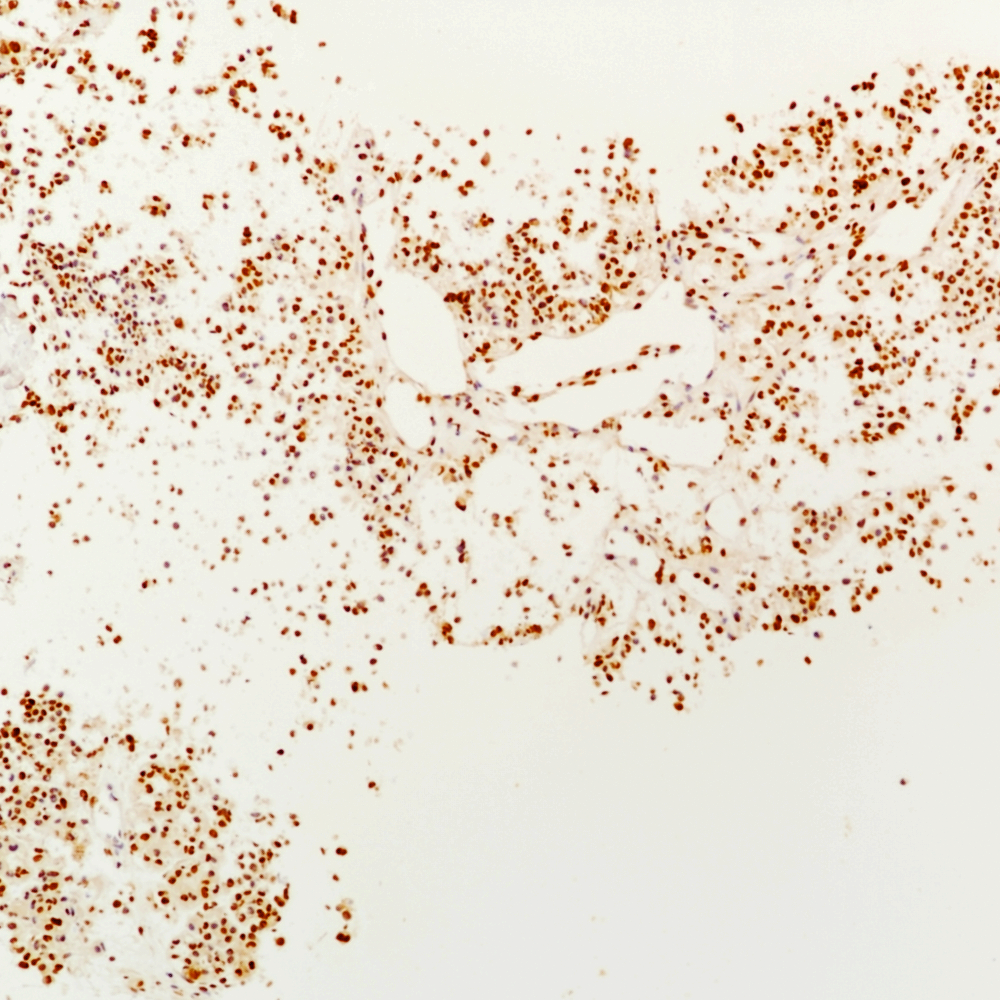

Supplement: Supplementary file 15 — Source data Fig. 6 [file 44319_2025_521_MOESM15_ESM.zip › Figure 6/6H/Pt2 hnRNPK-1 DP.png]

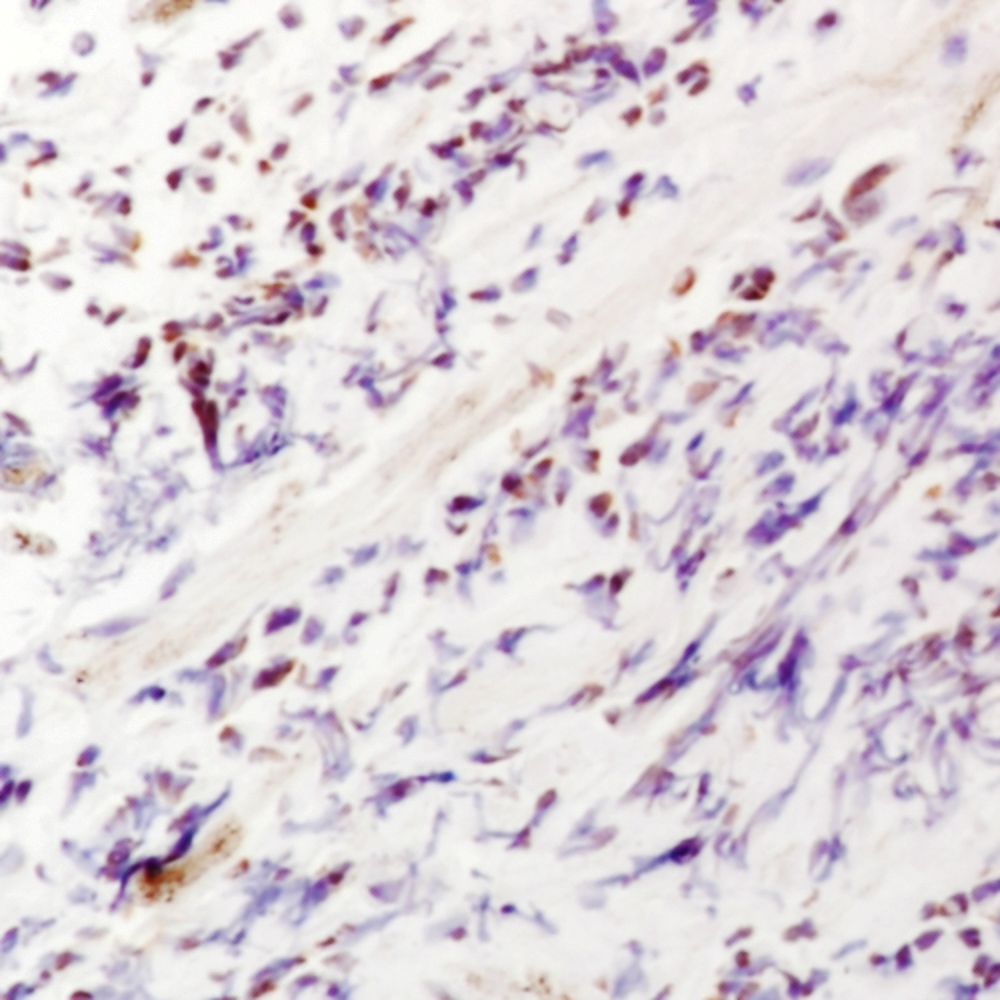

Supplement: Supplementary file 15 — Source data Fig. 6 [file 44319_2025_521_MOESM15_ESM.zip › Figure 6/6H/Pt2 hnRNPK-2 Baseline.png]

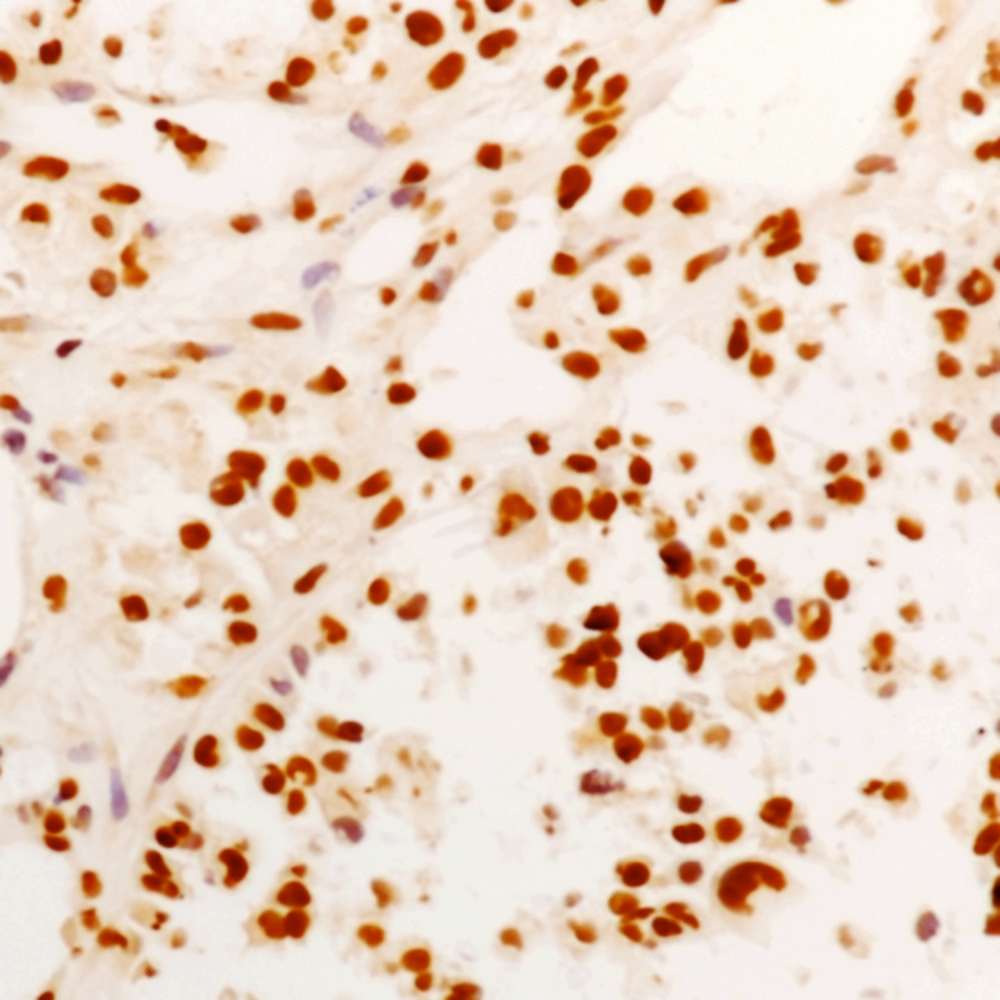

Supplement: Supplementary file 15 — Source data Fig. 6 [file 44319_2025_521_MOESM15_ESM.zip › Figure 6/6H/Pt2 hnRNPK-2 DP.png]

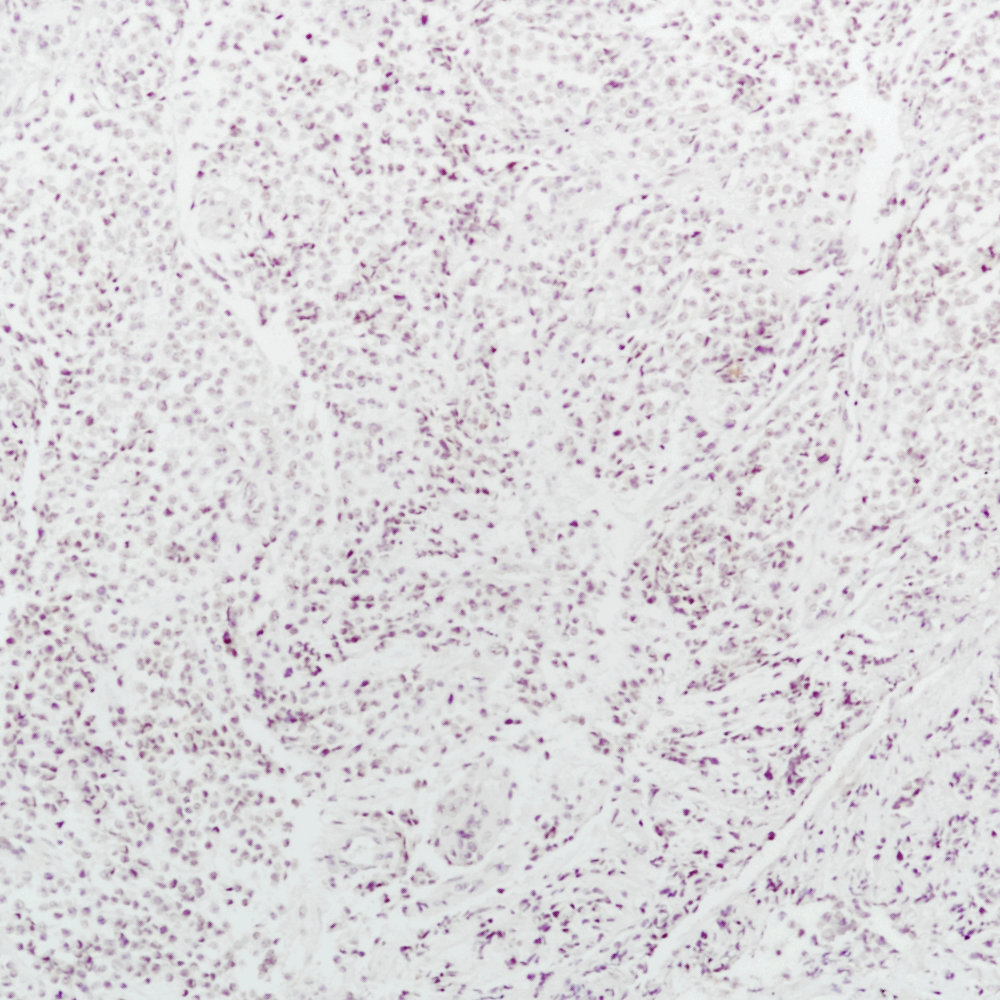

Supplement: Supplementary file 15 — Source data Fig. 6 [file 44319_2025_521_MOESM15_ESM.zip › Figure 6/6H/Pt2 p-AKT-1 Baseline.png]

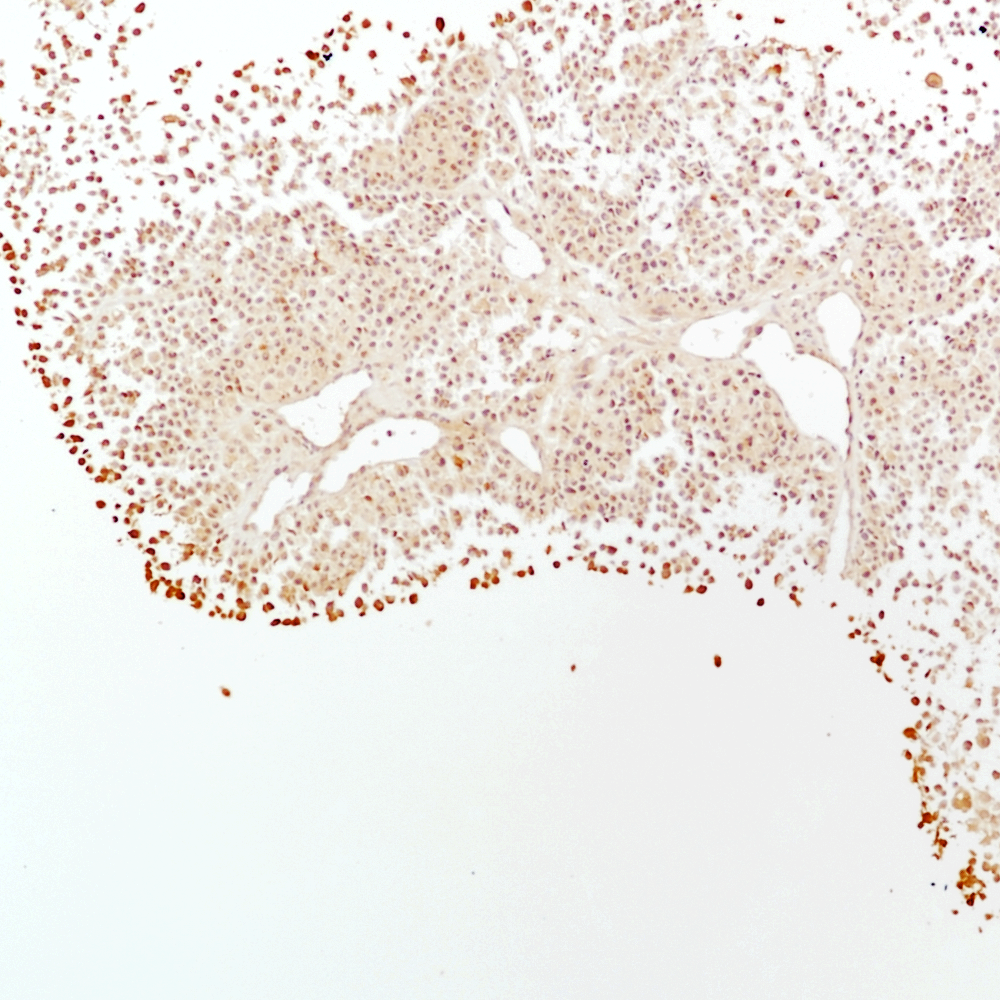

Supplement: Supplementary file 15 — Source data Fig. 6 [file 44319_2025_521_MOESM15_ESM.zip › Figure 6/6H/Pt2 p-AKT-1 DP.png]

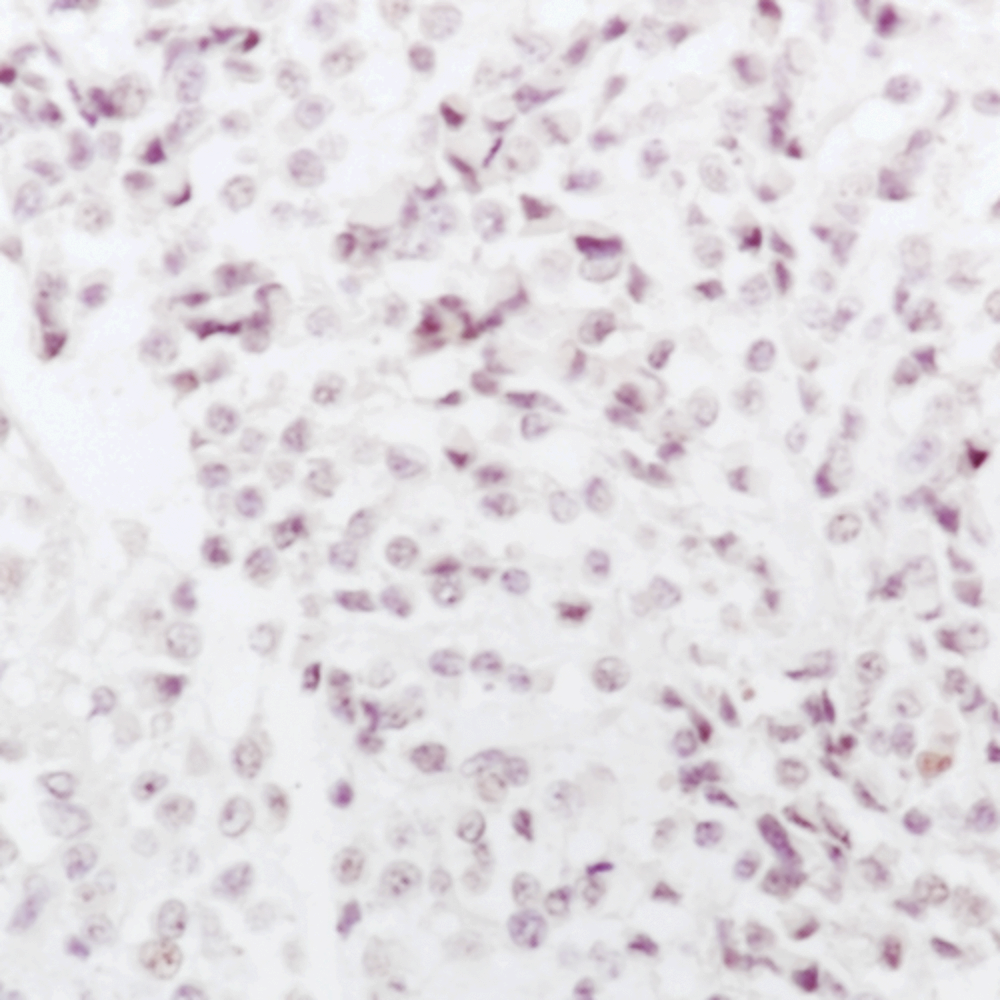

Supplement: Supplementary file 15 — Source data Fig. 6 [file 44319_2025_521_MOESM15_ESM.zip › Figure 6/6H/Pt2 p-AKT-2 Baseline.png]

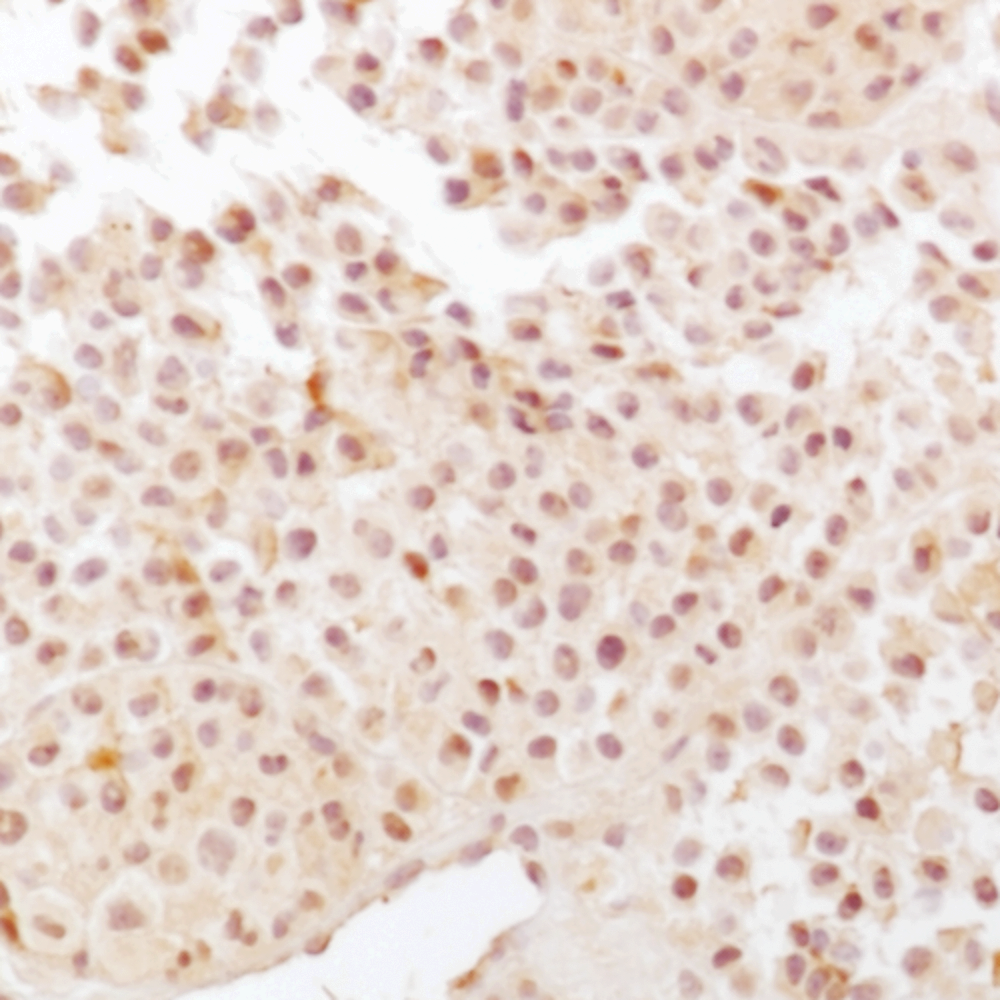

Supplement: Supplementary file 15 — Source data Fig. 6 [file 44319_2025_521_MOESM15_ESM.zip › Figure 6/6H/Pt2 p-AKT-2 DP.png]

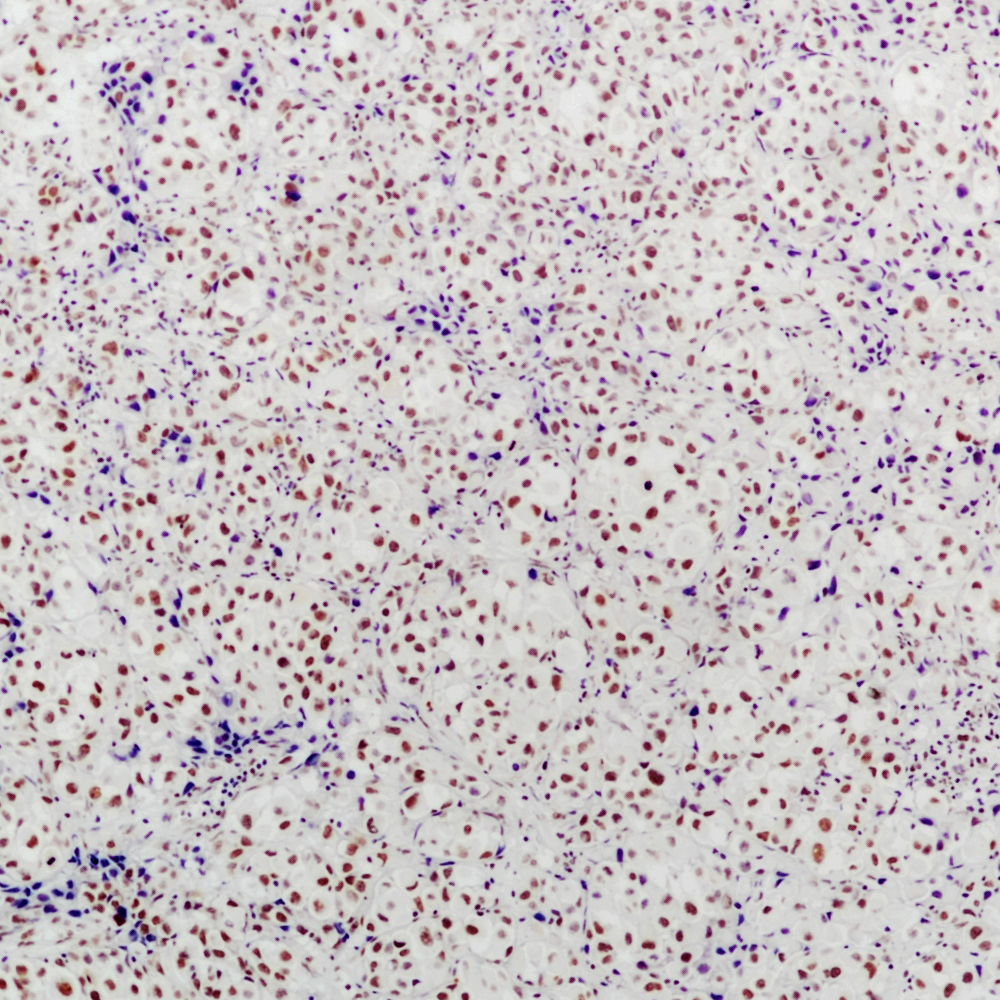

Supplement: Supplementary file 15 — Source data Fig. 6 [file 44319_2025_521_MOESM15_ESM.zip › Figure 6/6H/Pt3 hnRNPK-1 Baseline.png]

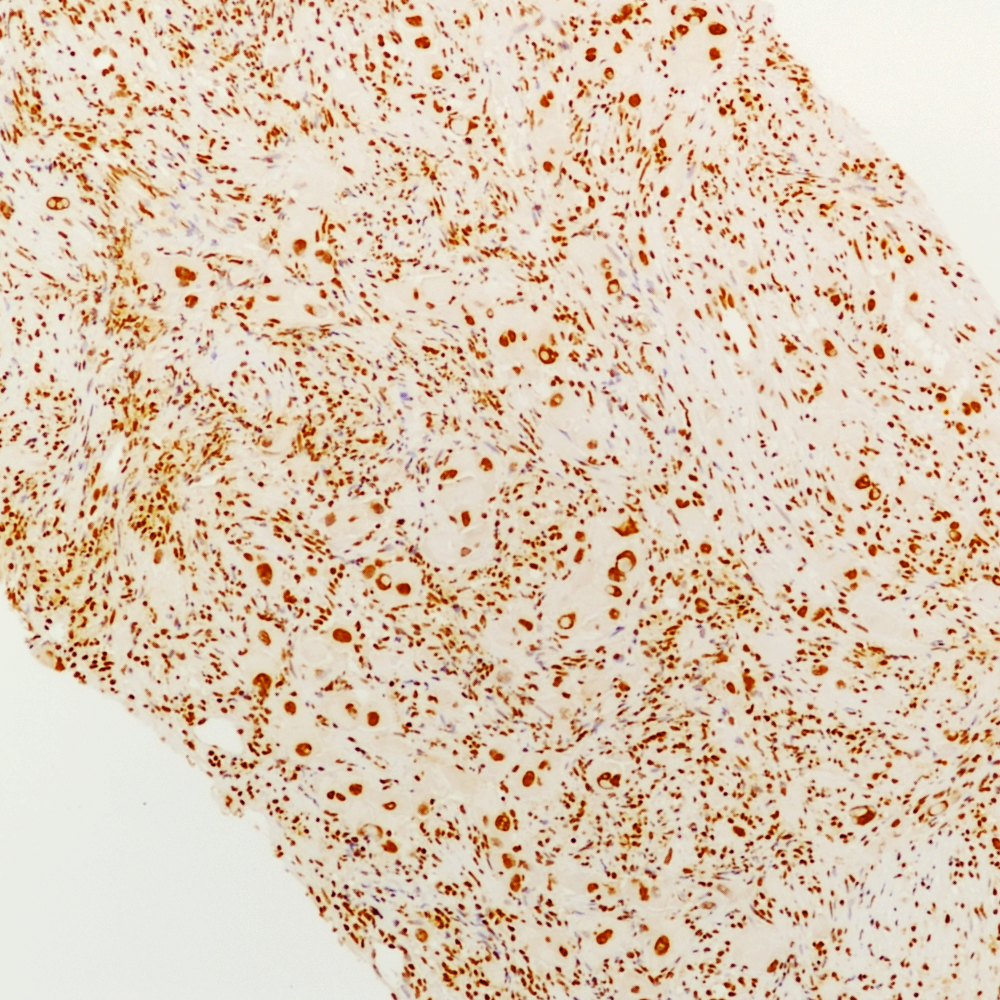

Supplement: Supplementary file 15 — Source data Fig. 6 [file 44319_2025_521_MOESM15_ESM.zip › Figure 6/6H/Pt3 hnRNPK-1 DP.png]

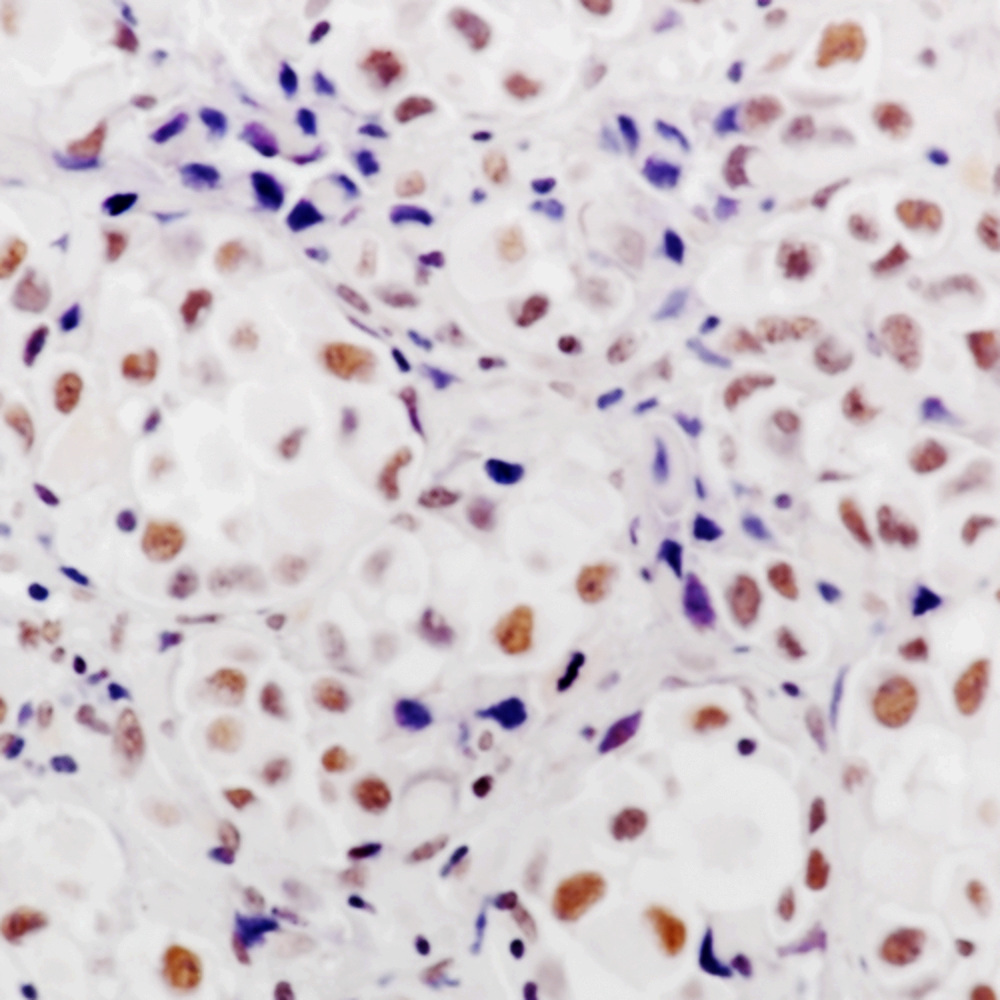

Supplement: Supplementary file 15 — Source data Fig. 6 [file 44319_2025_521_MOESM15_ESM.zip › Figure 6/6H/Pt3 hnRNPK-2 Baseline.png]

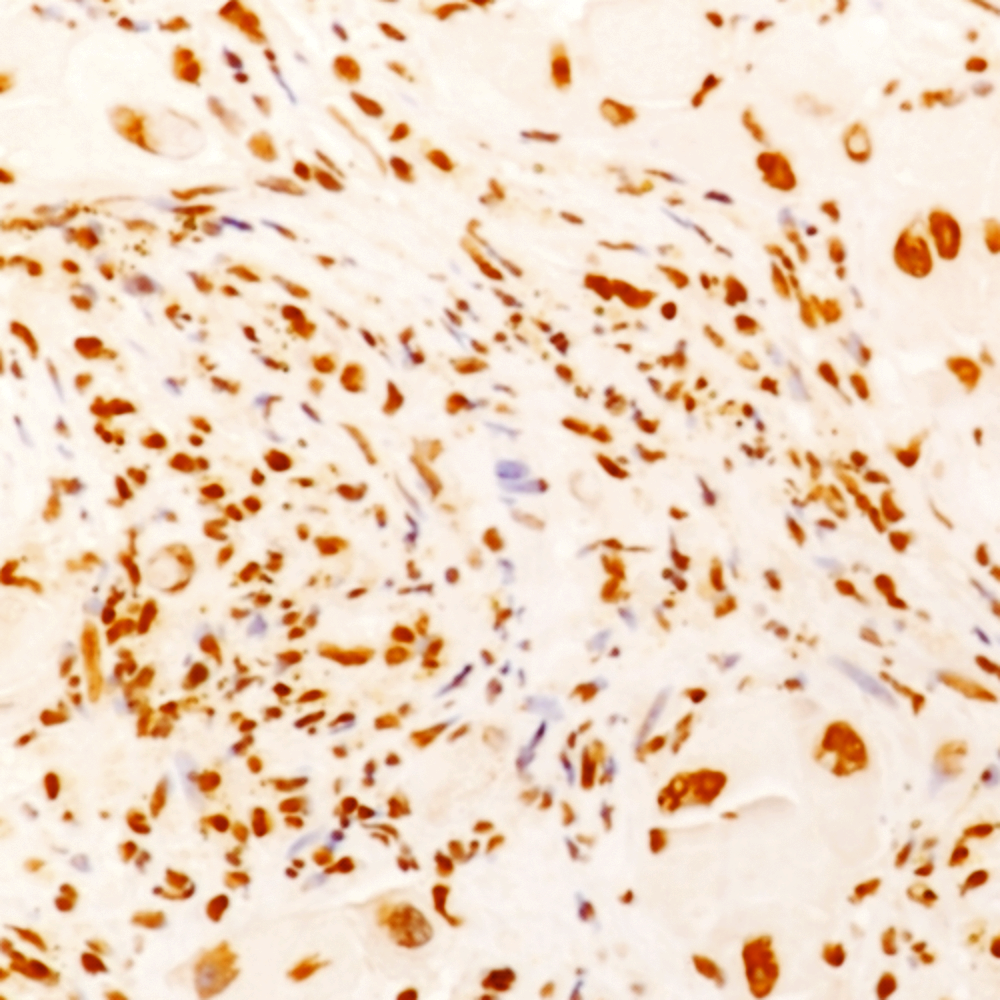

Supplement: Supplementary file 15 — Source data Fig. 6 [file 44319_2025_521_MOESM15_ESM.zip › Figure 6/6H/Pt3 hnRNPK-2 DP.png]

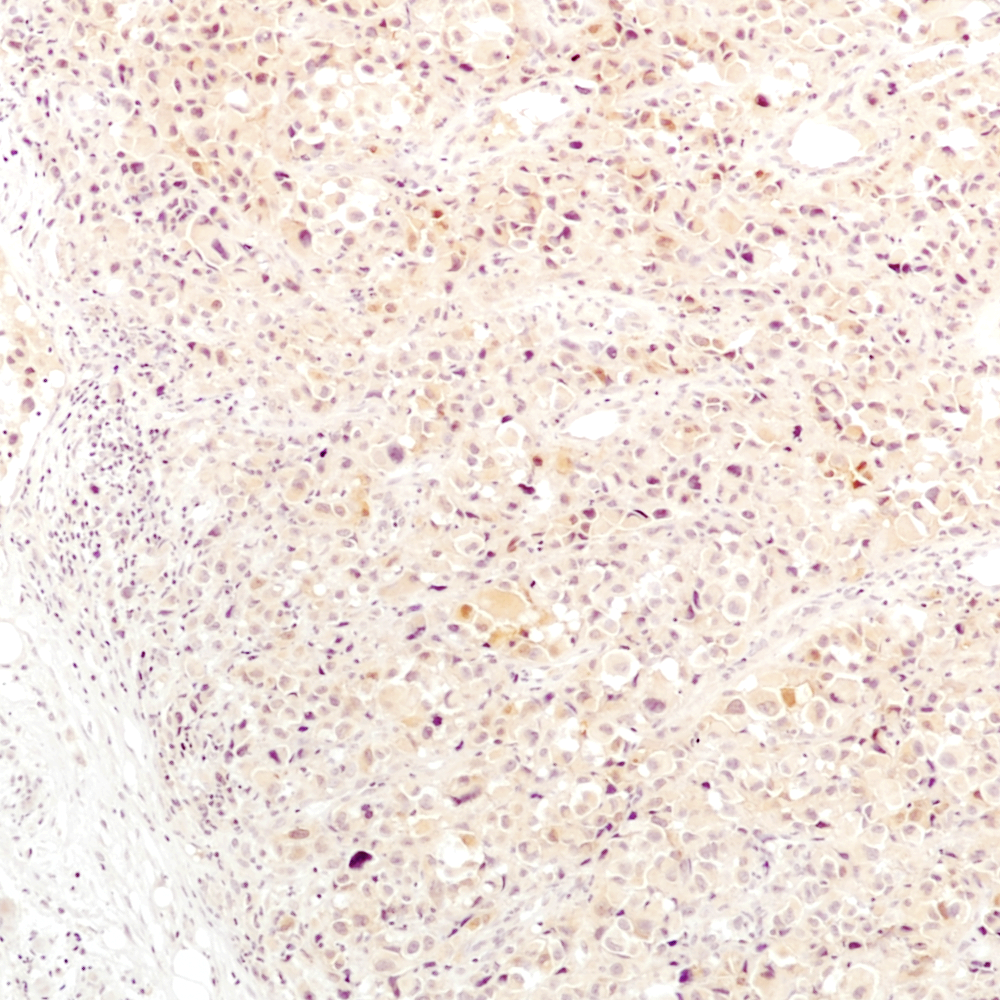

Supplement: Supplementary file 15 — Source data Fig. 6 [file 44319_2025_521_MOESM15_ESM.zip › Figure 6/6H/Pt3 p-AKT-1 Baseline.png]

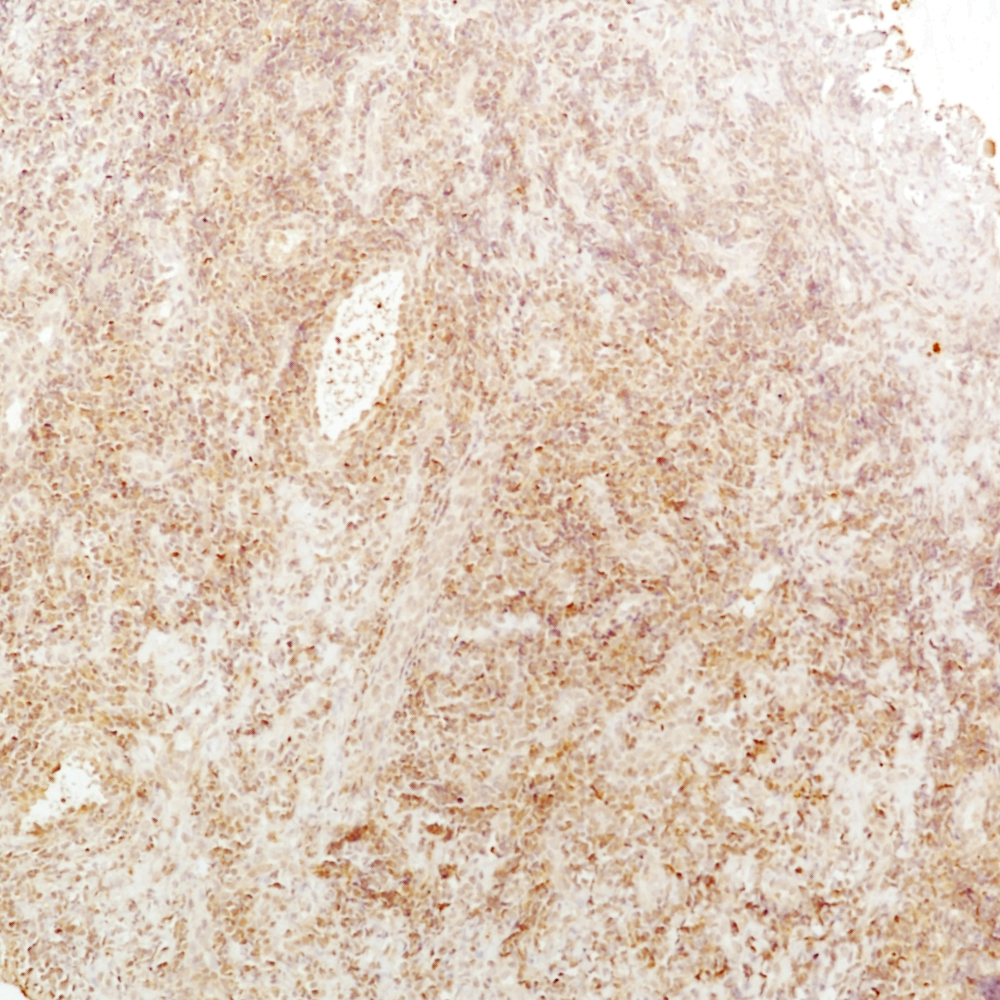

Supplement: Supplementary file 15 — Source data Fig. 6 [file 44319_2025_521_MOESM15_ESM.zip › Figure 6/6H/Pt3 p-AKT-1 DP.png]

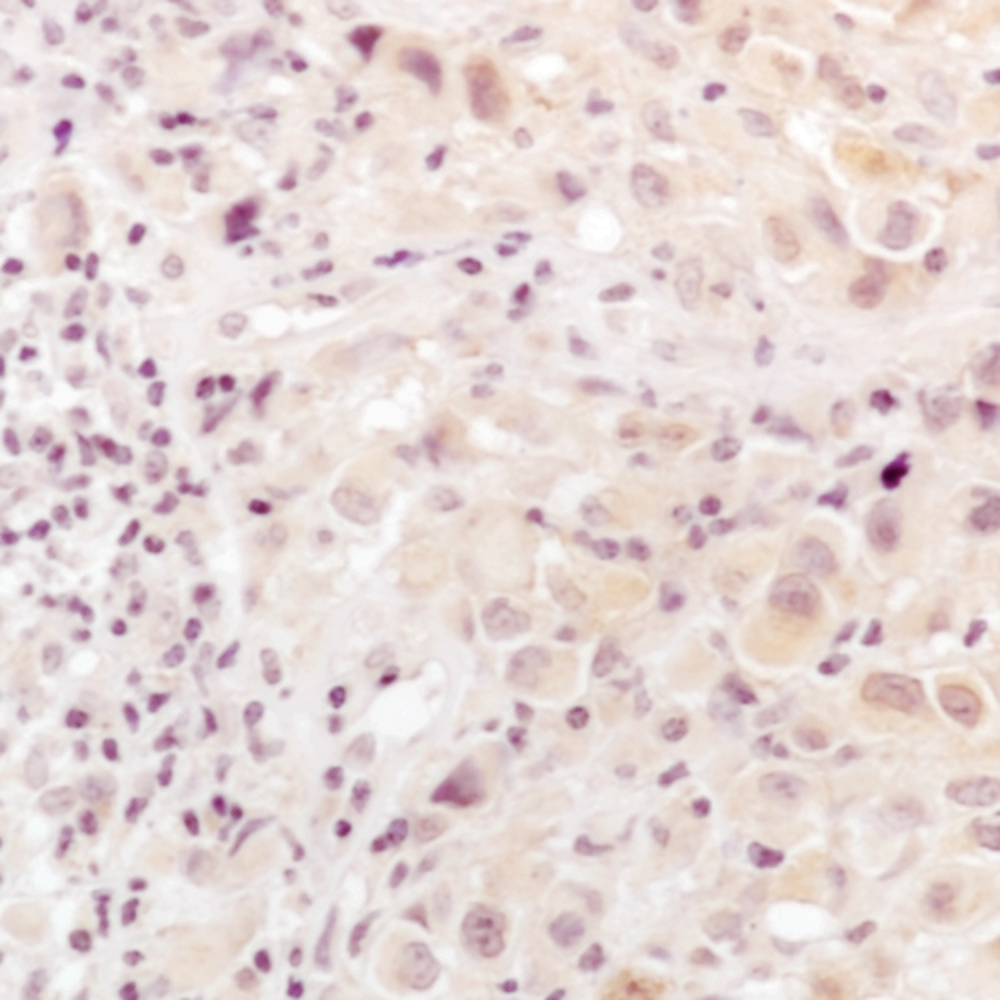

Supplement: Supplementary file 15 — Source data Fig. 6 [file 44319_2025_521_MOESM15_ESM.zip › Figure 6/6H/Pt3 p-AKT-2 Baseline.png]

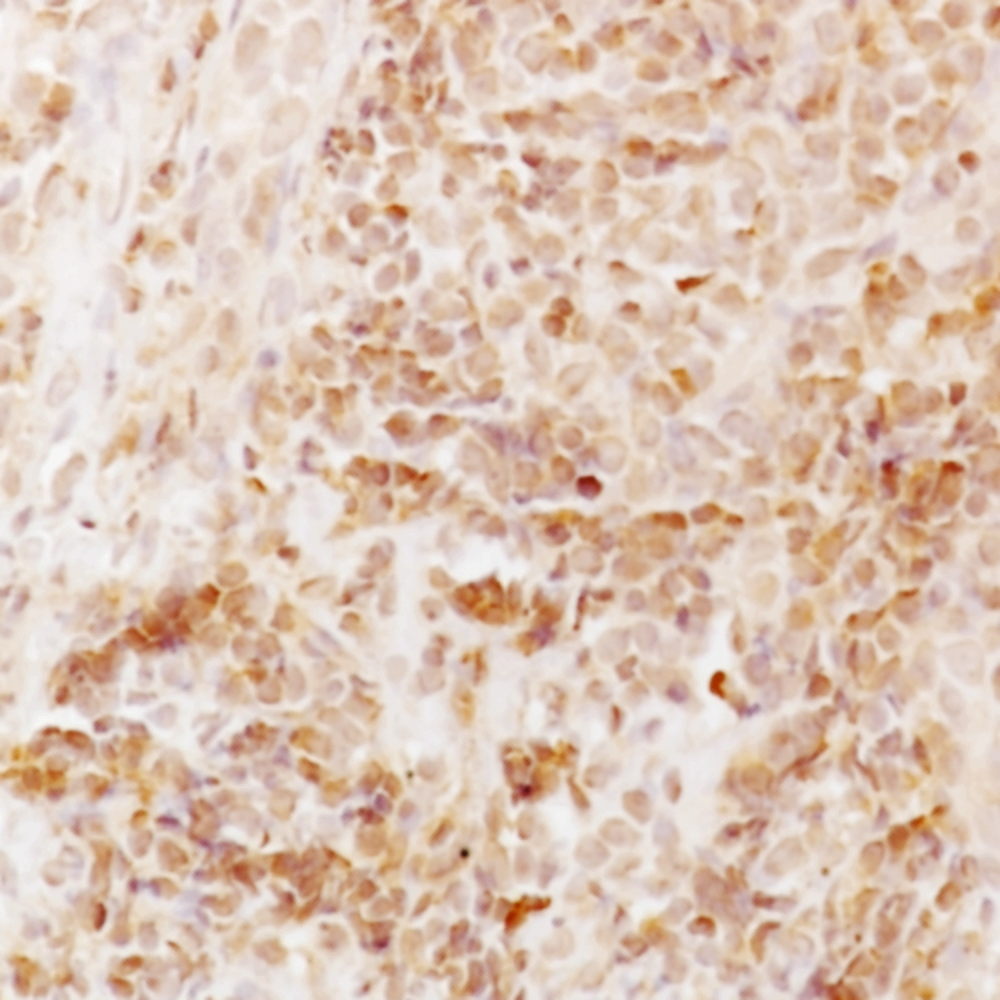

Supplement: Supplementary file 15 — Source data Fig. 6 [file 44319_2025_521_MOESM15_ESM.zip › Figure 6/6H/Pt3 p-AKT-2 DP.png]
